# s-Block Metal Catalysts for the Hydroboration of Unsaturated Bonds

**DOI:** 10.1021/acs.chemrev.1c00641

**Published:** 2022-03-07

**Authors:** Marc Magre, Marcin Szewczyk, Magnus Rueping

**Affiliations:** † Institute of Organic Chemistry, RWTH Aachen University, Landoltweg 1, 52074 Aachen, Germany; ‡ Chemical Science Program, Physical Science and Engineering Division, King Abdullah University of Science and Technology (KAUST), KAUST Catalysis Center, Thuwal 23955-6900, Kingdom of Saudi Arabia; Institute of Experimental Molecular Imaging, RWTH Aachen University, Forckenbeckstrasse 55, 52074 Aachen, Germany

## Abstract

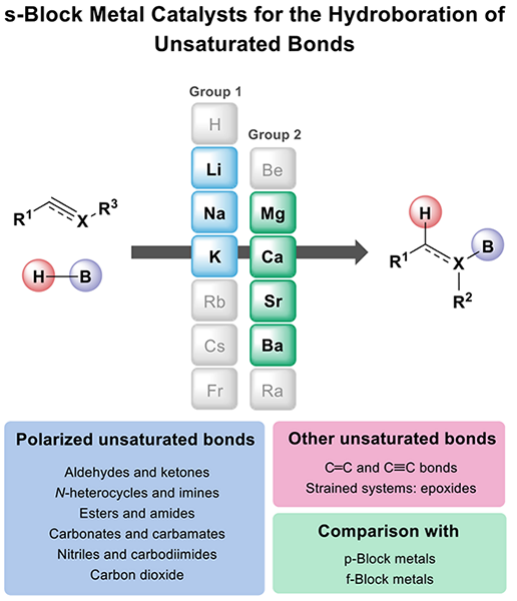

The addition of a B–H bond to an unsaturated bond (polarized or unpolarized) is a powerful and atom-economic tool for the synthesis of organoboranes. In recent years, s-block organometallics have appeared as alternative catalysts to transition-metal complexes, which traditionally catalyze the hydroboration of unsaturated bonds. Because of the recent and rapid development in the field of hydroboration of unsaturated bonds catalyzed by alkali (Li, Na, K) and alkaline earth (Mg, Ca, Sr, Ba) metals, we provide a detailed and updated comprehensive review that covers the synthesis, reactivity, and application of s-block metal catalysts in the hydroboration of polarized as well as unsaturated carbon–carbon bonds. Moreover, we describe the main reaction mechanisms, providing valuable insight into the reactivity of the s-block metal catalysts. Finally, we compare these s-block metal complexes with other redox-neutral catalytic systems based on p-block metals including aluminum complexes and f-block metal complexes of lanthanides and early actinides. In this review, we aim to provide a comprehensive, authoritative, and critical assessment of the state of the art within this highly interesting research area.

## 1 Introduction

**Scheme 1:**
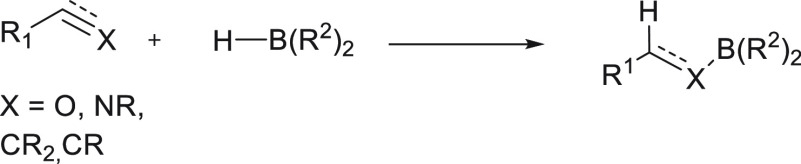
Hydroboration of Unsaturated Bonds

Hydroboration—the addition of a boron–hydrogen bond to an unsaturated bond—is a useful and atom-economic transformation for the synthesis of organoboranes. The addition of boranes (HBR_2_) to C=O or C=N bonds (Scheme 1) results in formation of a boron–oxygen or nitrogen bond, which, along with hydrolysis, constitutes a two-step process equivalent to reduction.^1,2^ Furthermore, the addition of boranes to carbon–carbon unsaturated bonds (*e.g.*, alkenes or alkynes) results in the synthesis of a carbon–boron bond suitable for sequential transformations such as C–C couplings^3^ (*i.e.*, Suzuki coupling reaction^4,5^).

**Scheme 2:**
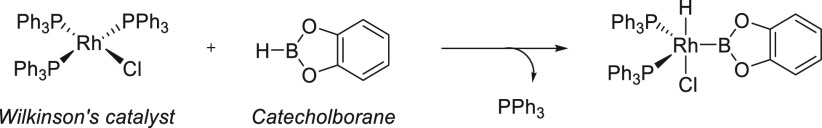
H–B Bond Activation by Wilkinson’s Catalyst

In 1956, Brown *et al.* discovered the direct addition of a B–H bond across a C=C bond using sodium borohydride-aluminum chloride mixtures.^6^ This pioneering discovery marked a substantial breakthrough in the hydroboration reaction.^7^ Using the simplest borane (BH_3_), the transformation occurs spontaneously without the need for a catalyst to provide the *anti*-Markovnikov product. In this regard, the regioselectivity observed is rationalized as follows: (i) B–H addition occurs in a *cis*-fashion; (ii) the boryl moiety prefers the least sterically hindered carbon; and (iii) the hydridic character of the H–B bond favors interactions with an electropositive carbon in the transition state of the reaction.^8,9^ Catecholborane (HBcat) and pinacolborane (HBpin) have emerged as alternatives to highly reactive BH_3_. Groundbreaking work by Kono and Ito *et al.* revealed that Wilkinson’s catalyst, Rh(PPh_3_)_3_Cl, underwent oxidative addition with HBcat (Scheme 2),^10^ which led to the first example of metal-catalyzed hydroboration of alkenes and alkynes by Männig and Nöth *et al.*(11)

Since then, research on transition-metal-based catalytic systems capable of providing hydroboration chemo- and regioselectively has increased exponentially.^12,13^ Early-transition-metal,^14,15^ first-row metal,^16,17^ and lanthanide complexes^18−20^ have also shown excellent activity and selectivity toward the hydroboration of polarized and unpolarized bonds. However, these materials have the drawbacks of high cost and toxicity. To overcome these problems, s-block metals have been applied as more sustainable alternatives.

**Figure 1:**
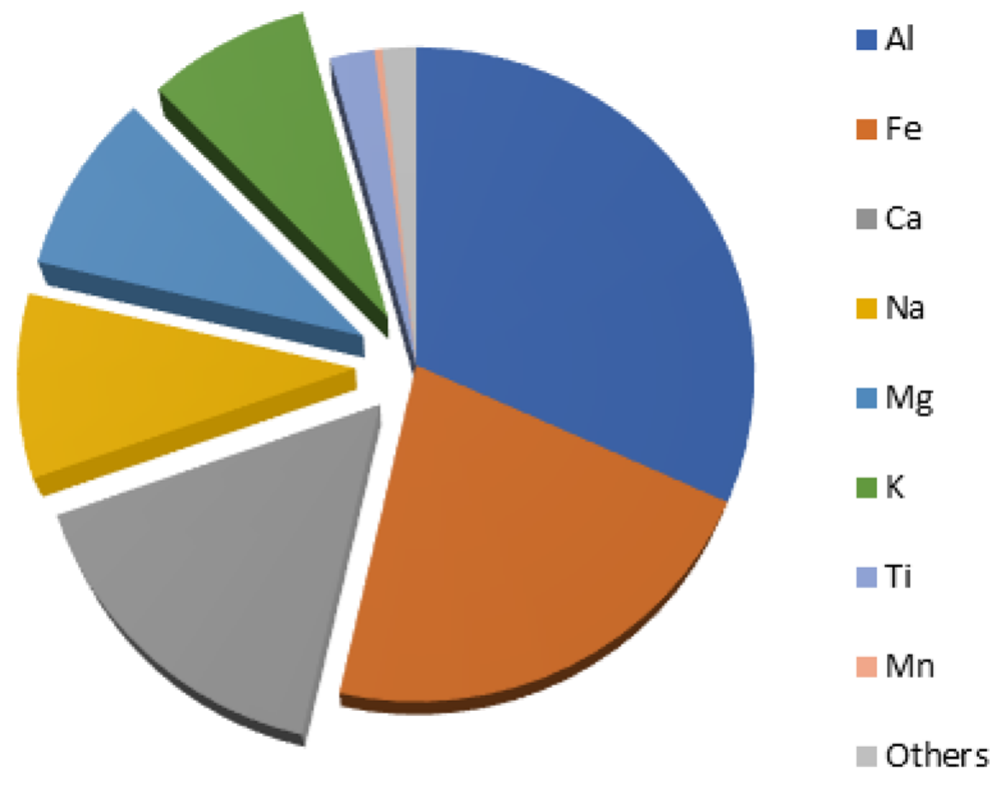
Relative abundance of metals in the Earth’s crust. Alkali and alkaline earth metals are highlighted.

In the past decade, the application of alkaline earth metals and, more recently, alkali metals has evolved rapidly.^21^ Among the latest discoveries, the use of these metal complexes in catalysis, which was not thought to be possible merely two decades ago, stands out. With these breakthroughs, interest in the catalytic activity of group 1 and group 2 metals has increased tremendously.^22,23^ Although some alkaline earth metals, such as magnesium and calcium, are among the most abundant metals in the Earth’s crust (Figure 1),^24^ their use in catalysis is still underdeveloped compared to, for instance, that of transition metals.

Organometallic complexes derived from early main group elements are known to be very reactive and difficult to isolate. Their high nucleophilic character and Brønsted basicity make them strong polar reagents. This high reactivity gives them high potential as catalysts for organic transformations that are traditionally catalyzed by transition-metal complexes. In terms of environmental hazards and toxicity, the replacement of expensive and harmful transition metals for abundant and nontoxic alkali and alkaline earth metals is highly desirable, particularly with regard to applications in the pharmaceutical industry or materials synthesis, where residual transition metals must be avoided.

Although the use of early main group metals in hydrofunctionalization catalysis has increased in the past decade,^25^ their application is still fairly limited due to their tendency to undergo Schlenk-type equilibrium (section 2.3).^26^ Whereas group 1 early metals (Li, Na, K) have been mostly limited to hydroboration reactions, group 2 metals (Mg, Ca, Sr, and Ba) have been widely studied and applied in a variety of hydrofunctionalizations of unsaturated bonds.^27−30^

**Scheme 3:**
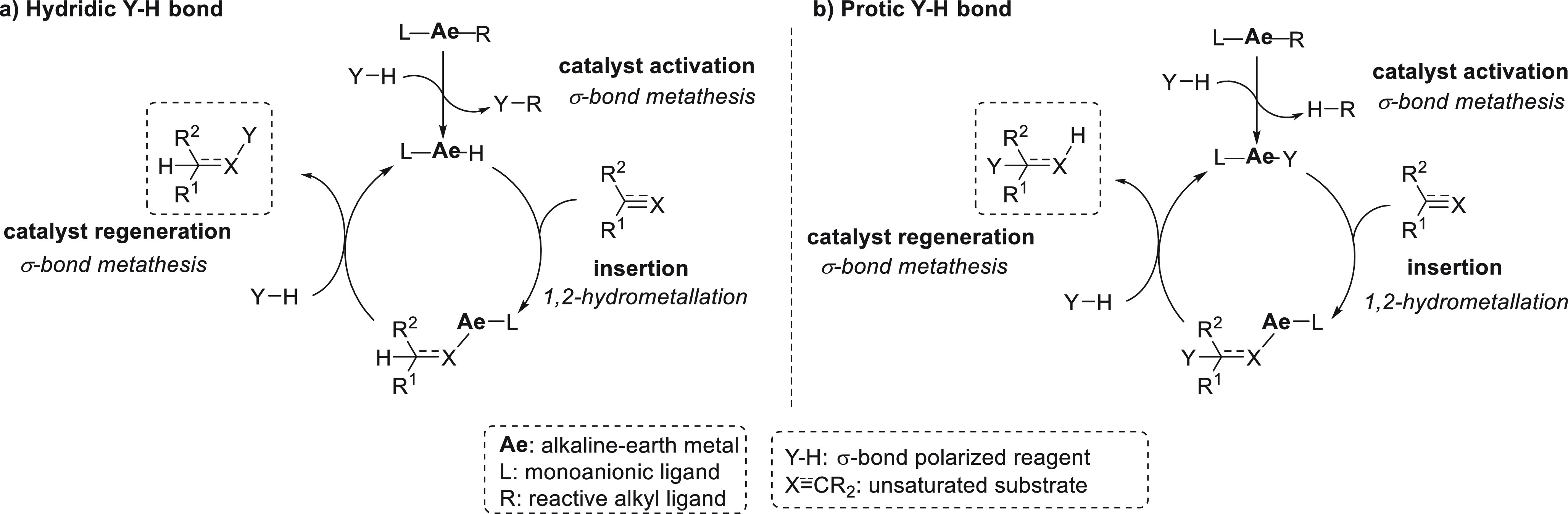
General Catalytic Cycles for s-Block Metal-Catalyzed Hydrofunctionalization of Unsaturated Bonds

In terms of standard reactivity toward hydrofunctionalization of unsaturated bonds, alkaline earth metal catalytic systems differ depending on the polarization of the Y–H bond in the reactant. While hydridic Y–H bonds undergo σ-bond metathesis, protic Y–H bonds undergo protonolysis. Therefore, two general catalytic cycles can be distinguished (Scheme 3).

The year 2016 brought a breakthrough in the area of alkali and alkaline earth metal hydrofunctionalization of unsaturated bonds, specifically, with regard to the hydroboration reaction. Therefore, herein, we will disclose in detail all s-block (group 1 and group 2) metal complexes and their application in the hydroboration of polarized and unpolarized unsaturated bonds.

First, we will briefly describe the different synthetic approaches for the synthesis of alkali- and alkaline earth metal catalysts and their reactivity toward hydroboration reactions (Section 2). Regarding the catalytic applicability, we have organized this section by reactions depending on the nature of the reduced bond: polarized (section 3) and nonpolarized (section 4). As the hydroboration of polarized bonds has been the most studied field, we have organized it in order of applicability: aldehydes and ketones (section 3.1) followed by main C=N bonds such as N-heterocycles and imines (sections 3.2 and 3.3, respectively). Then we will disclose the hydroboration of more stable and thus less reactive compounds such as esters and amides (section 3.4) as well as carbonates and carbamates (section 3.5).

To close the section on polarized unsaturated bonds, we will focus on the hydroboration of other unsaturated systems, such as nitriles and isonitriles (section 3.6), isocyanates and carbodiimides (section 3.7), carbon dioxide (section 3.8), carboxylic acids (section 3.9), and sulfoxides (section 3.10). Finally, we will include hydroboration of C–C double and triple bonds: alkenes (section 4.1), alkynes (section 4.2), and strained ring systems such as epoxides (section 5). For each transformation, we will include and explain in detail all known examples of group 1 and group 2 metal catalysts in chronological order.

Over the past years, aluminum- and lanthanide-based catalysts have been used for hydroboration of organic compounds. Given the similarities in reactivity (Section 6), we decided to provide a comparison of s-block metal-based catalysts with those derived from aluminum (section 6.1) and lanthanides (section 6.2).

Very recently, Thomas *et al.* reported the decomposition of the previously perceived stable hydride source HBPin, a borane most often used in hydroboration reactions. This decomposition results in the formation of BH_3_ which then may act as a “hidden” catalyst (see section 7).^31,32^ We recommend the readers to be aware of this issue while reading our manuscript.

Finally, protocols utilizing a catalyst-free hydroboration approach are briefly discussed in section 7.2.

Please note that the use of heterobimetallic complexes that contain alkali or alkaline earth metals as counterions will be excluded from this review, as they have been recently reviewed by Mulvey *et al.*(33)

## 2 s-Block Organometallic Complexes: Synthesis and Reactivity

In this section, we briefly describe the synthesis and representative examples of alkali and alkaline earth metal complexes applied to the hydroboration of unsaturated bonds. Moreover, the general reactivity trend and catalytic behavior of these complexes will also be discussed.

### 2.1 Synthesis of Alkali Metal Complexes

**Scheme 4:**
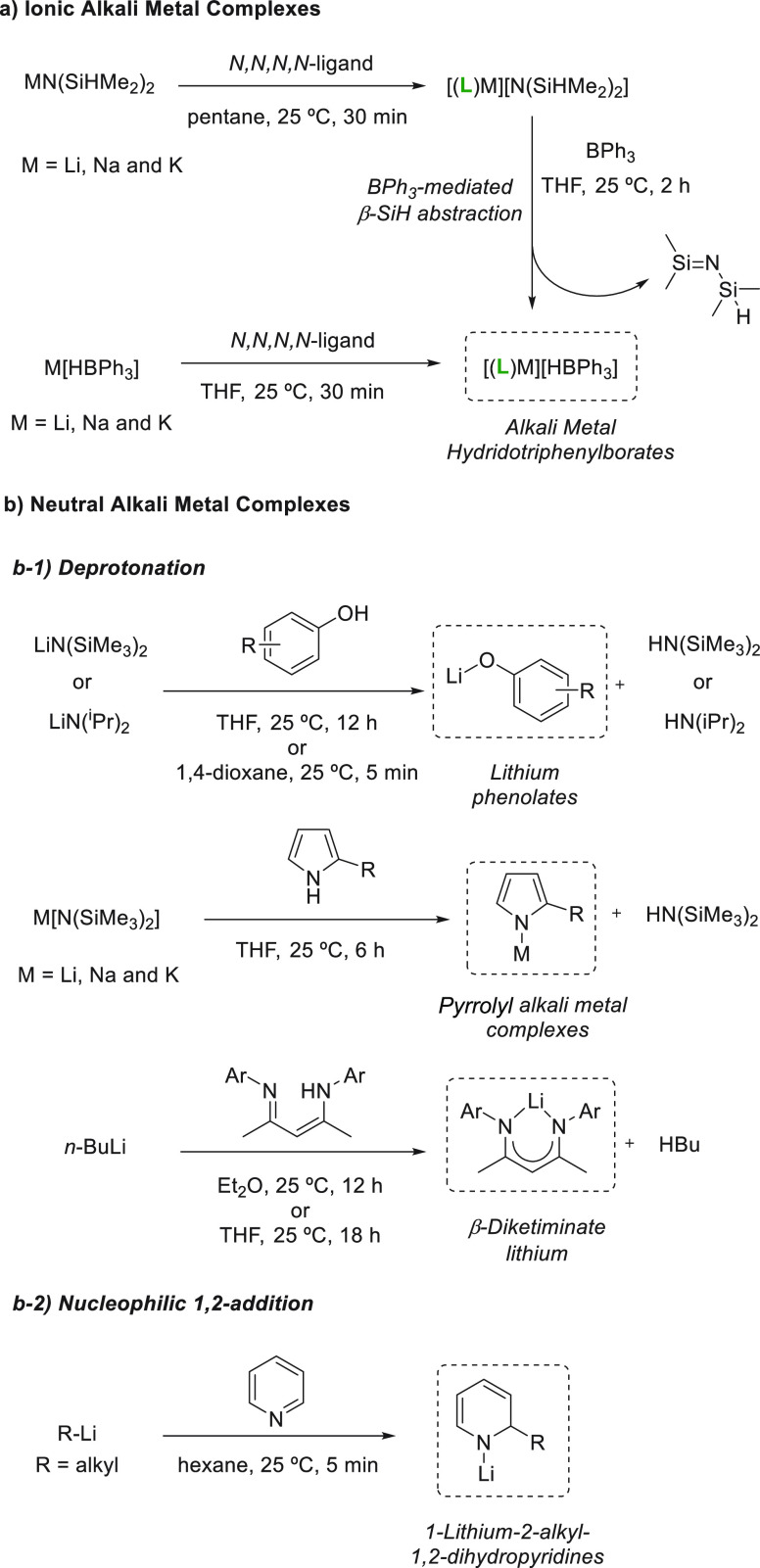
Synthetic Approaches for Alkali Metal Complexes

Although simple and commercially available compounds (*e.g.*, *n*-BuLi, NaOH, and KO*t*-Bu) have been successfully applied in the hydroboration of unsaturated bonds, several alkali metal complexes have also been effectively synthesized and applied in this transformation. In this regard, there are two main synthetic approaches to afford alkali metal complexes: (i) the use of neutral *N,N,N,N*-ligands to form ion-pair complexes (Scheme 4a) and (ii) the formation of neutral alkali metal complexes by either deprotonation of a ligand containing an acidic proton or 1,2-addition of organolithium compounds to pyridines (Scheme 4b).

The first important route (Scheme 4a), explored by Okuda *et al.*, consists of ligand coordination to easily accessible tetramethyl disilazides^34^ followed by BPh_3_-mediated β-SiH abstraction. An alternative route provides group 1 metal complexes after treating the corresponding hydridotriphenylborates with a neutral *N,N,N,N*-ligand (tris{2-(dimethylamino)ethyl}amine). These routes developed by Okuda and co-workers led to the successful synthesis of Li, Na, and K solvent-separated ion pairs.^35−37^

The second approach, which is most commonly used for the synthesis of alkali metal complexes, is based on a deprotonation strategy (Scheme 4b-1). Due to the high basicity of LiN(SiMe_3_)_2_ (p*K*_a_ ∼ 30), lithium diisopropylamide (p*K*_a_ ∼ 35), and *n*-BuLi (p*K*_a_ ∼ 50),^38,39^ these lithium precursors can effectively remove acidic protons from phenol^40^ and pyrrole derivatives,^41^ among others.^42^ In addition, β-diketiminate lithium complexes have been synthesized following the same strategy.^43^

Finally, 1-lithio-2-alkyl-1,2-dihydropyridine complexes can easily be synthesized by nucleophilic addition of alkyl lithium to pyridines, forming soluble and active lithium complexes (Scheme 4b-2). In this regard, Mulvey *et al.* were able to successfully isolate and comprehensively characterize this type of Li complexes.^44^

All of the above-mentioned strategies were applied to the synthesis of active alkali metal catalysts for the hydroboration of unsaturated bonds. All group 1 metal complexes will be presented, and their application will be discussed in section 3.

### 2.2 Synthesis of Alkaline Earth–Metal Complexes

The application of alkaline earth metal complexes in the hydroboration reaction has gained more attention than the application of group 1 metal complexes. For this reason, there are several examples of effective group 2 metal catalysts in the literature. As already described for alkali metal complexes (section 2.1), two main types of alkaline earth metal catalysts can be distinguished: (1) cationic complexes and (2) neutral complexes containing monoanionic or dianionic ligands.

**Scheme 5:**
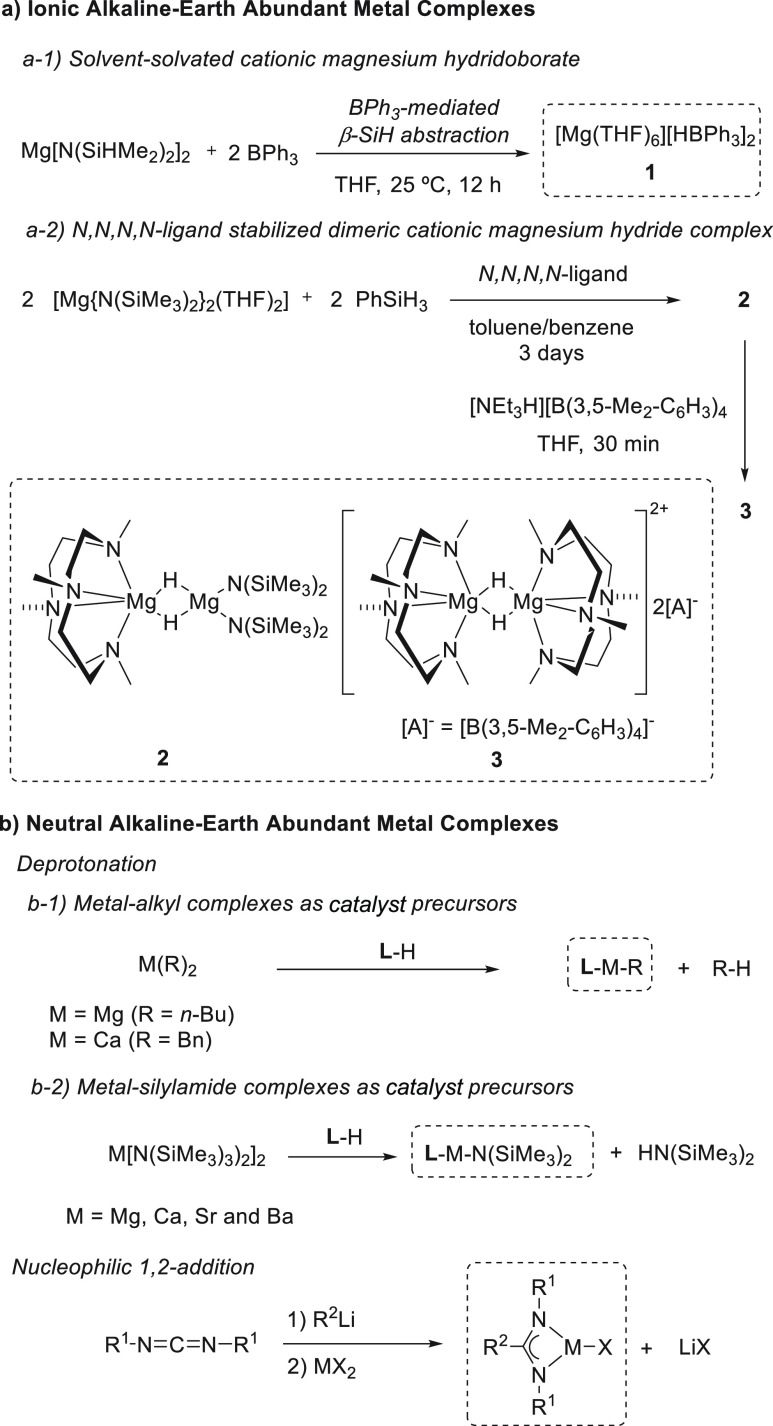
Synthetic Approaches for Alkaline Earth Metal Complexes

The first type, developed by Okuda *et al.*, relies on the synthesis of magnesium hydridotriphenylborate complexes **1** where a coordinative solvent, such as THF, provides the complex as a solvent-separated ion pair (Scheme 5a-1).^45^ Recently, the same authors prepared dimeric cationic magnesium hydride species **2** stabilized by neutral *N,N,N,N*-ligands. The treatment of magnesium bis(hexamethyldisilazide) with the macrocyclic ligand Me_4_TACD (Me_4_TACD = 1,4,7,10-tetramethyl-1,4,7,10-tetraazacyclododecane) and PhSiH_3_ as a hydride source yields magnesium dimeric species **2**, which, after partial protonolysis by [NEt_3_H][B(3,5-Me_2_-C_6_H_3_)_4_] in THF, afforded dimeric hydride ionic species **3** (Scheme 5a-2).^46^

**Figure 2:**
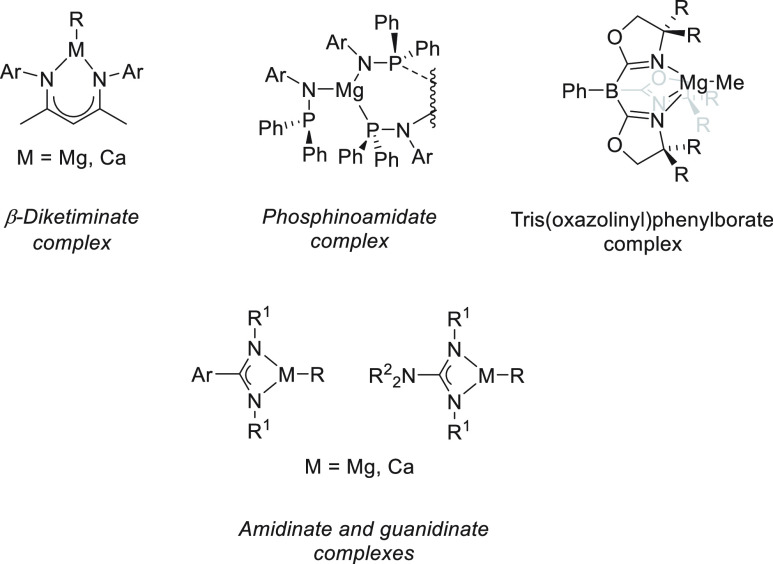
Representative group 2 metal complexes applied in the hydroboration of unsaturated bonds.

The second type of group 2 metal complexes are neutral complexes. Their synthesis is based on a deprotonation strategy of Brønsted acidic ligands by basic metal alkyl or silylamide precursors to afford the corresponding alkaline earth metal complexes (Scheme 5b). This second strategy has been the most widely applied to synthesize neutral complexes. Consequently, there are a wide variety of group 2 metal complexes bearing monoanionic ligands such as β-diketiminates and their derivatives,^47−50^ phosphinoamide,^51^ tris(oxazolinyl)phenylborates and their derivatives,^52^ and guanidinates^53^ or amidinates,^54^ which have been applied in the hydroboration of polarized and unpolarized unsaturated bonds (Figure 2).

The use of monoanionic ligands results in the corresponding stable alkaline earth metal complex bearing a reactive site (*e.g.*, alkyl or silylamide ligands) that reacts with HBpin *via* σ-bond metathesis to afford an active metal hydride species. Moreover, this strong ligand–metal interaction prevents any kind of ligand redistribution, known as Schlenk-type equilibrium,^26^ which would lead to a less reactive species. Although dianionic ligands, such as diols, have been widely used in group 2 metal catalysis,^55−57^ examples of applications in hydroboration reactions are scarce.

Although considerable effort has been made to design and apply tailor-made alkaline earth metal complexes, commercially available or simple Ae-bis(amides),^58−62^*e.g.*, Ae[N(SiMe_3_)_2_]_2_, or Ae-bis(alkyl), *e.g.*, Ae[CH(SiMe_3_)_2_]_2_·(THF)_2_^63^ and Mg(*n*-Bu)_2_,^64^ have also been widely used as precatalysts in other hydrofunctionalizations.

In section 3, all group 2 metal catalysts and their application in hydroboration will be discussed in more detail.

### 2.3 Reactivity of s-Block Organometallic Complexes toward Hydroboration of Unsaturated Bonds

**Figure 3:**
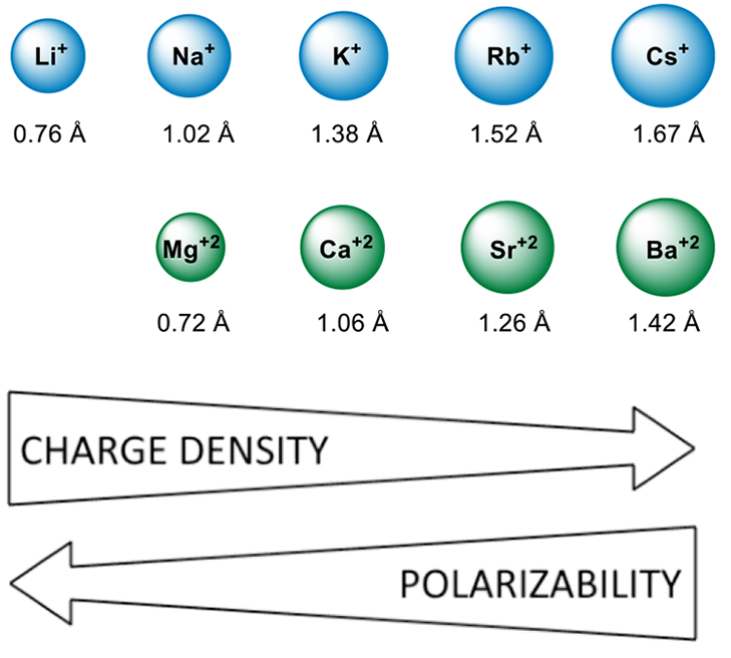
Effective ionic radii of group 1 and group 2 cations.

As previously mentioned, the chemistry of s-block organometallics is marked by their stable +1 (for alkali metals) and +2 (for alkaline earth metals) oxidation states.^65^ In this regard, the ionic radii of the corresponding ions (groups 1 and 2)^66^ increase as the group number decreases, leading to a decrease in electronic density and an increase in polarizability (Figure 3).

**Scheme 6:**
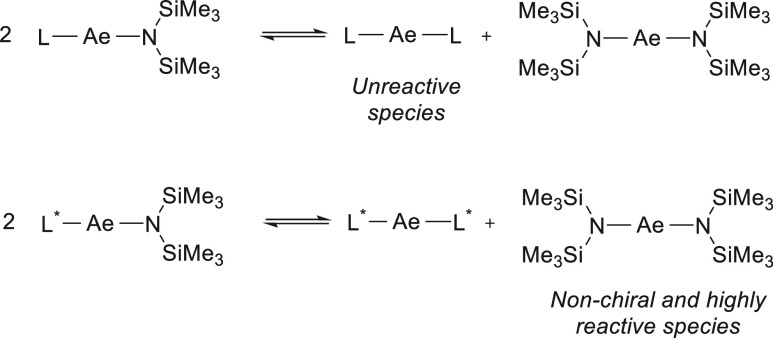
Schlenk-Type Equilibrium

These inherent variations influence the nature of metal-to-ligand bonding. In the case of heavier alkaline earth metals (Ca, Sr, and Ba), the nondirectional ionic interactions affect metal-to-ligand binding. Therefore, heteroleptic complexes tend to undergo Schlenk-type equilibrium,^26^ leading to homoleptic metal complexes, which mostly differ in reactivity (Scheme 6). Concerning enantioselective catalysis, this ligand redistribution can also lead to nonchiral and more reactive homoleptic complexes, which would provide low or no enantioinduction (Scheme 6). To avoid ligand redistribution, considerable efforts have been made in ligand and catalyst design. In this regard, bi- or polydentate monoanionic ligands presenting hard donor sites and steric bulk provide efficient kinetic stability to avoid any kind of ligand redistribution. Alkaline earth metal complexes designed for catalytic hydroboration reactions are frequently based on a spectator ligand (and most of the cases, monoanionic ligand, L) and a reactive ligand (usually silylamide or an alkyl group).

In contrast to transition metals, which usually show reversible oxidation states,^67^ s-block metals generally favor only one oxidation state, a fact that excludes catalytic pathways with redox features such as oxidative addition and reductive elimination.^68^ Therefore, the catalytic steps are simplified and built around basic dipolar transformations.

**Scheme 7:**
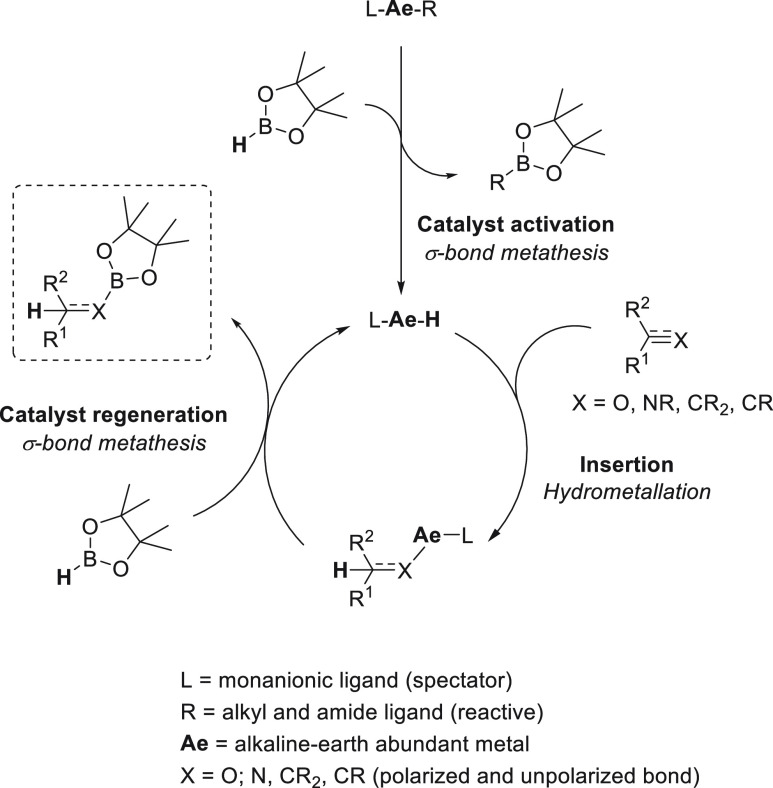
General Catalytic Cycle for the Group 2 Metal-Catalyzed Hydroboration of Unsaturated Bonds

As described in the Introduction, the nature of the hydrofunctionalizing agent determines the elemental catalytic steps (Scheme 3) of the transformation.^69^ In this case, hydroboranes such as HBpin present a hydridic H–B bond; therefore, a general catalytic cycle (Scheme 7) is based on metal hydride^70,71^ bond formation and its addition to an unsaturated bond, as follows: (i) σ-bond metathesis occurs between the polarized catalyst precursor, which bears a reactive labile ligand, and a polarized hydride reagent (H–Bpin). In this first step, a reactive L–Ae–H species is formed. (ii) The metal hydride species is inserted into an unsaturated bond *via* hydrometalation. (iii) Finally, the polarized hydride reagent H–B undergoes σ-bond metathesis to regenerate the active L–Ae–H catalyst and release the corresponding hydroborated product.^72^

This general catalytic cycle, which is based on metal hydride formation and subsequent hydrometalation, can vary depending on the nature of the ligand and the metal. For alkali metal catalysts, which have either spectator monoanionic ligands or reactive ligands, HBpin activation and the catalytic cycle can differ from those described in Scheme 7. Moreover, the hydroboration catalyzed by metal catalysts bearing a dianionic ligand or by a cationic complex also occurs *via* different pathways. Different HBpin activations and mechanisms will be discussed in detail in section 3.

## 3 Hydroboration of Polarized Unsaturated Bonds

### 3.1 Aldehydes and Ketones

**Scheme 8:**
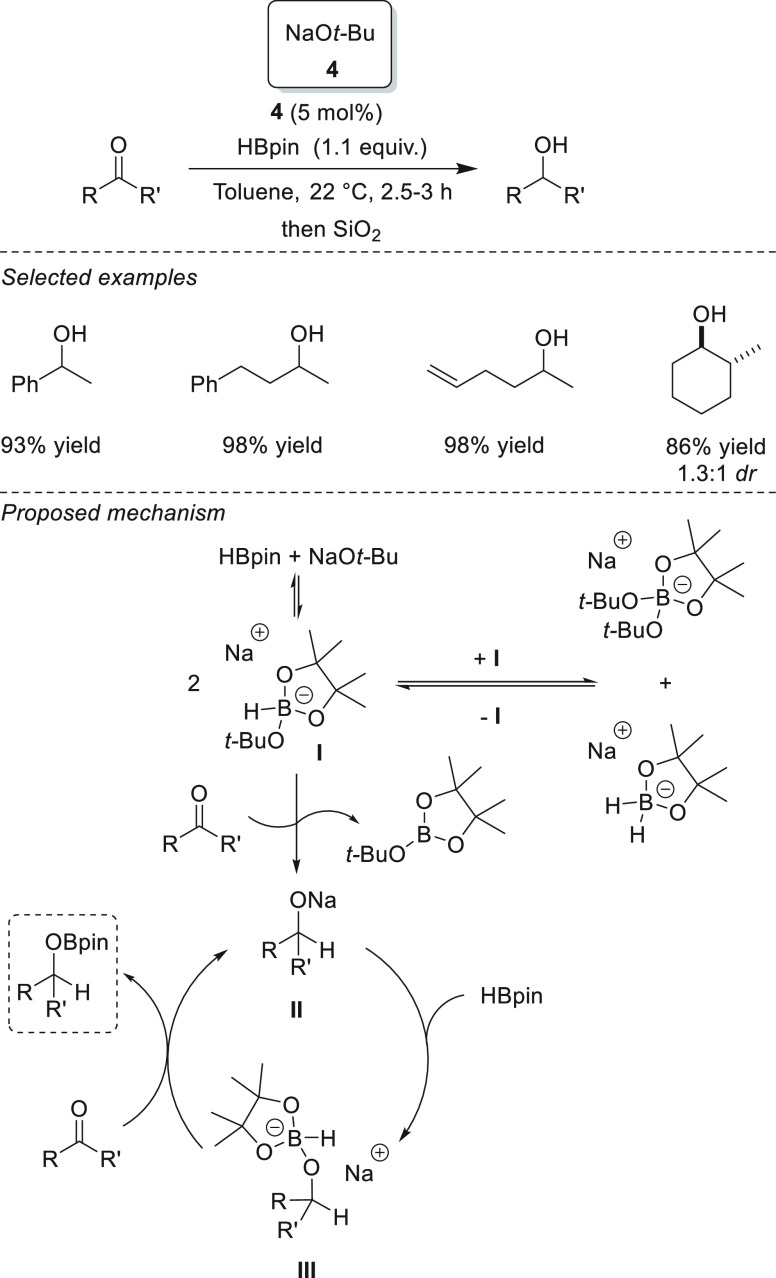
First Example of s-Block-Catalyzed Hydroboration of Ketones

The first example of s-block metal-catalyzed hydroboration of carbonyl compounds was reported in 2011 by Clark *et al.* and involved the use of sodium *tert*-butoxide **4** as the precatalyst (Scheme 8). The authors demonstrated that sodium alkoxide can catalytically activate pinacolborane toward the addition to C=O bonds in ketones. In this regard, the initial activation of pinacolborane by NaO*t*-Bu forms hydride species **I**, which adds to the C=O bond. The formed alkoxide **II** activates pinacolborane to generate species **III**, which subsequently adds to the C=O bond, generating the corresponding product and sodium alkoxide II, which enters the new catalytic cycle. Since the active hydride species could not be isolated or characterized by means of NMR spectroscopy, the authors postulated an equilibrium between sodium trialkoxyborohydride and other boron alkoxy and hydride species, which can also act as hydride sources.^73^

**Scheme 9:**
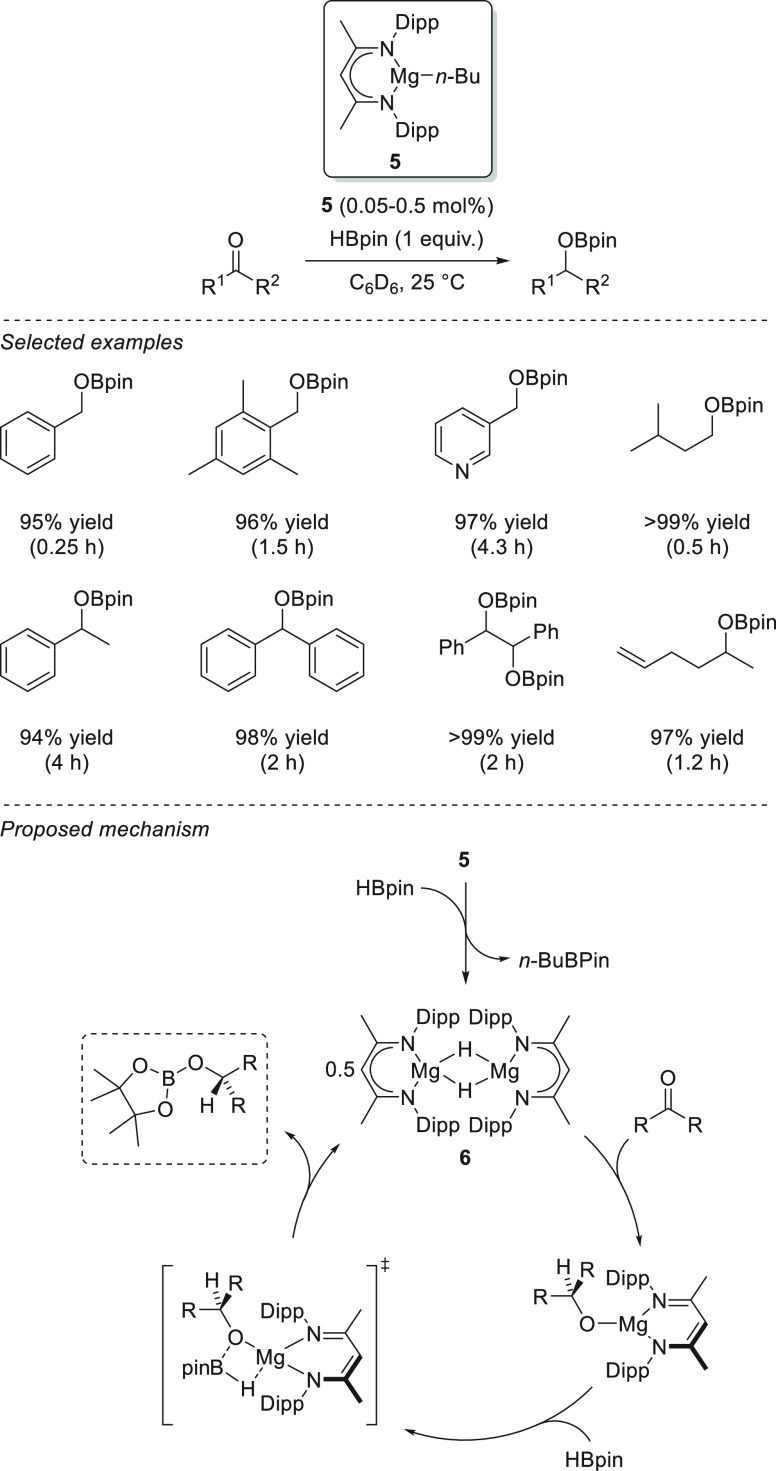
Magnesium-Catalyzed Hydroboration of Aldehydes and Ketones

β-Diketiminate magnesium complex **5**, as reported by Hill *et al.*, showed catalytic activity toward the hydroboration of aldehydes and ketones.^74^ Excellent yields were afforded for a wide range of carbonyl compounds for the first time using magnesium-based catalysts under mild reaction conditions (ambient temperature) and at low catalyst loadings (0.05–0.5 mol %). Mechanistically, the addition of HBpin to a solution of **5** leads, *via* σ-bond metathesis, to stoichiometric formation of *n*-BuBpin and heteroleptic magnesium hydride species **6**, which exists in equilibrium with labile magnesium borohydride species of the anion [*n*-BuHBpin]^−^. Next, the addition of the substrate leads to the formation of a heteroleptic magnesium species resulting from the insertion of the carbonyl group into the magnesium hydride bond. Finally, σ-bond metathesis with HBpin releases a boronic ester and recovers the catalyst (Scheme 9).

**Scheme 10:**
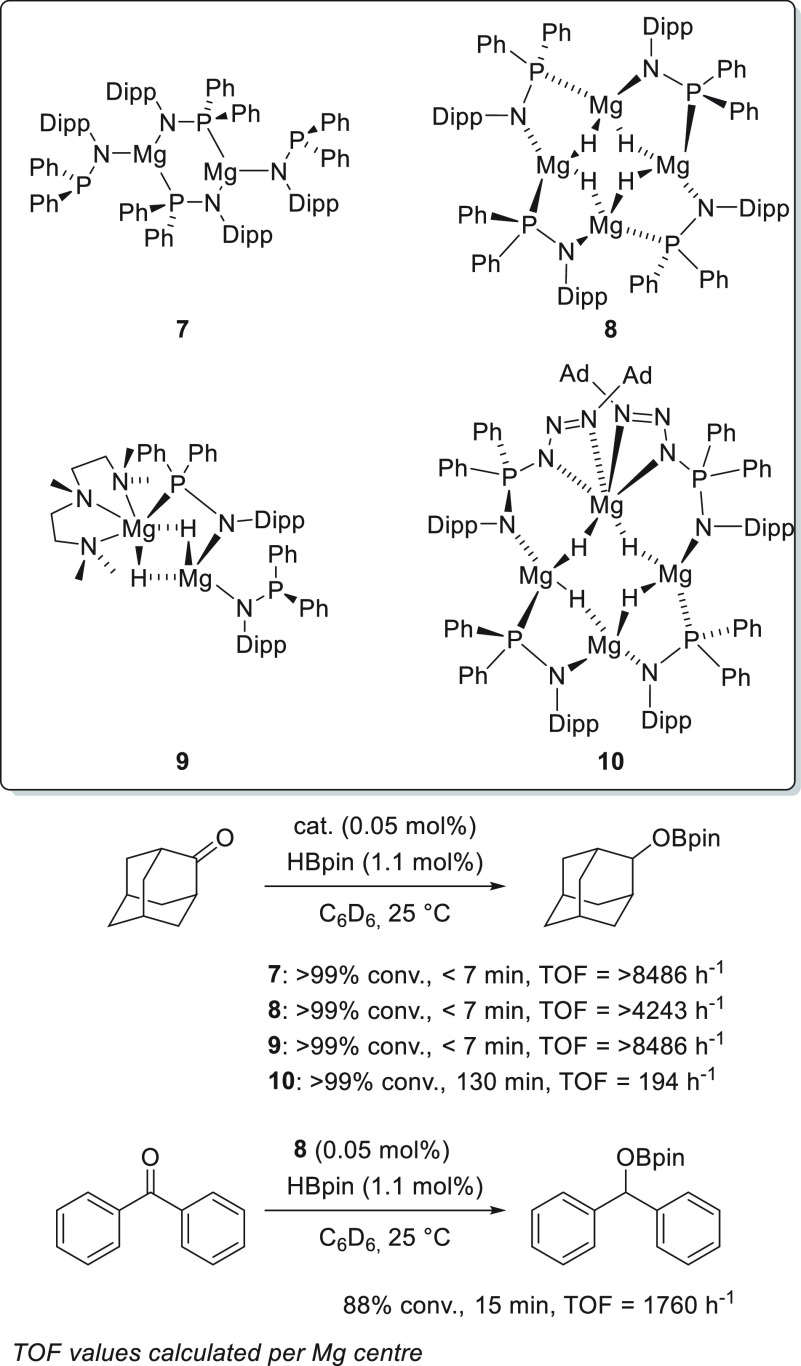
Ring-Shaped Phosphinoamido–Magnesium–Hydride Complexes for Hydroboration of Ketones

As described in section 2, Stasch *et al.* developed phosphinoamido–magnesium–hydride complexes **7**–**10** (Scheme 10) to investigate whether a ligand that favors bridging and terminal coordination modes can be beneficial in terms of activity compared to magnesium complexes such as **5**.^75^ Complexes **7**–**9** were shown to be very active for the hydroboration of ketones, providing quantitative conversions under mild reaction conditions, short reaction times, and low catalyst loadings (0.05 mol %). The authors, however, limited the substrate scope to only two ketones—benzophenone and 2-adamantanone.

The addition of Li–H species to the carbonyl group was first reported in 2012 by Stasch *et al.*, who showed that a hydrocarbon-soluble lithium hydride complex can effectively undergo hydrometalation to benzophenone.^51^

**Scheme 11:**
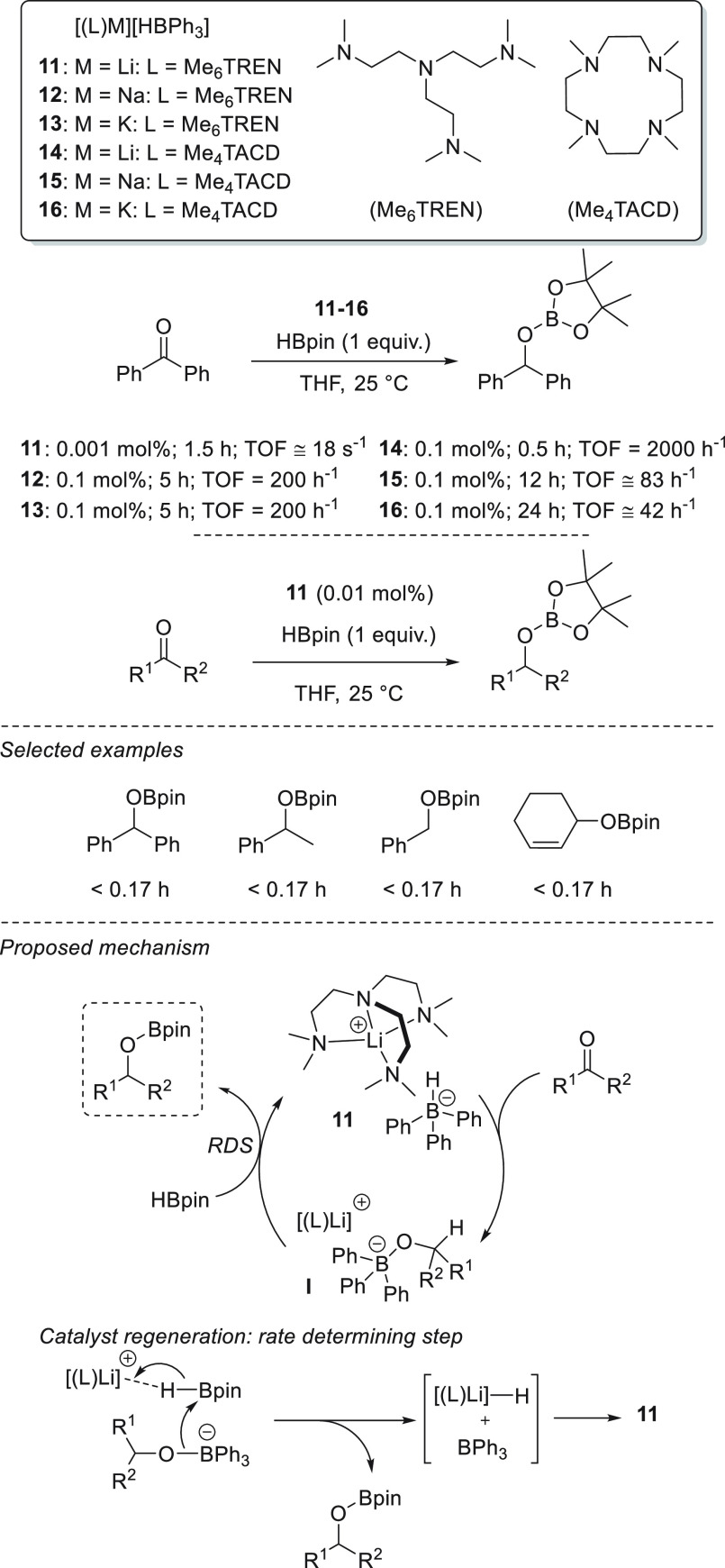
Alkali Metal Catalyzed Hydroboration of Carbonyl Compounds

It was not until 2016, however, that the first catalytic application of light alkali metal complexes was reported. Okuda *et al.* employed a series of lithium, sodium, and potassium hydridotriphenylborate complexes **11**–**13** for the selective hydroboration of benzophenone as a model substrate (Scheme 11).^35^ Compared to sodium and potassium complexes **12** and **13**, respectively, lithium complex **11** exhibited superb activity, exhibiting a remarkably high TOF of 66.6 × 10^3^ h^–1^ or 18 s^–1^.

Complex **11**, the most active catalyst, was applied for the hydroboration of several ketones and aldehydes. Mechanistically, the authors postulate that lithium hydridotriphenylborate **11** reacts rapidly with the carbonyl compound to give intermediate [(L)M][R^1^R^2^CHOBPh_3_] (**I**), whereas no reaction between **11** and HBpin or BPh_3_ and HBpin was observed. Finally, intermediate **I** reacts further with HBpin to give the desired product and regenerated **11**. The insertion step appears to be equally fast for all metals (Li, Na, and K), but the catalyst regeneration (or group transfer) is faster for Li complex **11** than for Na and K **12** and **13**, respectively. The authors suggested that the group-transfer step is rate determining. The group-transfer step through a direct hydride–alkoxide exchange *via* σ-bond metathesis was discarded, and regeneration of the active species **11** was suggested to occur by hydride abstraction from HBpin to generate LiH and BiPh_3_ (Scheme 11). Moreover, coordination of Me_6_TREN is crucial for high activity; in the absence of the coordinating ligand, the catalyst activity significantly decreased. The Me_6_TREN ligand offers a unique combination of flexible coordination and retention of the Lewis acidity of the lithium cation to become a highly active catalyst. Thus, the high activity of the Li catalyst is thus explained by the higher degree of polarization of lithium in the [(L)Li][R^1^R^2^CHOBPh_3_] intermediate compared with sodium and potassium. Moreover, this chelation most likely prevents lithium from forming aggregates.^36^ Similarly, macrocyclic Me_4_TACD has also been used as an *N,N,N,N*-type neutral ligand to afford complexes **14**–**16**.^37^ When applied for hydroboration of benzophenone, these complexes exhibited much lower activity than their Me_6_TREN analogues. Nevertheless, the reactivity trend of Li ≫ Na ≥ K was also observed in this case.

**Scheme 12:**
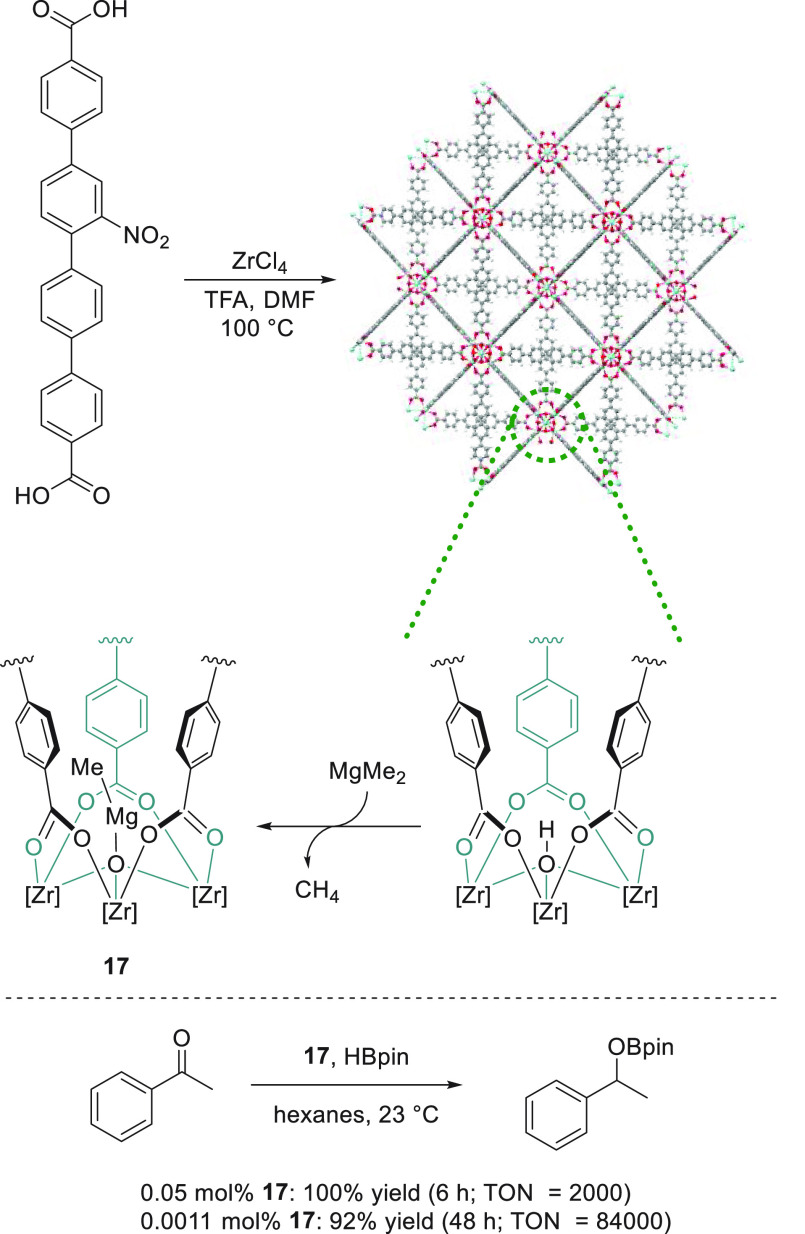
Magnesium Stabilization by a TPHN-MOF Developed by Lin **et al.**

Lin *et al.* reported a simple strategy to stabilize heteroleptic magnesium alkyl species by a TPHN-metal–organic framework (TPHN = 4,4′-bis(carboxyphenyl)-2-nitro-1,1′-biphenyl), thus avoiding any kind of Schlenk equilibrium that could lead to inactive Mg species. The authors succeeded in a straightforward metalation of secondary building units of Zr-MOF with MgMe_2_ and the application of this magnesium-supported catalyst **17** (Scheme 12) for the hydroboration of a wide range of carbonyl compounds, such as aldehydes and ketones. Impressively, Mg-functionalized MOF **17** displayed high turnover numbers and could be reused more than 10 times with no loss of activity.^76^

**Scheme 13:**
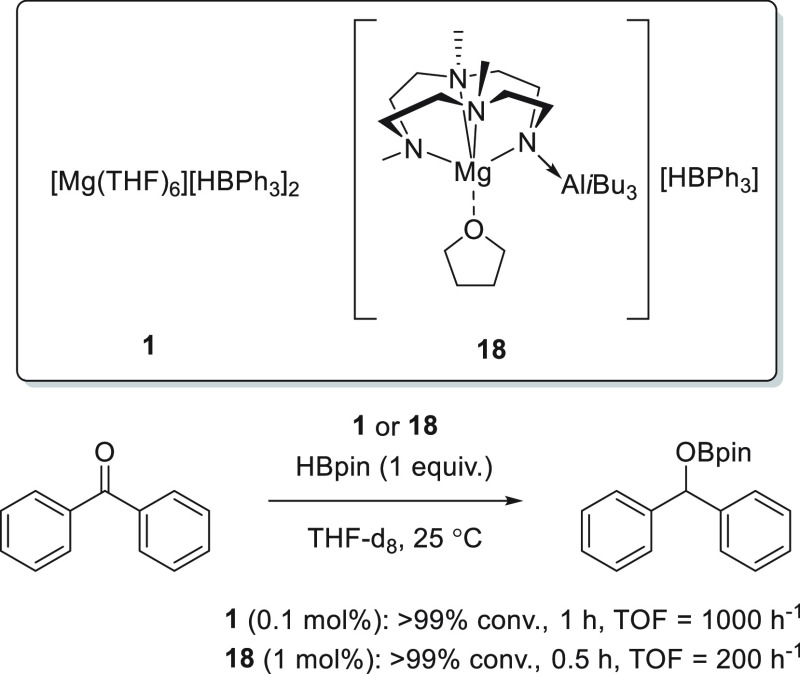
Magnesium Complexes for the Hydroboration of Ketones Developed by Okuda *et al.*

In addition, Okuda *et al.* developed magnesium hydrotriphenylborate complex **1** (Scheme 13), which proved to be an active catalyst for the hydroboration of various polarized bonds.^45^ Although complex **1** was active for the hydroboration of aldehydes with as low as 0.05 mol % catalyst loading, lower conversions and longer reaction times were required when ketones were used in the reaction. Moreover, since the reactions were carried out in DMSO, the authors observed competition between the reduction of carbonyls and sulfoxides (see section 3.10).

To expand the catalyst versatility, Okuda *et al.* designed a new molecular magnesium complex **18** containing an *N,N,N,N*-type macrocyclic ligand (Scheme 13). Interestingly, the basic amido function is blocked by the Al(*i*Bu)_3_ coordination in order to avoid the formation of large clusters.^77^ Complex **18** was subsequently applied for the catalytic hydroboration of a wide range of substrates, including ketones. Compared to its ligand-free analogue **1**, magnesium complex **18** showed lower catalytic activity toward the hydroboration of ketones which may be explained by the oversaturated, sterically encumbered five-coordinate magnesium center.

**Scheme 14:**
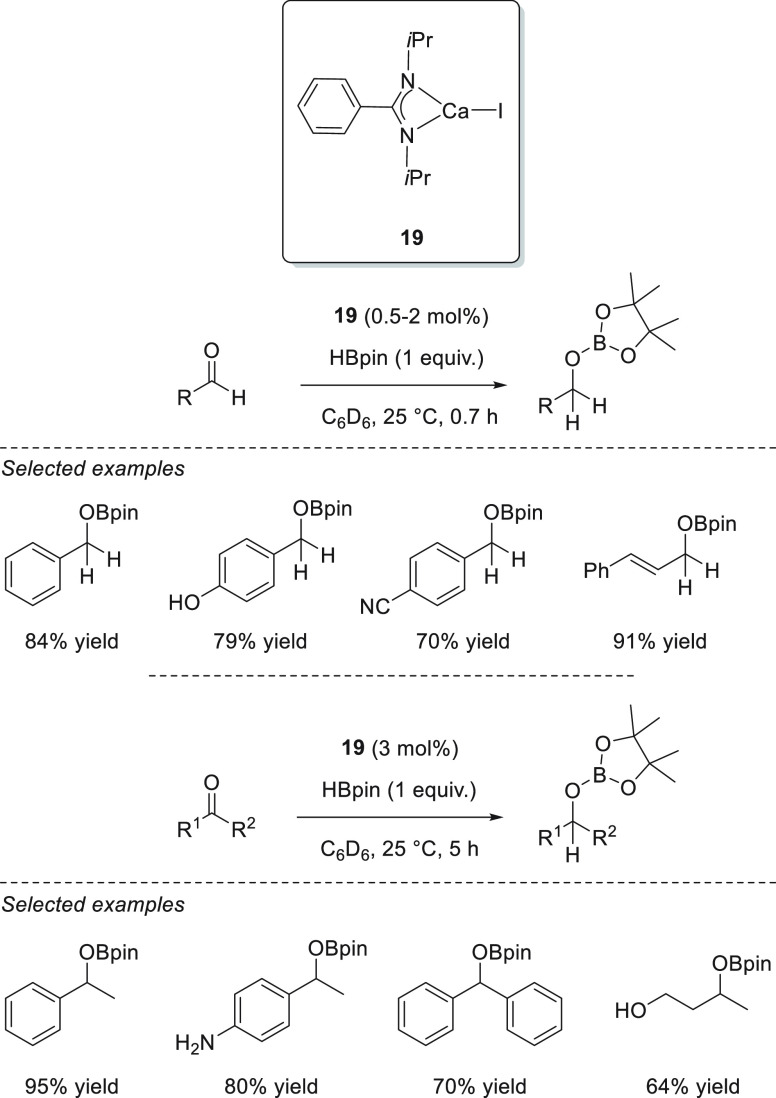
Calcium-Catalyzed Hydroboration of Carbonyl Compounds

It was not until 2017 that the first calcium-catalyzed hydroboration of carbonyl compounds appeared. Sen *et al.* designed a benzamidinato calcium complex **19** that was active toward the hydroboration of aldehydes and ketones (Scheme 14). By using complex **19**, excellent yields could be obtained under mild reaction conditions, short reaction times, and low catalyst loadings.^78^ Moreover, this catalytic system showed good functional group tolerance toward OH and NH groups as well as C–C double bonds.

**Scheme 15:**
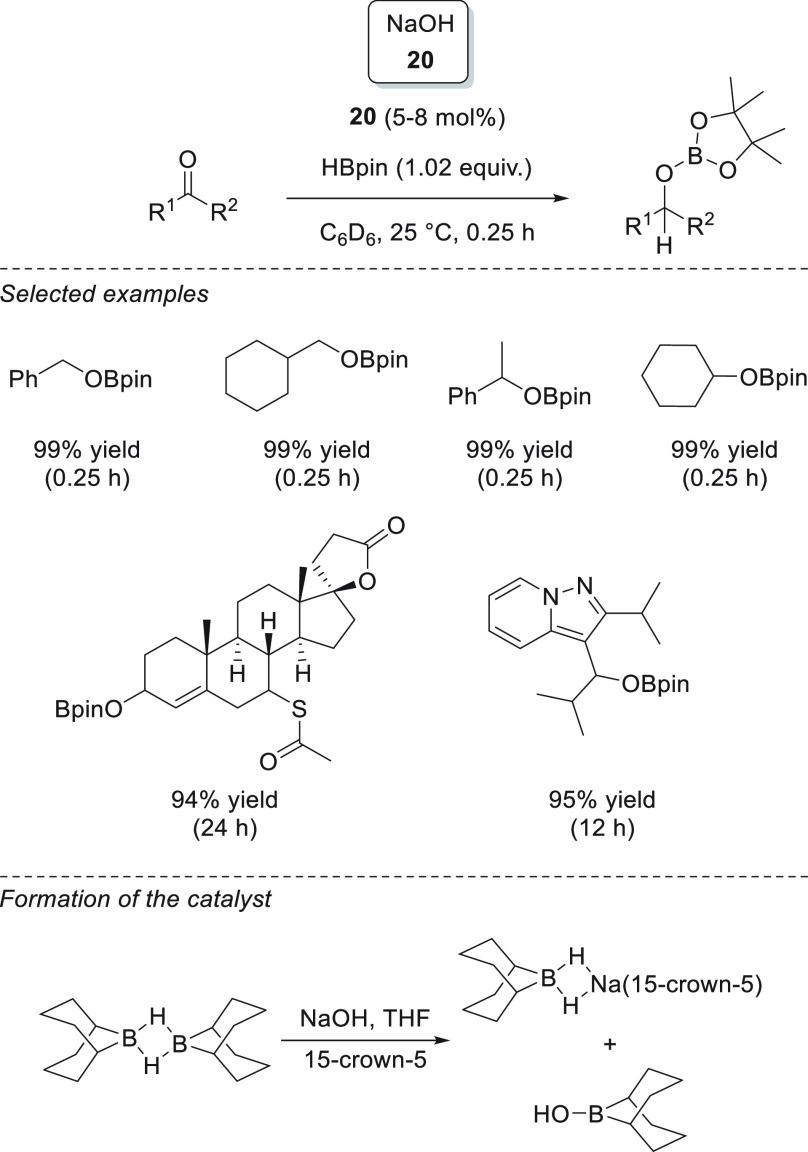
Na-Catalyzed Hydroboration of Carbonyl Compounds

Wu, Liu, and Zhao *et al.* showed that a catalytic hydroboration of carbonyl compounds could be initiated simply by NaOH **20** (Scheme 15).^79^ The authors postulated that the reaction is possible due to the formation of an anionic borodihydride species from the reaction of borane and NaOH, which then acts as a precatalyst. The formation of the anionic borodihydride was corroborated when the authors used 9-BBN as a hydride source to isolate and characterize the dihydride species, as the attempt to obtain such dihydride from HBpin resulted in the formation of unanalyzable species.

**Scheme 16:**
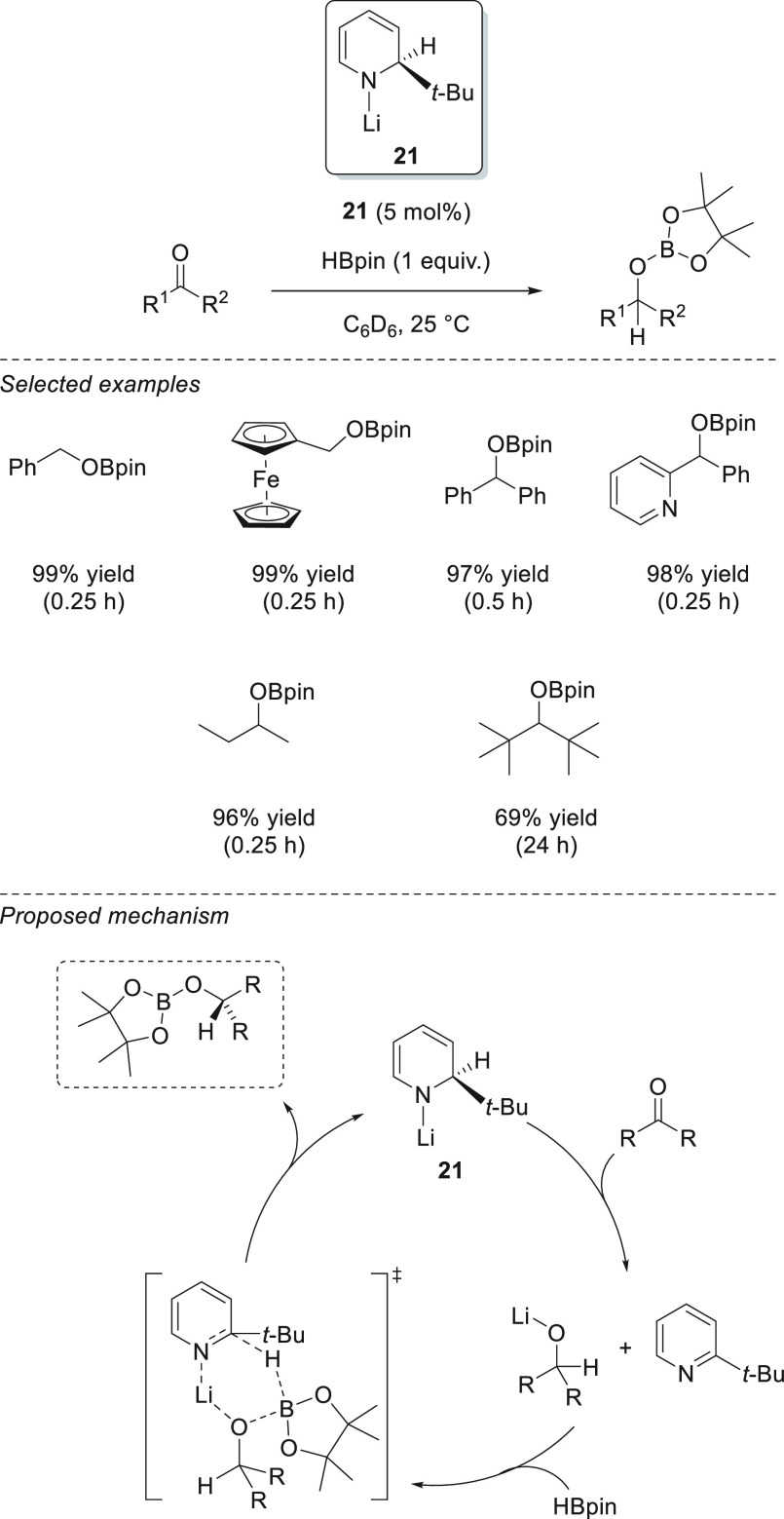
Li-1,2-Dihydropyridine-Catalyzed Hydroboration of C=O Bonds

Taking advantage of the excellent hydrogen-transfer ability of dihydropyridines, Mulvey and Roberston *et al.* described the successful application of lithium *tert*-butyldihydropyridine complex **21** in the hydroboration of aldehydes and ketones.^80^ This highly hydrocarbon-soluble catalyst exhibited excellent activities, providing quantitative conversions in less than 30 min, in almost all cases. On the basis of the NMR studies, the authors suggested that complex **21** undergoes hydrometalation of the C=O bond, releasing rearomatized *t*-BuPy and Li alkoxide. Finally, the Li alkoxide species activates HBpin *via* a 6-membered transition state with *t*-BuPy to regenerate the Li-1,2-dihydropyridine **21** and the desired boronic ester (Scheme 16).

**Figure 4:**
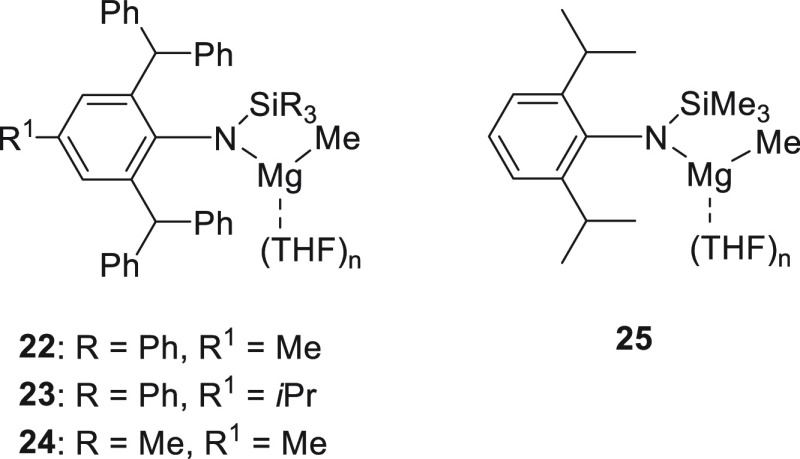
Various magnesium-based precatalysts for the hydroboration of carbonyl compounds developed by Ma *et al.*

Ma *et al.* designed a series of bulky amido magnesium complexes **22**–**25** (Figure 4), which could be easily prepared by treating the corresponding secondary amine and 2 equiv of the Grignard reagent (MeMgI).^81^ All complexes exhibited excellent activities toward ketone hydroboration, although complex **25** provided the best results. However, when sterically hindered ketones were used, elevated temperature and prolonged time were necessary to achieve good conversion.

**Scheme 17:**
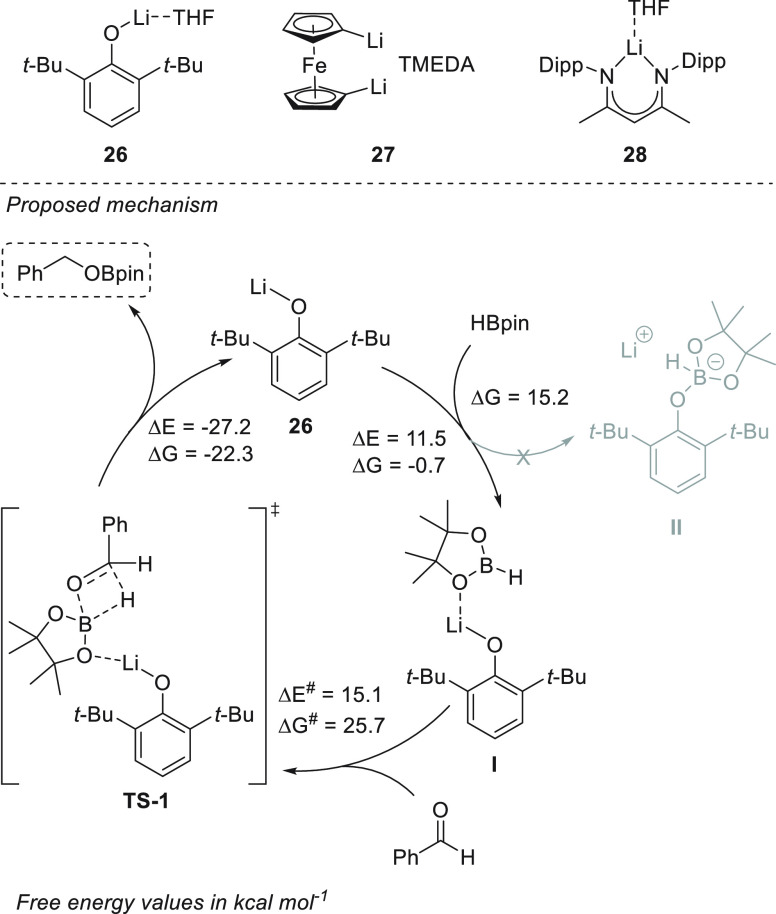
Mechanism of Li-Based Hydroboration of Carbonyl Compounds in the Presence of Catalysts **26** and **27** developed by Sen *et al.*

In 2018, Sen *et al.* reported a wide range of accessible and active lithium compounds (**26**–**28**, Scheme 17).^82^ 2,6-Di-*tert*-butyl phenolate lithium **26**, 1,1′-dilithioferrocene **27**, and β-diketiminate lithium **28** and exhibited excellent activities toward aldehyde and ketone hydroboration. Based on the results of NMR spectroscopy and DFT calculations, the authors provided mechanistic insight into the reaction catalyzed by complex **26**. Coordination of the oxygen atom of HBpin to lithium complex **21** forms Li-HBpin adduct **I**. The concerted attack of the boron center by the *O*-atom of the carbonyl group and of the electrophilic carbon by the hydride group leads to the formation of a four-membered transition state (**TS-1**, Scheme 17) and eventually to the formation of a boronic ester and the regeneration of the catalyst **26**. The possibility of a phenolate attack to the pinacolborare to form the boronate complex **II** was ruled out based on a thermodynamically unfavored pathway (Δ*G* = −0.7 kcal mol^–1^ for **I** and Δ*G* = 15.2 kcal mol^–1^ for **II**).

**Scheme 18:**
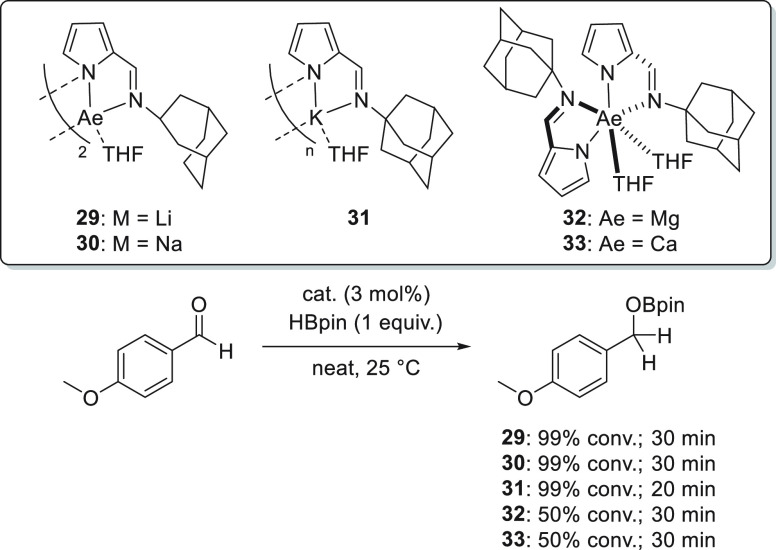
*N*-Adamantyl–Iminopyrrolyl Complexes for the Hydroboration of Carbonyl Compounds

A comparative study of the hydroboration of *p*-methoxybenzaldehyde using *N*-adamantyliminopyrrolyl complexes **29**–**33** with Group 1 and Group 2 metals was carried out by Panda *et al.* (Scheme 18).^41^ The results showed that group 1 complexes rapidly led to the formation of the corresponding product within 30 min for lithium and sodium complexes **29** and **30**, respectively. The potassium analogue showed the highest activity, and the reaction was finished in less than 20 min. Thus, potassium complex **31** was applied for the hydroboration of aldehydes and ketones, showing good functional group tolerance. Moreover, the authors found that magnesium complex **32** and calcium complex **33** exhibited similar reactivity and were less active than the corresponding group 1 metal complexes **29**–**31**.

**Scheme 19:**
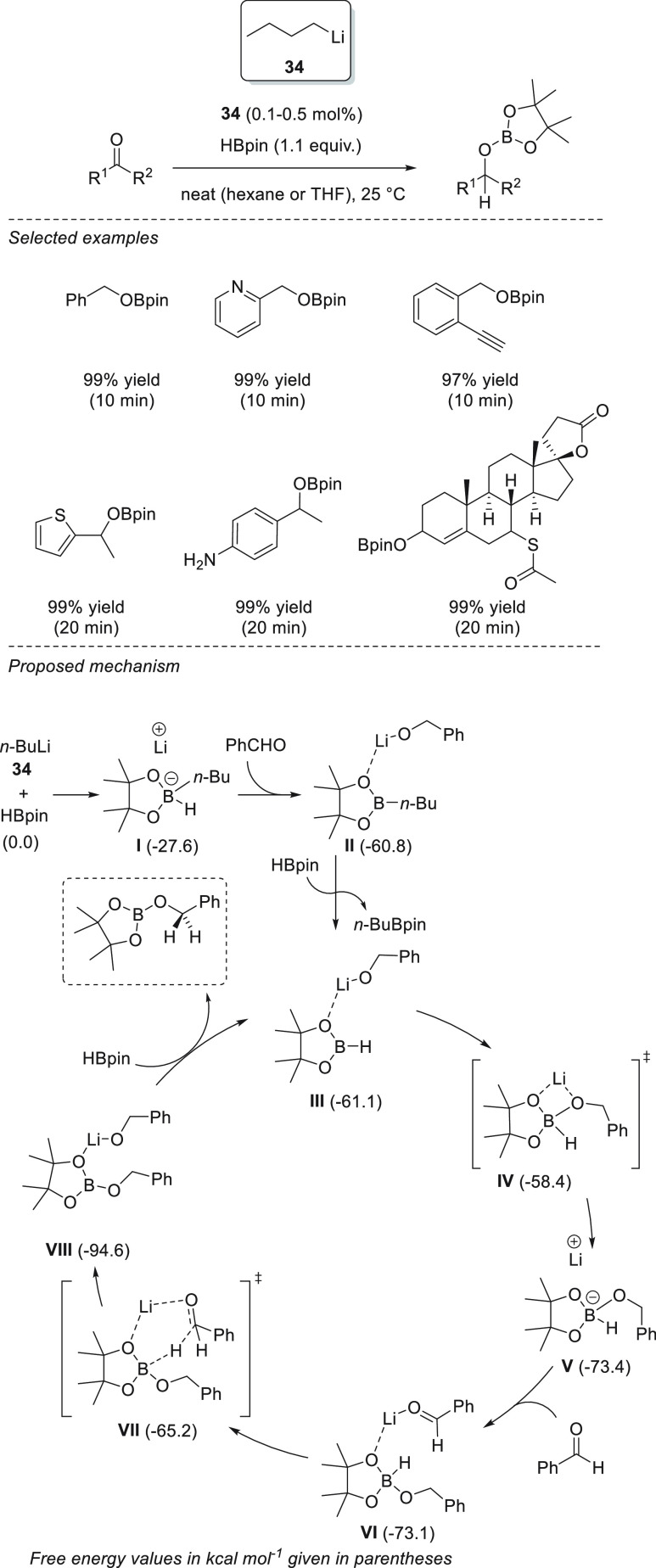
Mechanism of the Li-Catalyzed Hydroboration of C=O Bonds

Xue and Bao *et al.* demonstrated that readily available *n*-BuLi **34** is able to effectively catalyze the hydroboration of aldehydes and ketones. With as little as 0.1 mol % precatalyst, the authors were able to reduce aromatic and aliphatic aldehydes and ketones, showing excellent functional group tolerance, under mild reaction conditions: in most cases in just 20 min. On the basis of DFT calculations, the authors proposed that *n*-BuLi **34** reacts with pinacolborane to provide a Li-butylborate species **I** which upon reduction of the aldehyde leads to the lithium alkoxide, which presumably binds to the *n*-BuBpin, forming adduct **II**. Adduct **II** then undergoes ligand exchange with pinacolborane, affording species **III** to form the active catalytic species. A thermodynamically favored nucleophilic attack of the alkoxide to the boron atom affords Li boronate species **V**, in which a carbonyl compound binds to the lithium cation, favoring the hydride attack (**TS VII**). The obtained species **VIII** finally reacts with another molecule of pinacolborane, affording the desired compound and the regenerated active species **III** (Scheme 19).^83^

A similar study was reported at the same time by An *et al.*, who showed the excellent reactivity of *n*-BuLi **34** toward the hydroboration of aldehydes and ketones. Varying the reaction conditions compared to those used by Xue and Bao *et al.*,^83^ such as performing reactions with higher catalyst loading but lower temperature (0 °C) and using THF as a solvent, resulted in formation of the desired product in just 5 min. Remarkably, α,β-unsaturated ketones and aldehydes underwent selective 1,2-hydroboration, affording the corresponding allylic alcohols.^84^

**Scheme 20:**
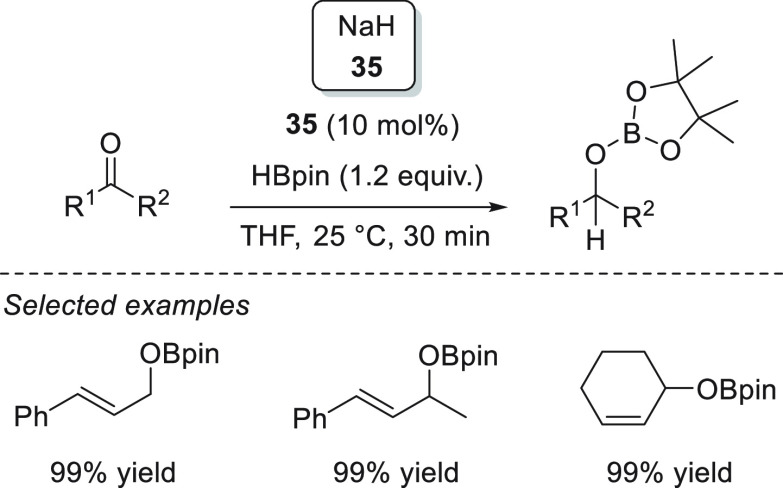
NaH-Catalyzed Hydroboration of Ketones and Aldehydes

Moreover, the same authors also reported the catalytic application of NaH **35** for the hydroboration of aldehydes and ketones (Scheme 20).^85^ This NaH-catalyzed hydroboration of α,β-unsaturated substrates was completely regioselective, affording the corresponding allylic alcohols in excellent yields.

**Scheme 21:**
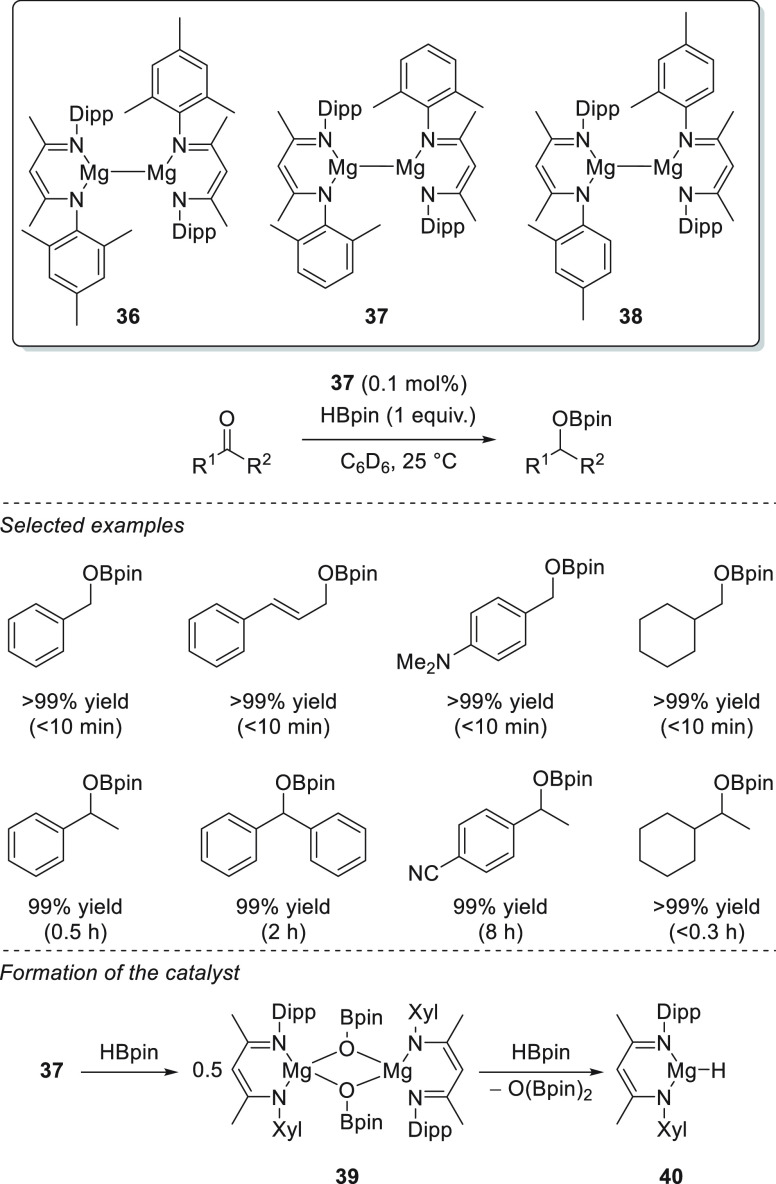
Mg(I) Dimers Developed by Ma **et al.** and Their Activation toward Active Mg–H Species

The concept of magnesium(I) complexes was first presented in 2007 by Stasch *et al.*;^86,87^ however, their first application in catalytic hydroboration was reported by Ma *et al.*, who applied a series of unsymmetrical β-diketiminatomagnesium(I) complexes **36**–**38** as precatalysts (Scheme 21) for the hydroboration of aldehydes and ketones, among others.^88^ The reduction of C=O bonds was performed under mild reaction conditions and at low catalyst loadings. From a mechanistic point of view, the authors proposed that dimeric Mg(I) complex **38** reacts with HBpin to form dimeric magnesium boryloxide complex **39**, arising from the decomposition of HBpin. Compound **39** reacts with another molecule of HBpin, forming catalytically active Mg(II) complex **40**. Although catalytically active Mg(II) **40** has been reported as a well-defined species active toward hydroboration of unsaturated bonds, it is worth mentioning that the combination of Mg(I) dimers with pinacolborane provides additional reactive boron-containing species, which could also be considered active in hydroboration (see section 7.1).

**Scheme 22:**
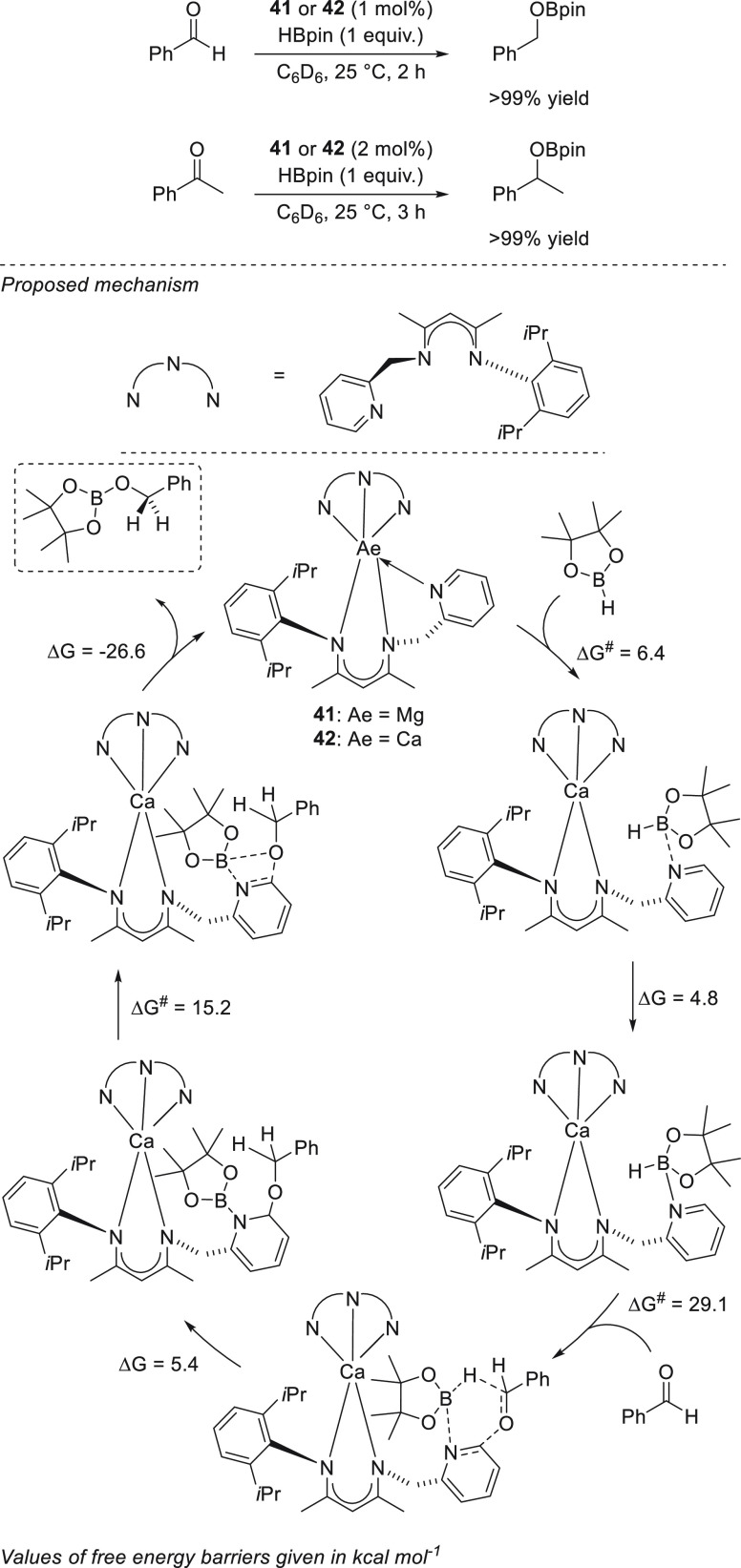
Mechanism Catalyzed by Methylpyridine β-Diketiminato Magnesium and Calcium Catalysts

An interesting use of magnesium and calcium complexes for the hydroboration of aldehydes and ketones has been reported by Vanka and Sen *et al.* Here, β-diketiminatomagnesium and -calcium complexes **41** and **42** allowed formation of the desired products under mild reaction conditions, short reaction times, and low catalyst loadings (Scheme 22). On the basis of the hemilabile bond between the pyridyl group and the metal center as well as the results of DFT calculations, the authors postulated that the metallic center does not partake in any activation, but rather binds two ligands, each of which is capable of acting as a catalytic site for the hydroboration. Thus, the calcium enables the formation of a dual site catalyst, which would be more efficient than just employing a pyridine moiety as a single site catalyst in the reaction. It should be noted, however, that the authors did not conduct any control experiments with just a ligand or pyridine to confirm the proposal.^89^ The presented work could therefore be considered as an example of organocatalytic transformation.

Following the trend of Xue and Bao, who used readily available *n*-BuLi as a precatalyst,^83^ Kuciński and Hreczycho reported that commercially available and inexpensive LiHBEt_3_**43** shows high activity toward the catalytic hydroboration of a wide range of aldehydes and ketones.^90^ Under solvent-free conditions, with as little as 0.1 mol % precatalyst, quantitative conversions were reported for a wide range of substrates.

Furthermore, An *et al.* reported the successful hydroboration of aldehydes and ketones using lithium *tert*-butoxide **44**(91) and potassium carbonate **45**(92) as precatalysts. Excellent yields were obtained under mild reaction conditions and catalyst loadings of 0.5–1 mol %. Interestingly, lithium *tert*-butoxide **44** showed higher activity than its sodium analogue **4**, which was studied by Clark *et al.*(73)

**Scheme 23:**
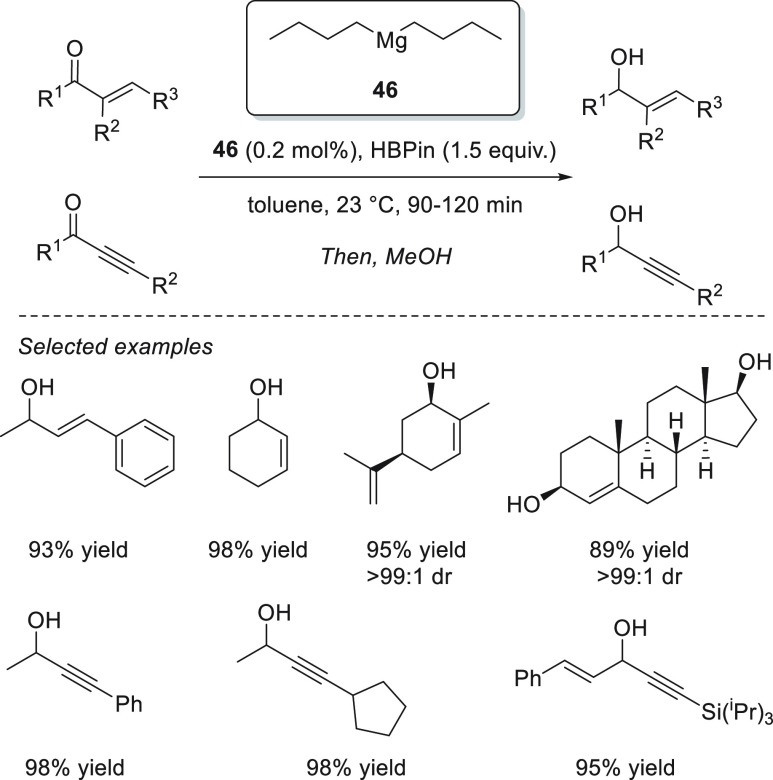
Mg-Catalyzed Regioselective Hydroboration of α,β-Unsaturated Ketones, Developed by Rueping *et al.*

Although commercially available lithium complexes were tested as efficient precatalysts, commercially available magnesium compounds were not applied for the hydroboration of carbonyl compounds until Rueping *et al.* applied readily available Mg(*n*-Bu)_2_**46** for the chemoselective hydroboration of α,β-unsaturated ketones (Scheme 23). For the first time, this simple and readily available precatalyst was successfully applied for the highly 1,2-selective hydroboration of α,β-unsaturated ketones, achieving excellent yields and chemoselectivities for a wide range of enones and ynones with low catalyst loadings, short times, and mild reaction conditions.^93^ Compared to other Mg complexes previously applied for hydroboration of α,β–unsaturated compounds,^45,76^ precatalyst **46** provides better conversions in shorter times.

Moreover, Grignard reagents have been applied by Ma *et al.* in the hydroboration of aldehydes and ketones. The high concentration of the reaction mixture ensured excellent conversions in the presence of MeMgI **47** (aldehydes: 0.05 mol %, ketones: 0.5 mol %) in 20 min.^94^

**Scheme 24:**
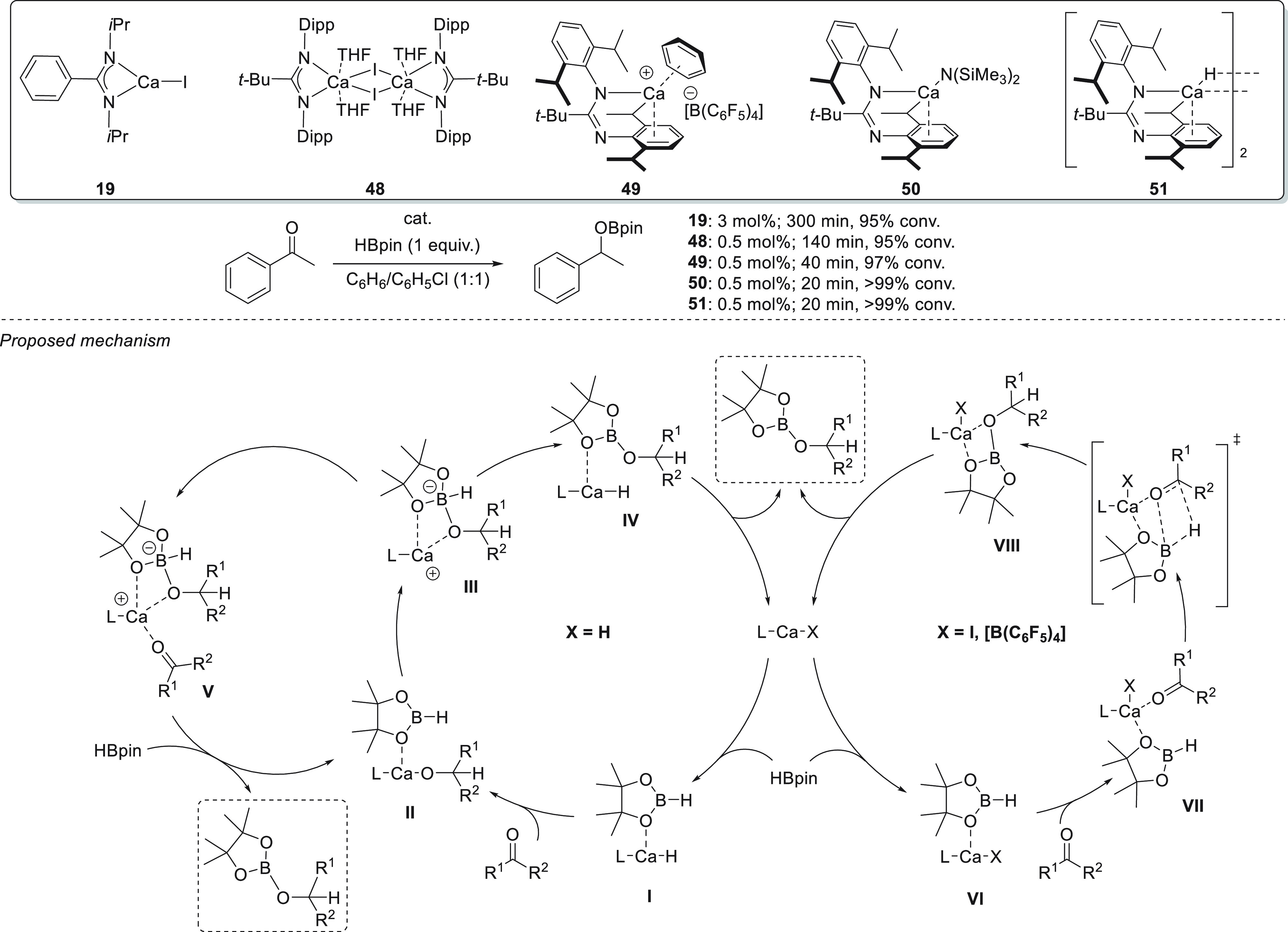
Calcium Catalysts for Hydroboration of Ketones

Very recently, Harder *et al.* reported the use of calcium amidinate complexes **48**–**51** with various anionic ligands or counterions for the hydroboration of ketones^95^ and compared their activity with that of previously reported complex **19** (Scheme 24).^78^ The authors concluded that complex **48** exhibited far better activity than complex **19**. The authors attribute this observation to the different amidinate spectator ligands’ bulkiness (buried volume *V*_B_ = 34.0% for **48** and *V*_B_ = 28.1% for **19**), both in *N*,*N*-coordination mode, and the presence of aryl substituents in complex **48**, making calcium more electrophilic. Further increasing the metal center electrophilicity by introducing the [B(C_6_F_5_)_4_]^−^ anion (complex **49**) led to an improvement in the catalytic performance. Finally, catalysts **50** and **51** outperformed all the other catalysts, which led to the proposal of two different mechanistic pathways depending on the ligand.

Mechanistically, for hydridic complexes **50** and **51** the authors proposed that in similar metal–hydride hydroboration reactions, Ca–H **I** undergoes hydrometalation to provide calcium alkoxide **II**, which *via* nucleophilic attack to the boron atom forms zwitterionic species **III**. Finally, after hydride transfer, complex **IV** is obtained, the product is released, and the catalyst regenerates the catalysts. Alternatively, a pathway in which hydride is not transferred from the metal to the ketone, but directly from the borate (species **V**), cannot be excluded.

In the case of catalysts **48** and **49**, no metal hydride formation was observed, as activation of HBpin occurs *via* Ca–O interactions. This behavior is in agreement with the increasing reaction rates and Lewis acidity of the calcium center. Thus, the authors postulated a mechanism for **48** and **49** in which the metal catalyst activates HBpin to form adduct **VI**. Substrate coordination ensures polarization of the C=O bond (**VII**), thus making it reactive toward hydride addition *via* direct B–H/C=O addition. Lastly, species **VIII** releases the corresponding boronic ester product and the catalyst is regenerated.

Kuciński and Hreczycho reported the first example of a catalytic hydroboration in the presence of easily available potassium fluoride (KF) **52**. Thus, ketones and aldehydes, bearing a wide range of functional groups, were successfully reduced to the corresponding primary and secondary alcohols under mild reaction conditions (DMF, room temperature) and short reaction times (30–60 min).^96^

Similarly, An *et al.* applied lithium bromide (LiBr) **53** for hydroboration of various organic compounds, such as aldehydes and ketones, acid chlorides, esters, amides, nitriles, alkenes, and alkynes as well as epoxides. Out of all tested substrates, only hydroboration of aldehydes and ketones provided the desired products. With as little as 0.5–1 mol % of LiBr, various substrates bearing aliphatic and aromatic substituents were reduced under mild reaction conditions and in short reaction times.^97^

**Scheme 25:**
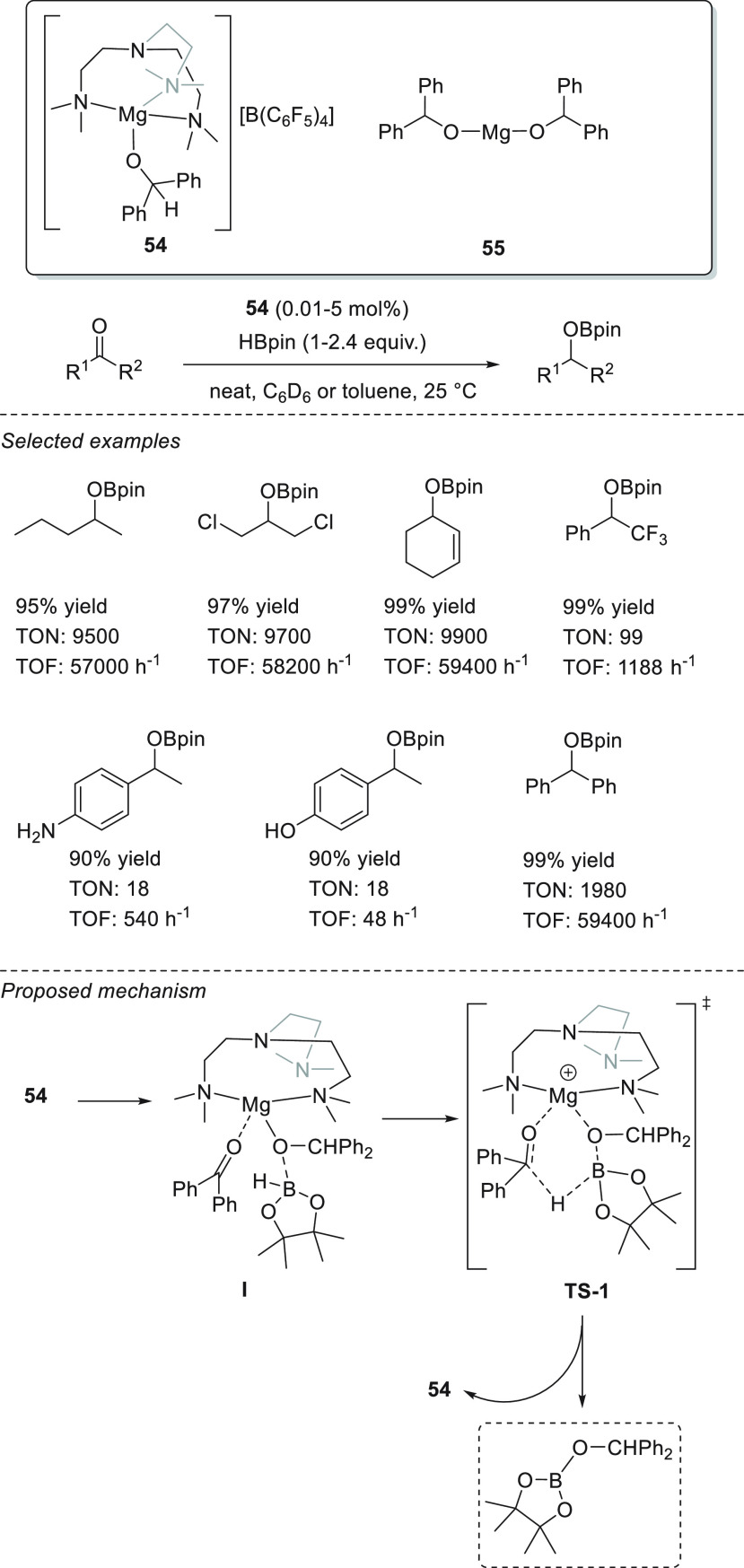
Magnesium Alkoxide 55 Catalyzed Hydroboration of Ketones

Very recently, Maron, Venugopal, and co-workers reported (Me_6_TREN)–magnesium alkoxide complex **54** and magnesium dialkoxide **55** as active catalysts for the hydroboration of ketones.^98^ The sterically hindered magnesium complex **54** was employed as a model catalyst to explore the role of magnesium alkoxides for ketone hydroboration (Scheme 25). Control experiments and DFT calculations suggest that the hydride transfer from pinacolborane to the C=O bond occurs in a concerted reaction pathway through a six-membered ring transition state (**TS-1**). On the basis of these results, the authors discarded the possibility of the formation of any magnesium hydride intermediate (cf. Scheme 28). The authors explored the substrate scope by using homoleptic dialkoxide **55** as a simplified version of **54**. Excellent yields were obtained for a wide range of dialkyl ketones, enones, and acetophenone derivatives, showing good functional group tolerance. It is important to highlight the excellent activities of **55**, competing favorably with the other Group 1 and Group 2 metal catalysts.

**Scheme 26:**
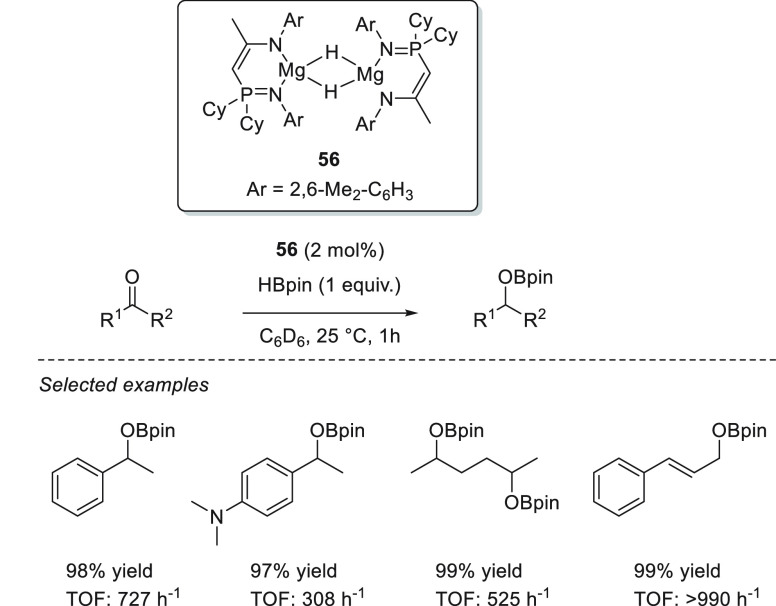
Dinuclear Magnesium Hydride Catalyzed Hydroboration of Ketones

Recently, Liu and Cui reported the activity of dinuclear magnesium hydride **56** stabilized by a phosphinimino amide ligand.^99^ Under mild reaction conditions and in short reaction times, complex **56** was able to successfully catalyze the hydroboration of a wide range of aldehydes, acetophenone-derived ketones, and enones (Scheme 26).

#### Asymmetric Hydroboration of Ketones

Although the catalytic hydroboration of carbonyl compounds has been widely studied using magnesium catalysts, the catalytic enantioselective version has been exclusively limited to transition-metal catalysts.^100−103^ Even though there are several examples of enantioselective magnesium catalysis reported thus far,^104,105^ there are only two examples of enantioselective magnesium-catalyzed hydroborations.^106^

**Scheme 27:**
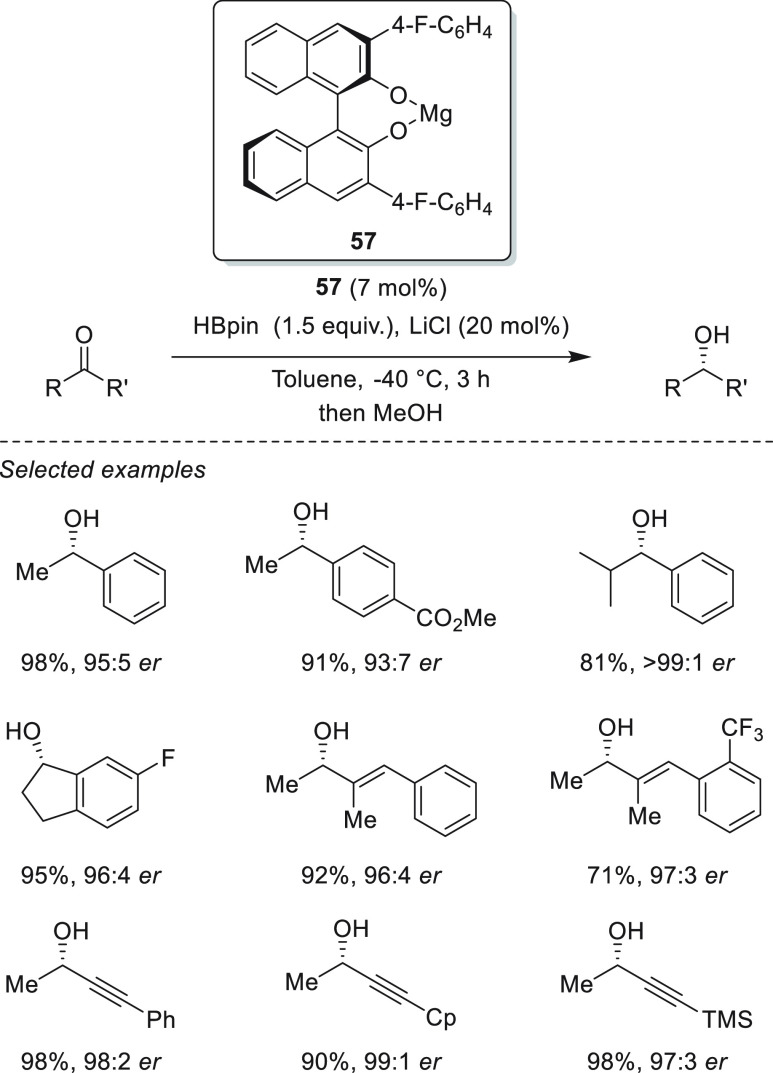
First Example of Enantioselective Magnesium-Catalyzed Hydroboration of Prochiral Ketones Developed by Rueping *et al.*

**Scheme 28:**
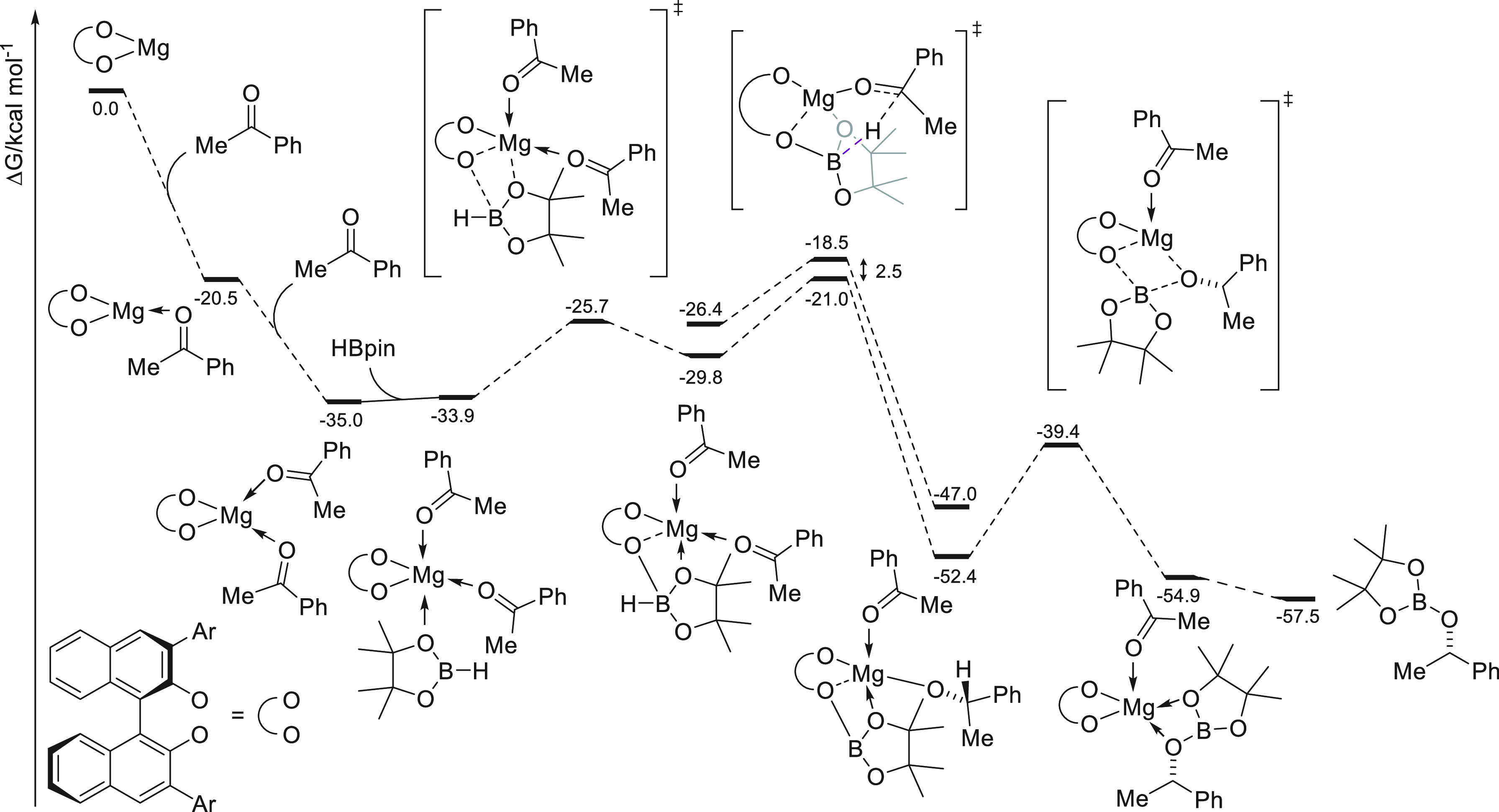
Mechanism of Enantioselective Magnesium-Catalyzed Hydroboration of Prochiral Ketones Developed by Rueping *et al.*

The first example of enantioselective magnesium hydroboration of prochiral ketones was reported by Rueping *et al.* using Mg-(*R*)-BINOL derived complex **57**.^107^ With this *in situ* formed catalyst,^108^ excellent yields and enantioselectivities could be obtained for a wide range of acetophenone and 1-indanone derivatives (Scheme 27). Moreover, catalyst **57** was applied for the hydroboration of α,β-unsaturated ketones, with exclusive 1,2-addition, achieving excellent enantioselectivities for a wide range of enones and ynones. The authors suggested a cooperative magnesium–ligand activation mode of HBpin, which is supported by the results of NMR spectroscopy and DFT calculations (Scheme 28). NMR experiments showed that, in contrast to other magnesium hydroboration examples, no Mg–H species was observed when **57** and HBpin were mixed. NMR spectroscopy measurements also showed that in stoichiometric experiments only one molecule of HBpin is necessary for the quantitative reduction of ketone, in agreement with the fact that no Mg–H formation is required for the reaction. In this regard, DFT calculations disclosed an alternative pathway. After substrate coordination to **57**, HBpin coordination takes place *via* a dual coordination with the Mg center (*via* O atom from HBpin) and with the O atom of BINOL ligand (*via* B atom from HBpin). Through coordination the HBpin moiety becomes more electron-rich, which facilitates hydride transfer to the previously coordinated C=O bond. DFT calculations also indicate that the BINOL derivative can act as a noninnocent ligand and is involved in HBpin activation. In this regard, when HBPin coordinates to the O atom of **57**, it becomes more electron rich and facilitates hydride transfer to C=O bond. This hydride transfer is also favored not only by the increase of the hydridic character of HBpin but presumably also by the increased electrophilic character of the substrate upon coordination to the Mg center. The computed energy profile reveals that the hydride-transfer step is predicted to be the rate-limiting and enantiodetermining step. In this case, the origin of enantioselectivity arises from the steric repulsion between the substituent at the 3′-position of the BINOL skeleton and the aryl substituent of the ketone. Thus, the energy difference of 2.5 kcal mol^–1^ between both transition states in the hydride transfer step and the absolute configuration of the products are in agreement with the experimental results.

**Scheme 29:**
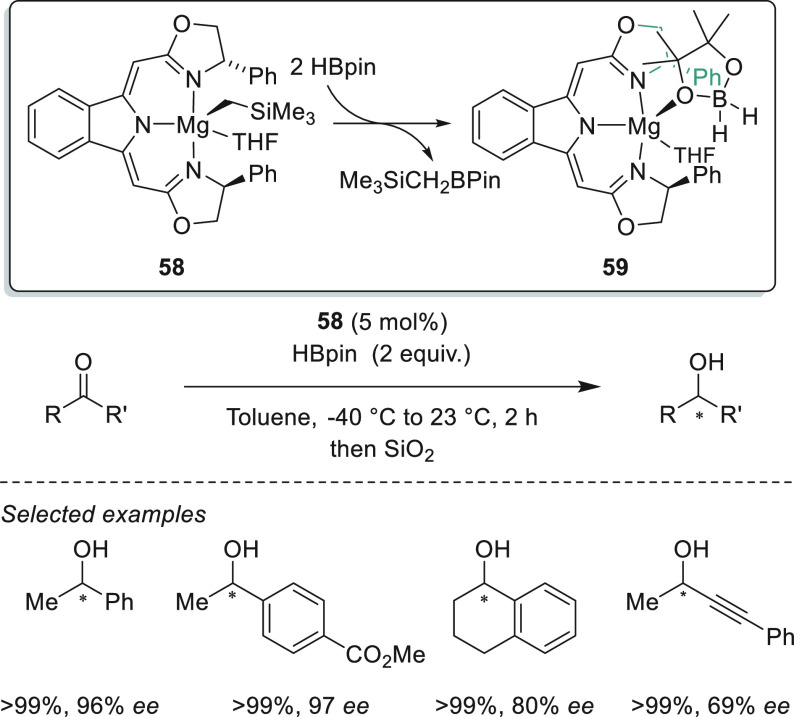
Enantioselective Mg-Catalyzed Hydroboration of Ketones Developed by Gade *et al.*

Similarly, Gade *et al.* developed magnesium–boxmi complex **58** (boxmi = bis(oxazolinylmethylidene)isoindoline) for the hydroboration of a wide range of acetophenone derivatives (Scheme 29).^109^ On the basis of the results of NMR spectroscopy and DFT calculations, the authors suggest a mechanistic pathway that involves a reactive borohydride intermediate (**59**) formed *via* metathesis with a simultaneous release of Me_3_SiCH_2_BPin. Although complex **58** showed excellent enantioselectivity toward acetophenone derivatives, competing favorably with Rueping’s catalyst **57** (Scheme 26), complex **58** showed a narrower substrate scope.

**Scheme 30:**
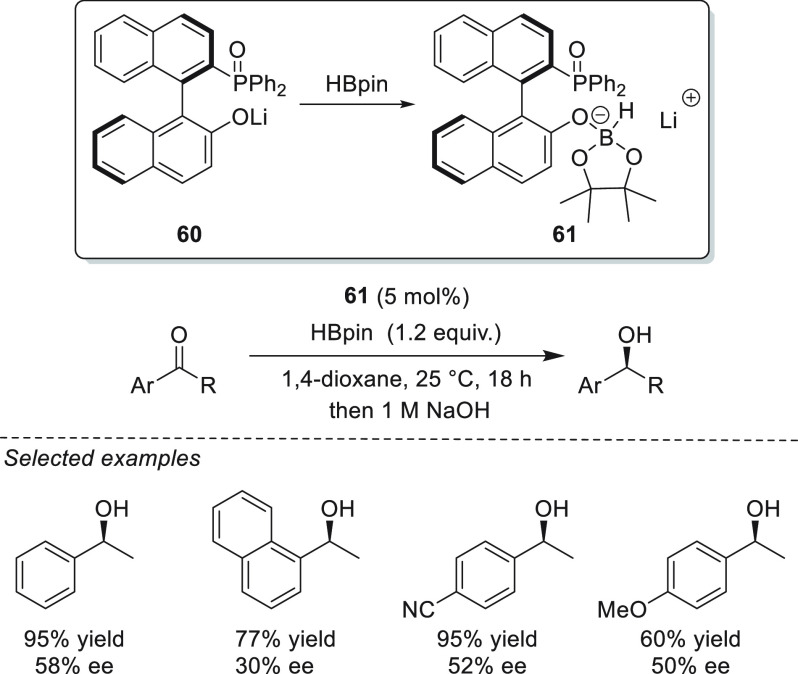
Lithium-Catalyzed Asymmetric Hydroboration of Aryl Alkyl Ketones

Very recently, Melen *et al.* developed the enantioselective lithium-catalyzed hydroboration of aryl alkyl ketones (Scheme 30). Lithium complex **60** formed *in situ* between LDA and a chiral BINOL-derived ligand generated secondary alcohols in good to excellent yields; however, the optical purities of the obtained products were rather low and did not exceed 60% ee. Mechanistically, the authors showed that the phenolic proton in the ligand is deprotonated with LDA and that subsequent addition of HBpin leads to the formation of reactive trialkoxyborohydride species **61**, which is responsible for the hydride transfer to the C=O bond.^110^

### 3.2 Pyridines

**Scheme 31:**
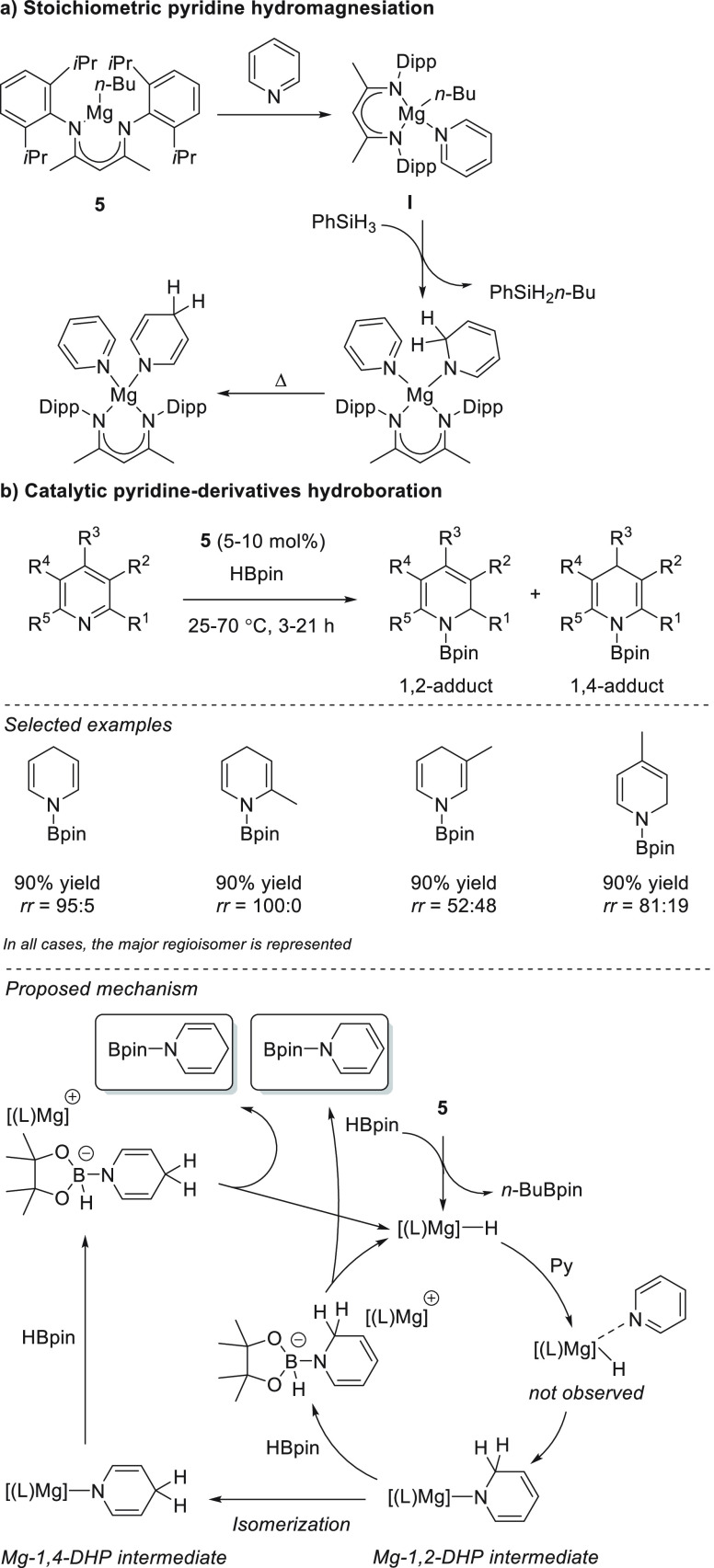
Magnesium-Catalyzed Hydroboration of Pyridine Derivatives

In 2010, Hill *et al.* found that β-diketiminate magnesium complex **5** promoted the dearomatization of pyridine. By single-crystal X-ray diffraction analysis and NMR spectroscopy, they observed that upon mixing of **5** and pyridine coordination takes place rather than alkyl addition. Treatment of the pyridyl complex with phenylsilane, a reagent known for the synthesis of well-defined magnesium hydrides, resulted the formation of *n*-butylphenylsilane. However, no evidence of a Mg–H species was found. Instead, the formation of a mixture of 1,2- and 1,4-dihydropyridines was observed (Scheme 31a).^111,112^ The product distribution was in agreement with the seminal work from Ashby who demonstrated MgH_2_ addition to pyridine.^113,114^

These stoichiometric studies led to the development of the first catalytic system based on magnesium hydride species for the hydroboration of unsaturated bonds. In this case, magnesium complex **5** was shown to catalyze the hydroboration of a wide range of pyridines, leading to mixtures of 1,2- and 1,4-dihydropyridine products (Scheme 31b), with a preference for the latter.^115,116^

Mechanistically, the magnesium-catalyzed hydroboration of pyridine derivatives did not differ from the stepwise stoichiometric studies previously accomplished by the same group. The authors postulate that the precatalyst **5** reacts with pinacolborane to afford the corresponding magnesium hydride intermediate, which upon pyridine coordination immediately forms the corresponding 1,2-dihydropyridine regioisomer (Mg–1,2-DHP), which isomerizes to the more thermodynamically stable regioisomer (Mg-1,4-DHP). Finally, the Mg–dihydropyridine intermediates reacts with pinacolborane, forming a magnesium borate intermediate that releases the final product and the regenerated magnesium hydride catalyst. It should be noted that the isomerization is highly substrate dependent, resulting in excellent to poor product regioselectivity.

**Scheme 32:**
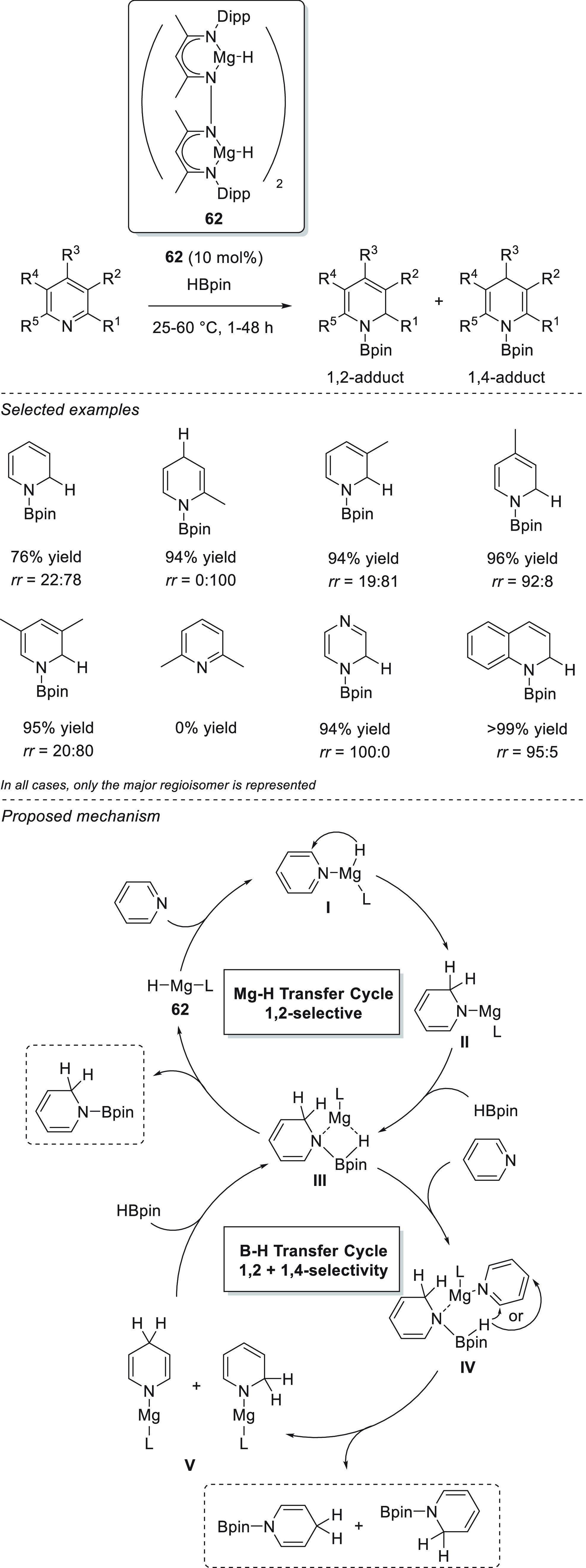
Multinuclear Magnesium Hydride Cluster for the Hydroboration of Pyridines

A multinuclear magnesium hydride cluster **62** was tested by Harder *et al.* for the conversion of pyridine derivatives to *N*-borylated dihydropyridines (Scheme 32).^117^ Focusing first on stoichiometric reactivity, a tetranuclear cluster **62** showed exceptional selectivity toward hydride transfer to the 2-position of the pyridine, with no isomerization even at elevated temperatures and prolonged heating. On the other hand, the octanuclear cluster analogue showed temperature-dependent mixtures with 1,2- and 1,4-selectivity. Because of the exceptional 1,2-selectivity, the authors applied **62** in the catalytic hydroboration of a wide range of pyridine derivatives. Whereas the use of stoichiometric Mg–H addition to pyridine led to the formation of a 1,2-regioisomer exclusively, the use of catalytic amounts of **62** resulted in lower regioselectivity. The inactivity observed for 2,6-lutidine supported the mechanistic hypothesis of an initial hydride transfer to the 2-position prior to the isomerization to the 4-position. The difference in regioselectivity of the stoichiometric and catalytic reactions led the authors to hypothesize that the two metallic centers of the catalysts might be operating in different catalytic stages of the cycle, which could potentially result in the preference of regioselectivity. The authors ruled out the possibility of the formation of catalytic amounts of MgH_2_ (which would lead to a nonselective hydride transfer) as no direct indication for Schlenk equilibrium was observed. Consequently, the authors proposed an alternative catalytic cycle, which circumvents the formation of the intermediate magnesium hydride species. Whereas the first catalytic cycle is based on a magnesium hydride species, which transfers the hydride to the 2-position selectively, the second, unselective cycle might be operating in parallel to the Mg–H cycle (Scheme 32). Thus, in the presence of excess pyridine (Cycle II), a magnesium borate intermediate (**IV**) is formed, which could directly transfer a hydride from boron to either the 2- or 4-position of a pyridine ligand, resulting in the formation of a magnesium 1,2-DHP or 1,4-DHP mixture (**V**). Importantly, multinuclear magnesium **62** showed slightly better performance than the mononuclear magnesium complex **5**.^115,116^

**Figure 5:**
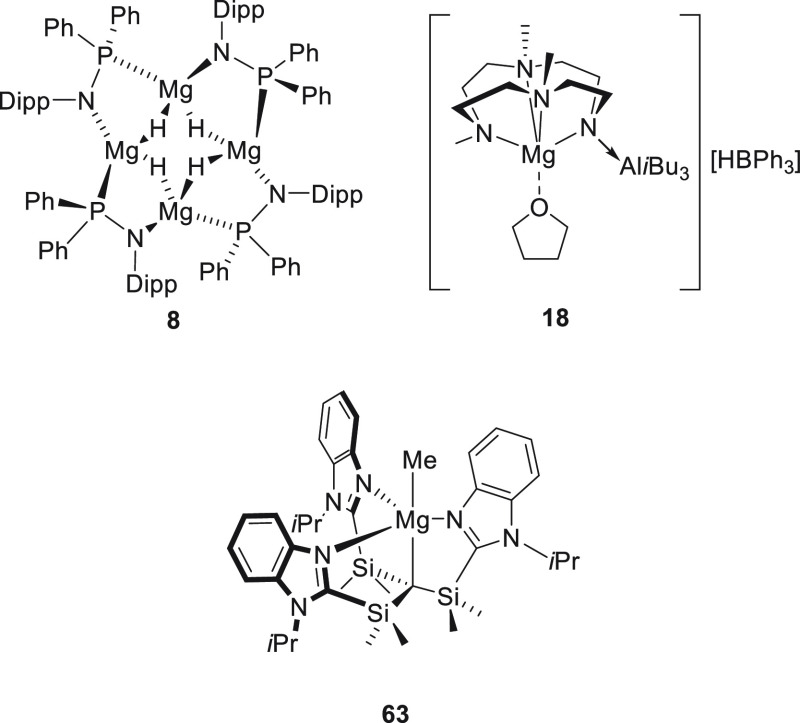
Complexes **8**, **18**, and **63** developed by Stasch, Okuda, and Parkin, respectively.

In 2016, Stasch *et al.* developed phosphinoamido–magnesium–hydride complex **8** (Figure 5), which was shown to be very active for the hydroboration of ketones.^75^ When applied to the hydroboration of pyridine, complex **8** did not lead to full conversion due to decomposition of HBpin under harsh reaction conditions, thus showing lower catalytic activity than the previously developed magnesium complexes **5** and **62** developed by Hill^115^ and Harder,^117^ respectively.

In the same year, Okuda *et al.* applied magnesium complex **18**, containing an *N,N,N,N*-type macrocyclic ligand (Figure 5), for the hydroboration of pyridine, which regioselectively afforded the 1,4-insertion product.^77^

Similarly, Parkin *et al.* developed [Tism^PriBenz^]MgMe complex **63** (Figure 5).^118^ Although the precatalyst provided the 1,4-addition product with high regioselectivity, the authors did not further investigate the substrate scope.

**Scheme 33:**
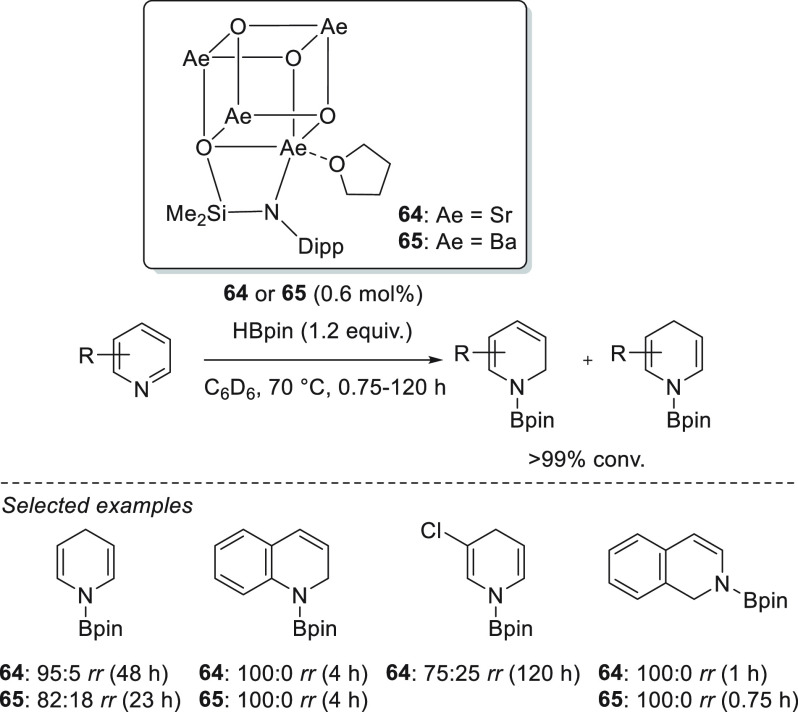
Sr- and Ba-Catalyzed Hydroboration of Pyridines and Quinolines

Tetranuclear siloxide/amide strontium complex **64**, which is active for the hydroboration of pyridine and its derivatives (Scheme 33), was reported by Harder *et al.*(119) In terms of activity and 1,4-regioselectivity, complex **64** competes favorably with the magnesium catalysts reported to date. The authors also developed tetranuclear siloxide/amide barium complex **65**, which is an analogue of **64**, for the hydroboration of pyridines. Compared to strontium complex **64**, barium complex **65** was slightly more active, although in some cases, this increase in activity occurred at the expense of regioselectivity. It is important to highlight the excellent activity and good selectivity of complex **65** when chlorinated pyridine was tested, which thus far has been converted only with the use of an iron catalyst.^120^

**Figure 6:**
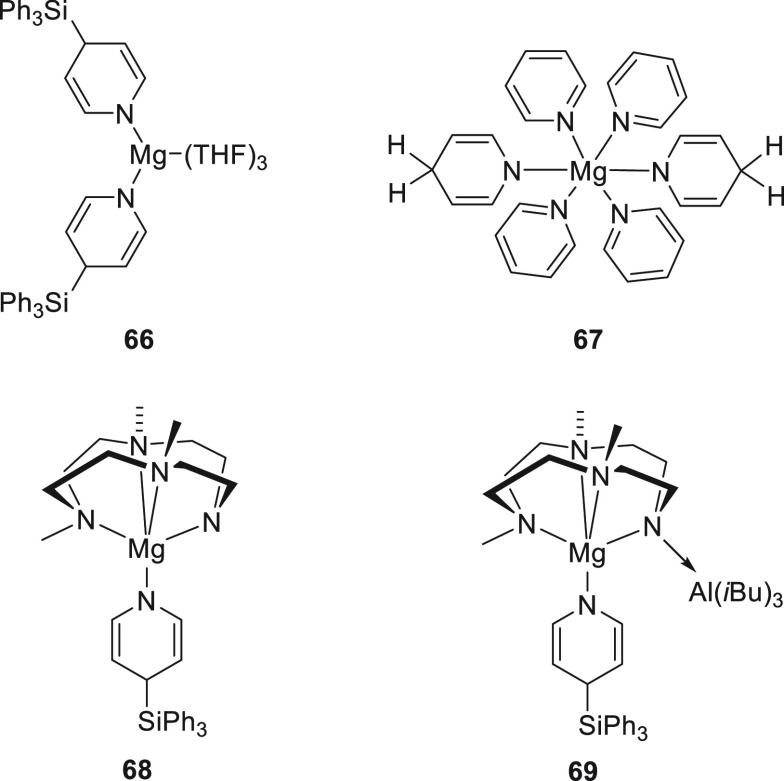
Complexes **66**–**69** developed by Okuda *et al.*

Okuda *et al.* then demonstrated that when [Mg(SiPh_3_)_2_(THF)_2_]·THF complex was mixed with pyridine it underwent 1,4-addition, affording complex **66** (Figure 6). A similar reaction but with excess pyridine provided complex **67**. The same result was observed when (Me_3_TACD)Mg(SiPh_3_) was mixed with excess pyridine, leading to the formation of complex **68**. For comparison purposes, (Me_3_TACD·Al*i*Bu_3_)Mg(NC_5_H_4_SiPh_3_) **69** was synthesized, and all complexes (**67**–**69**) were applied in the hydroboration of pyridine. Complexes **68** and **69** were the most active complexes.^121^ Regarding activities and regioselectivities, these complexes showed behavior similar to those already reported by Stasch *et al.* (**8**),^75^ Hill *et al.* (**5**),^115^ and Harder *et al.* (**62**).^117^

**Figure 7:**
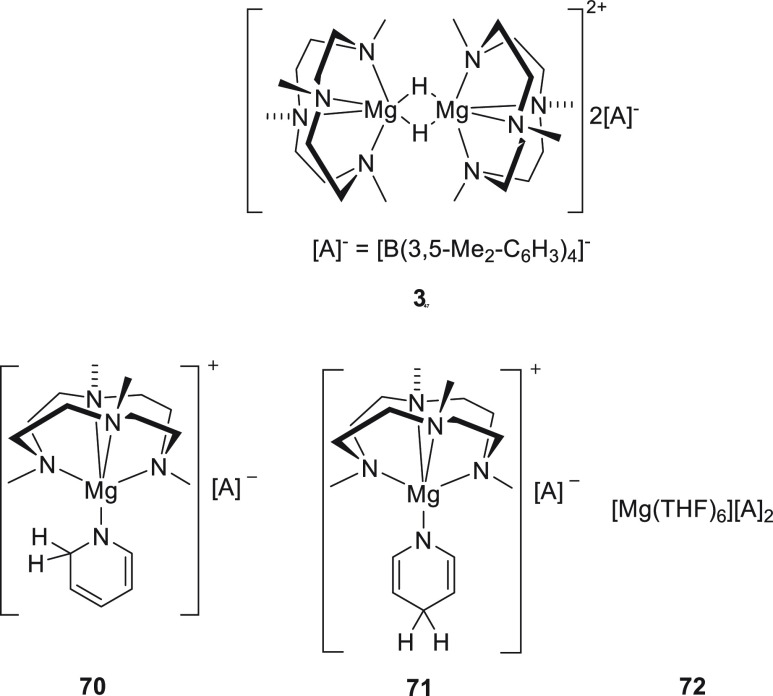
Magnesium species applied for the hydroboration of several polarized unsaturated bonds.

Okuda *et al.* also reported the synthesis of cationic magnesium hydride species **3** (Figure 7) stabilized by an *N,N,N,N*-type macrocycle.^46^ When **3** was treated with pyridine, complex **70** was obtained. Remarkably, complex **70**, which contains a 1,2-dihydropyridine as a ligand, did not isomerize to the 1,4-regioisomer **71**, even at high temperature, in excess pyridine, and after a long time. The conversion of **70** to **71** was achieved by adding catalytic amounts of complex **72**. The authors speculated that the strong Lewis acidic Mg^2+^ complex **72** accelerated the transformation of **70** to **71**. These three magnesium complexes were further applied for the catalytic hydroboration of pyridine. Whereas complexes **70** and **71** provided a mixture of 1,2- and 1,4-regioisomers (ratio 1:3 and 1:9, respectively), complex **72** exclusively provided the 1,4-regioisomer.

**Scheme 34:**
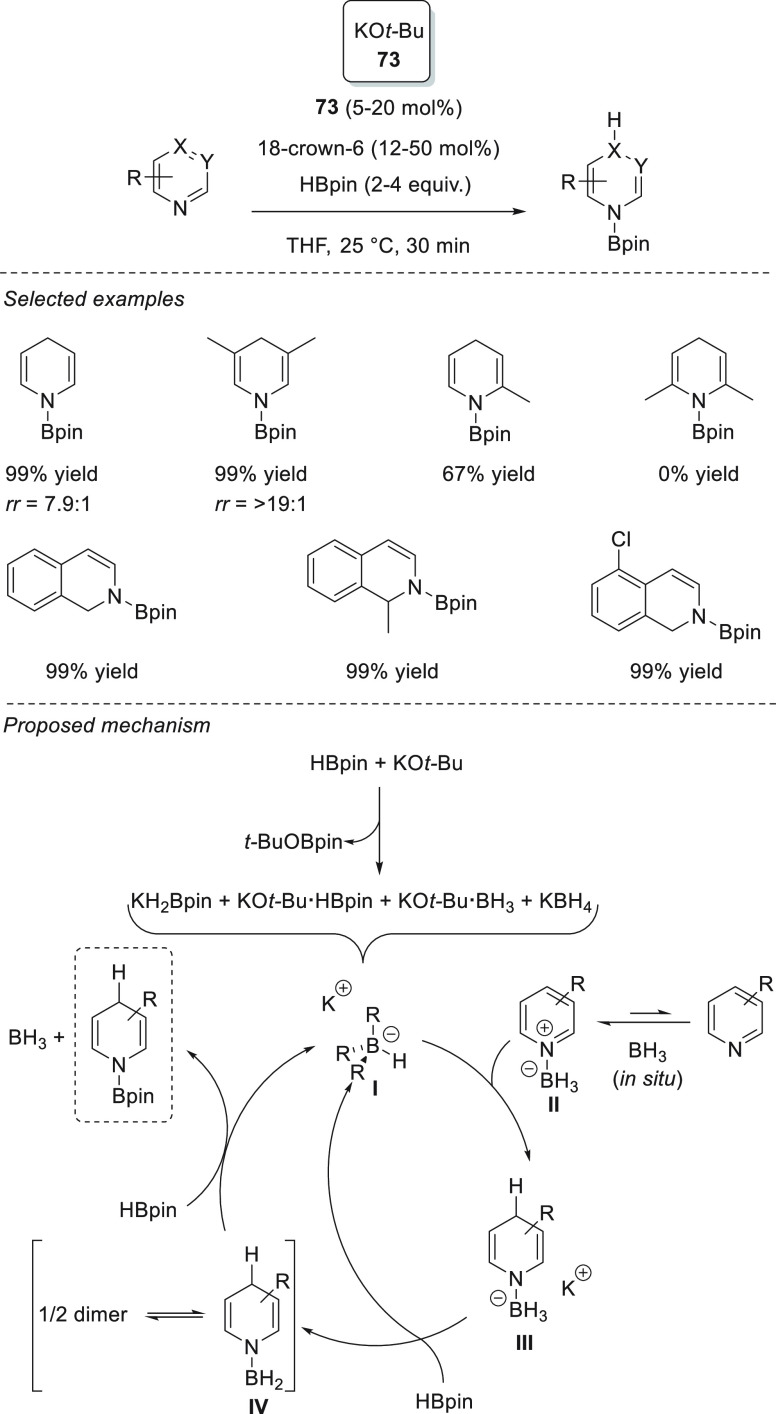
K-Catalyzed 1,4-Regioselective Hydroboration of *N*-Heterocycles

Interestingly, Park and Chang *et al.* found that potassium-based precatalysts not only are active for the hydroboration of C=O bonds but also provide excellent regioselectivities for the hydroboration of pyridines and their derivatives. Potassium *tert*-butoxide **73** together with 18-crown-6 showed excellent activities and regioselectivities for the hydroboration of a range of *N*-heteroarenes (Scheme 34), achieving *N*-boryl-1,4-dihydropyridines in excellent yields. Mechanistic studies revealed that *in situ* formed BH_3_ forms an adduct with *N*-heteroarenes to which HBpin is selectively added to break the *N*-aromaticity. Mechanistic investigations supported by NMR spectroscopy, DFT calculations, and kinetic studies revealed that initially KO-*t*-Bu reacts with HBpin to rapidly produce borohydrides species **I**, which are in equilibrium (including BH_3_). The pyridine substrate then reacts with BH_3_ to generate a pyridine·BH_3_ adduct **II** that undergoes nucleophilic hydride attack by a borohydride species, forming 1,4-dihydropyridyl borohydride **III**, which is a resting intermediate. Then a hydride transfer from **III** to HBpin slowly regenerates the reactive borohydrides **I** and 1,4-dihydropyridyl borane **IV**, which finally reacts with a second molecule of pinacolborane to afford the *N*-Bpin-1,4-dihydropyridine product and BH_3_, probably *via* σ-bond metathesis.^122^

**Scheme 35:**
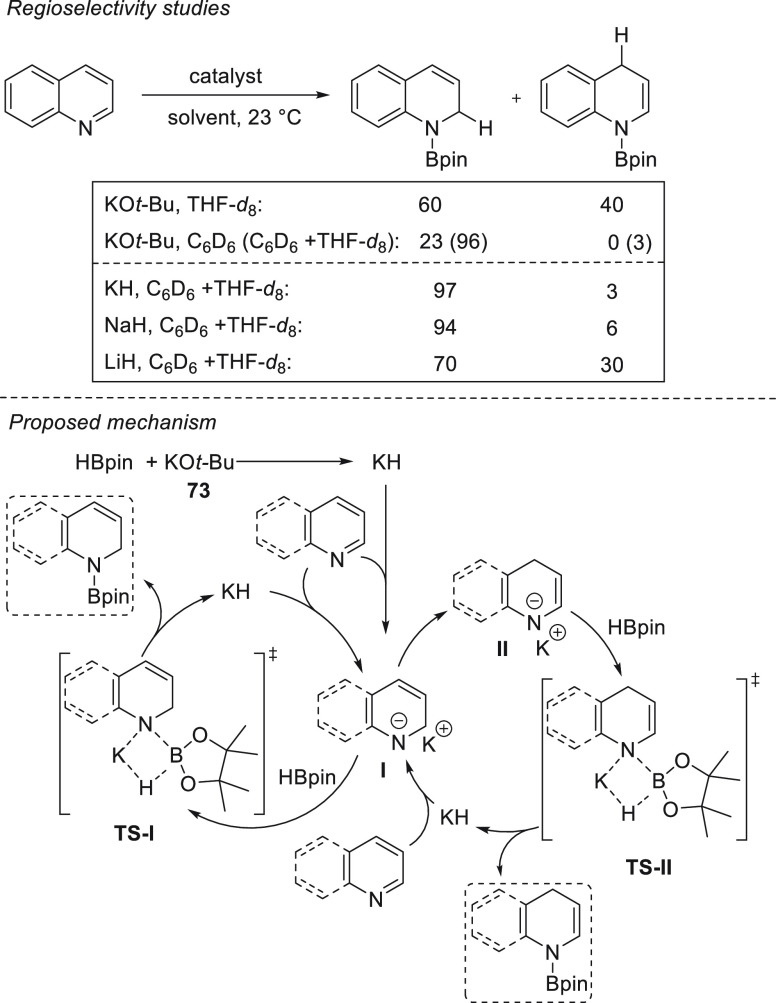
K-Catalyzed Hydroboration of Pyridines and Quinolines. Kinetic (Left) and Thermodynamic Processes (Right)

Recently, He and Zhang *et al.* reported the regioselective 1,2-hydroboration of *N*-heteroarenes using potassium *tert*-butoxide **73**.^123^ The authors found that replacing THF with a nonpolar solvent, such as benzene, completely changed the regioselectivities observed by Chang *et al.*(122) toward selective 1,2-addition. Therefore, a range of *N*-heteroarenes could be selectively hydroborated, affording the corresponding *N*-boryl-1,2-dihydropyridine derivatives in excellent yields and selectivity. When KO-*t*-Bu **73** and HBpin were mixed a white precipitate was obtained together with *t*-BuOBpin. The authors suggested that the white precipitate is KH, which indeed in subsequent experiments performed the same way as KO-*t*-Bu **73** (Scheme 35). Interestingly, other alkali metal hydrides (LiH and NaH) provided lower regioselectivity. These findings corroborated the hypothesis that KH is formed after mixing **73** and HBpin and that KH was the active hydride species.

Regarding the regioselectivity, the authors propose that the 1,2-regioselectivity relies on the reaction between K-compound **I** with HBpin, which is faster than the isomerization of **I** to **II**. Therefore, once **I** is formed, the 1,2-regiosomer will be afforded together with KH. Control experiments confirmed that the isomerization from **I** to **II** occurs due to stability of *N*-boryl-1,2-hydropyridine (Scheme 35).^123^ Hence, polar and coordinative solvents such as THF favor the isomerization toward intermediate **II**, whereas C_6_D_6_ suppresses it.

A comparison of the KO-*t*-Bu-catalyzed hydroboration of *N*-heterocycles reported by Park and Chang *et al.*(122) and He and Zhang *et al.*(123) shows that the active hydride species (and thus, the regioselectivity) depends on the nature of the solvent. Whereas reactions carried out in THF and in the presence of 18-crown-6 provide BH_3_ as active hydride species and 1,4-regioisomers, the reactions carried out in C_6_D_6_ and in the absence of 18-crown-6 form KH as a hydride donor, and 1,2-regioisomers are obtained.

**Scheme 36:**
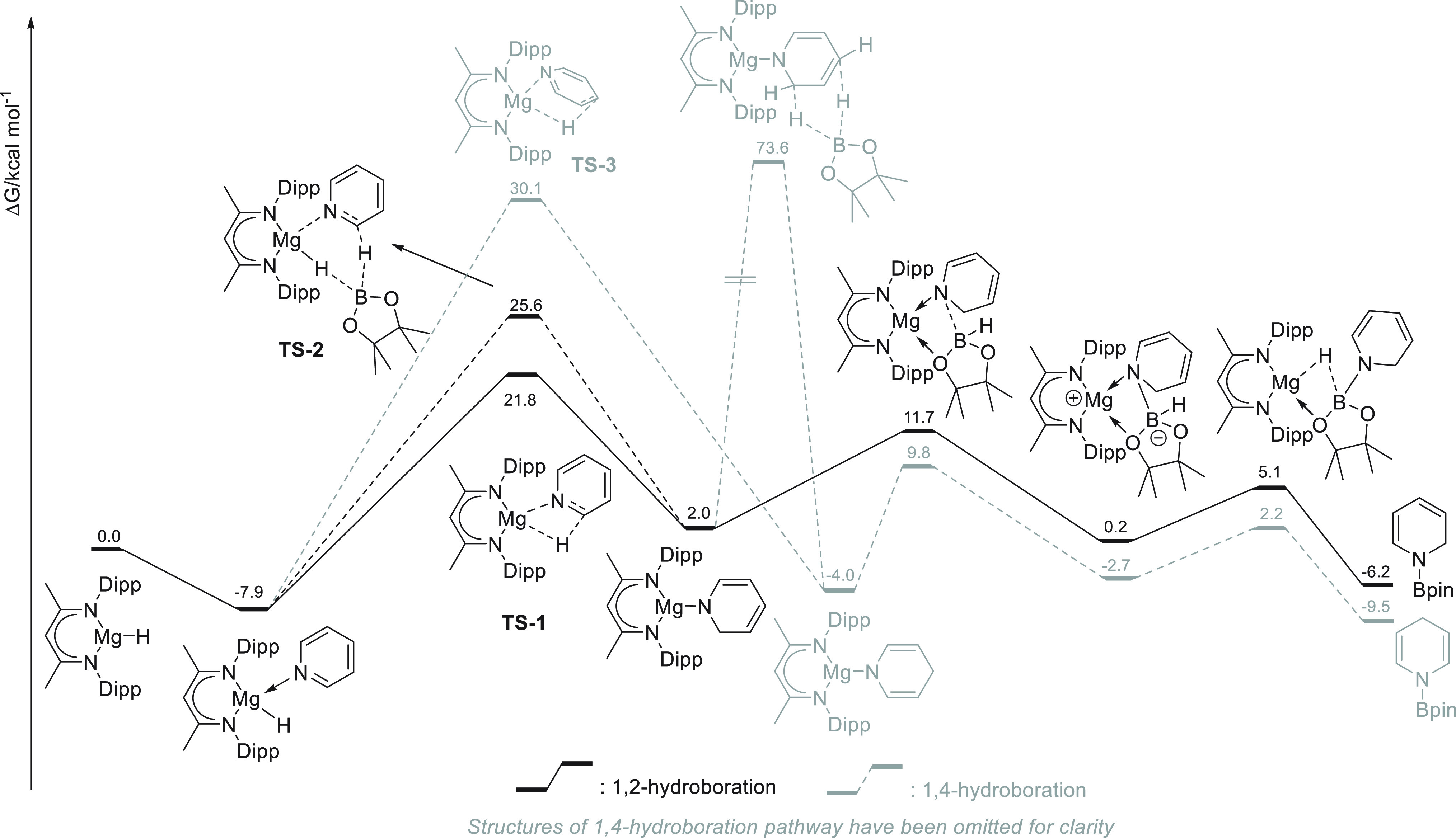
Mechanistic Insight into Mg-Catalyzed Hydroboration of Pyridine

Bai and Lan and co-workers performed DFT calculations to investigate the mechanism of the alkaline earth metal catalyzed hydroboration of pyridines with pinacolborane.^124^ The authors studied the magnesium-catalyzed hydroboration of pyridine using one of the most employed catalysts: a magnesium hydride derived from the precatalyst **5** (Scheme 36). The authors established that once the magnesium hydride species is formed (upon mixing **5** with pinacolborane) pyridine coordination takes place. The authors studied three possibilities: (i) direct 1,2-hydride transfer involving Mg–H; (ii) 1,2-hydride transfer mediated by pinacolborane; and (iii) direct 1,4-hydride transfer involving Mg–H. Interestingly, the insertion barrier steps are of 29.7, 33.5, and 38.8 kcal mol^–1^, respectively. The hydride transfer *via***TS-1** and **TS-2** affords the same Mg-1,2-dihydropyridine intermediate, whereas **TS-3**, less energetically favored, provides the other regioisomer. The free energy profile shows that the rate-determining step of the catalytic cycle is the hydride transfer to pyridine (*via***TS-1**). The authors also studied other alkaline earth metal catalysts and found that the activation free energies of **TS-1** are much lower for the Ca and Sr analogue of **5** (calculated Ae-H BDE is Be-H > Mg-H > Ca-H > Sr-H). Thus, the authors anticipate that calcium and strontium catalysts could be used in a future, allowing milder reaction conditions.

### 3.3 Imines

**Scheme 37:**
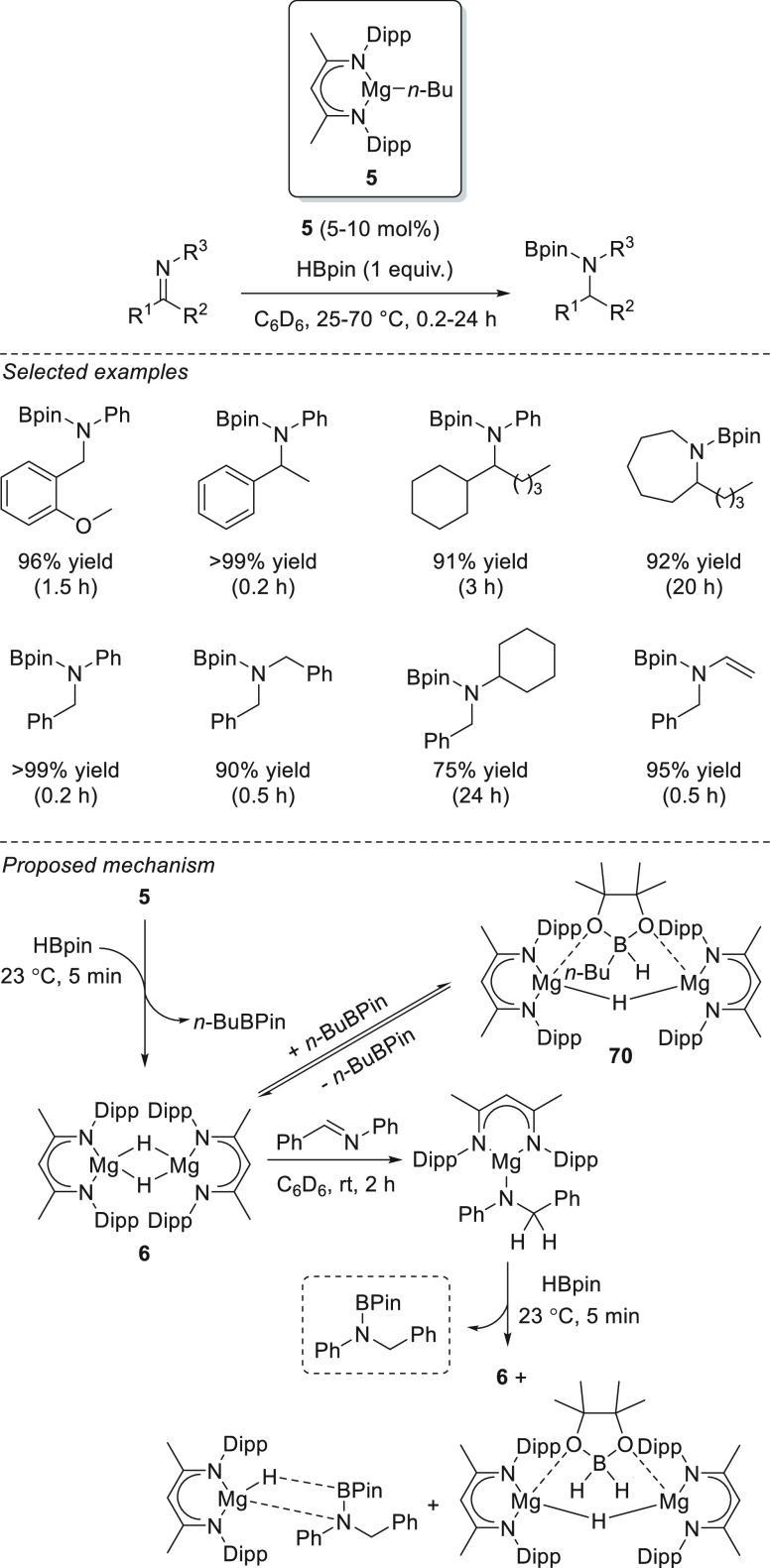
Magnesium-Catalyzed Hydroboration of Imines

Imines, which are easily accessible from carbonyl compounds and primary amines, are suitable precursors for the preparation of secondary amines. In 2013, Hill *et al.* reported the excellent catalytic activity of magnesium complex **5** for the hydroboration of *N*-aryl and *N*-alkyl aldimines and ketimines (Scheme 37).^125^ For reactions that required temperatures higher than 60 °C and prolonged times, poor yields were obtained due to the decomposition of catalyst. The authors observed in several reactions B_2_pin_3_ as a decomposition pathway byproduct.

By means of NMR spectroscopy, the authors established that when complex **5** is mixed with HBpin, dimeric species **6** is formed together with *n*-BuBpin, which exist in equilibrium with the magnesium borohydride complex **70**. This active Mg-H species **6** undergoes C=N bond addition affording a magnesium-amido intermediate, and after σ-bond metathesis with 1 equiv of HBpin, *N*-boryl amine is obtained, and active species **6** is partially regenerated together with a new magnesium hydride species (Scheme 37).

Kinetic studies displayed a second-order rate in [imine] and a zero order in [HBpin], and in the excess of pinacolborane, a decrease of the reaction rate was observed, consistent with HBpin acting as an inhibitor. On the contrary, the excess of imine substrate showed no catalyst inhibition at higher initial concentrations. Finally, kinetic studies showed that the reaction is first order in [catalyst], which is consistent with the monomeric nature of the insertion intermediate.

**Scheme 38:**
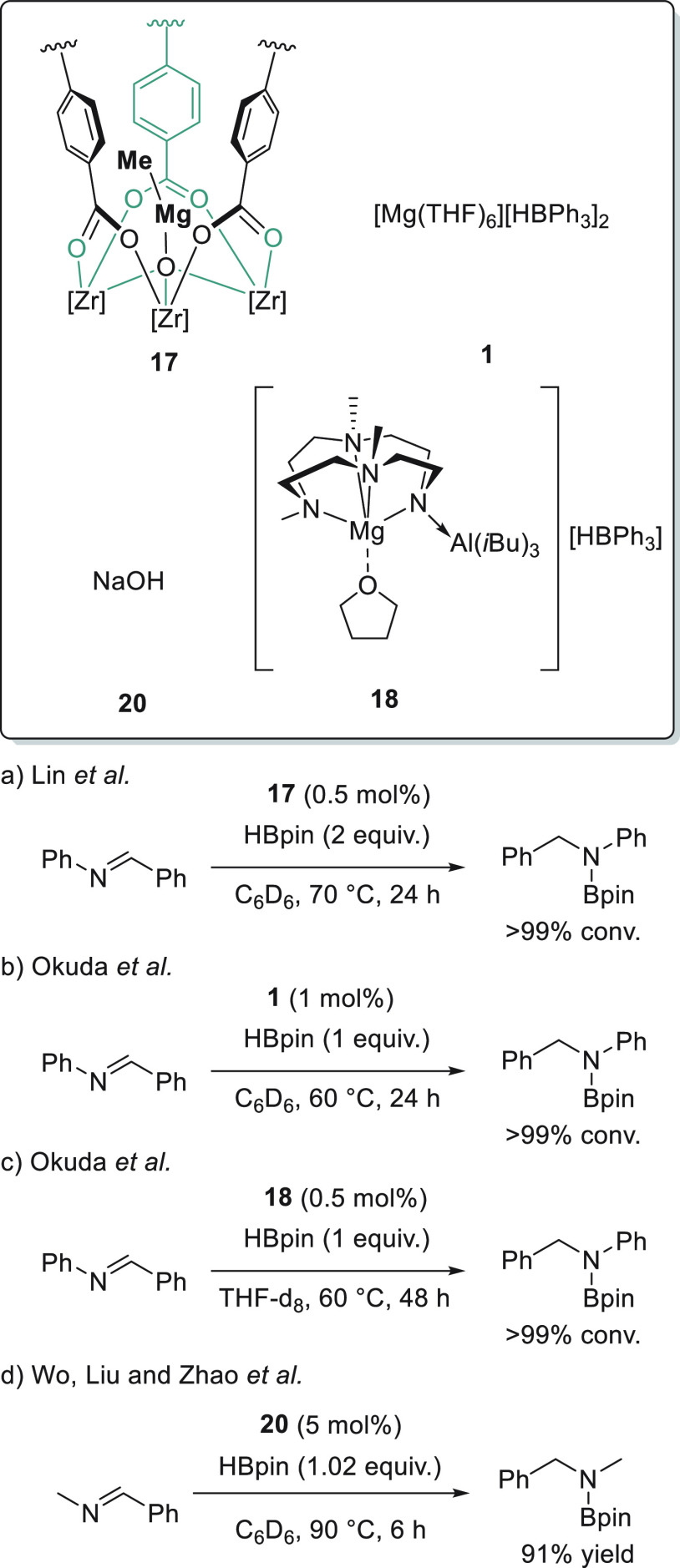
Hydroboration of Various Benzaldehyde-Derived Aldimines

Lin *et al.* applied magnesium-functionalized Zr-MOF **17** for the hydroboration of imines to give *N*-borylamines.^76^ Although the complete conversion of *N*-benzylideneaniline was observed with a catalyst loading of 0.05 mol %, rather long reaction times were reported for other aldimines and ketimines, limiting the applicability of the catalyst (Scheme 38a).

Magnesium hydrotriphenylborate complex **1** applied for the reduction of a variety of polarized bonds was used for *N*-benzylideneaniline as a model substrate (Scheme 38b).^45^ Similar to Lin’s finding,^76^ harsher reaction conditions and a long reaction time were necessary to afford full conversion of this relatively reactive imine.

*N*-benzylideneaniline was hydroborated by Okuda *et al.* in the presence of magnesium complex **18** containing an *N,N,N,N*-type macrocyclic ligand (Scheme 34c).^77^ The authors suggested that the presence of a bulky Al(*i*Bu)_3_ group made the reaction difficult, and full conversion was achieved after 48 h.

The catalytic hydroboration of *N*-methyl-1-phenylmethanimine could be initiated simply by NaOH **20** (Scheme 34d), as shown by Wu, Liu, and Zhao *et al.*(79) The reaction proceeded within 6 h at 90 °C, affording the product in good yield. The authors, however, did not expand the substrate scope to other imines.

**Scheme 39:**
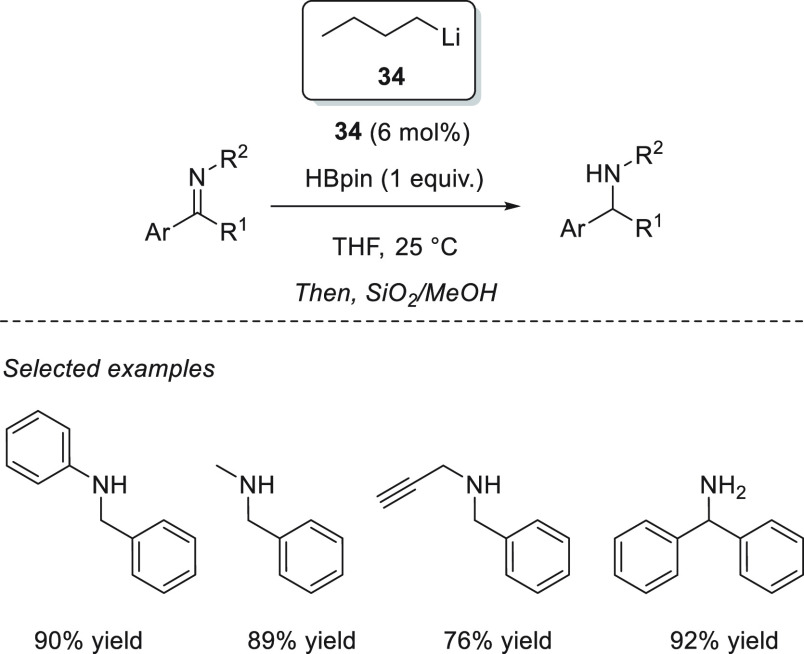
Li-Catalyzed Hydroboration of Imines

Furthermore, Xue and Bao *et al.* extended the application of *n*-BuLi **34** as an efficient precatalyst for the hydroboration of imines (Scheme 39).^126^ For the first time, a lithium catalyst was shown to be active toward the hydroboration of imines. A wide range of *N*-aryl and *N*-alkyl aldimines and ketimines were hydroborated under mild reaction conditions. Additionally, this catalytic system showed excellent chemoselectivity toward the hydroboration of *N*-propargylic aldimines.

Panda and co-workers applied potassium benzyl (KCH_2_Ph) **74** for the hydroboration of aldimines.^127^ Although low catalyst loadings (5 mol %) and mild reaction conditions were required for the successful hydroboration of aldimines, no examples of less reactive *N*-alkyl aldimines and ketimines were presented. Thus, excellent yields were obtained for a wide range of *N*-aryl ketimines. Furthermore, after successfully applying lithium bromide for hydroboration of aldehydes and ketones in the presence of cheap and readily available lithium bromide **53**,^97^ An *et al.* utilized this catalytic system for hydroboration of imines. Various aryl-protected aldimines and ketimines were hydroborated to afford the corresponding amines under mild reaction conditions and in remarkably short reaction times.^128^

### 3.4 Esters and Amides

Due to their higher stability compared to ketones and imines, the reduction of esters and amides is a more challenging task.^129^ The existing protocols often employ either transition-metal catalysts^130^ or very reactive metal hydrides,^131^ which are rather undesired, as they tend to react with other functional groups. The s-block metal-catalyzed hydroboration of esters and amides has been reported using only magnesium-based catalysts.

**Scheme 40:**
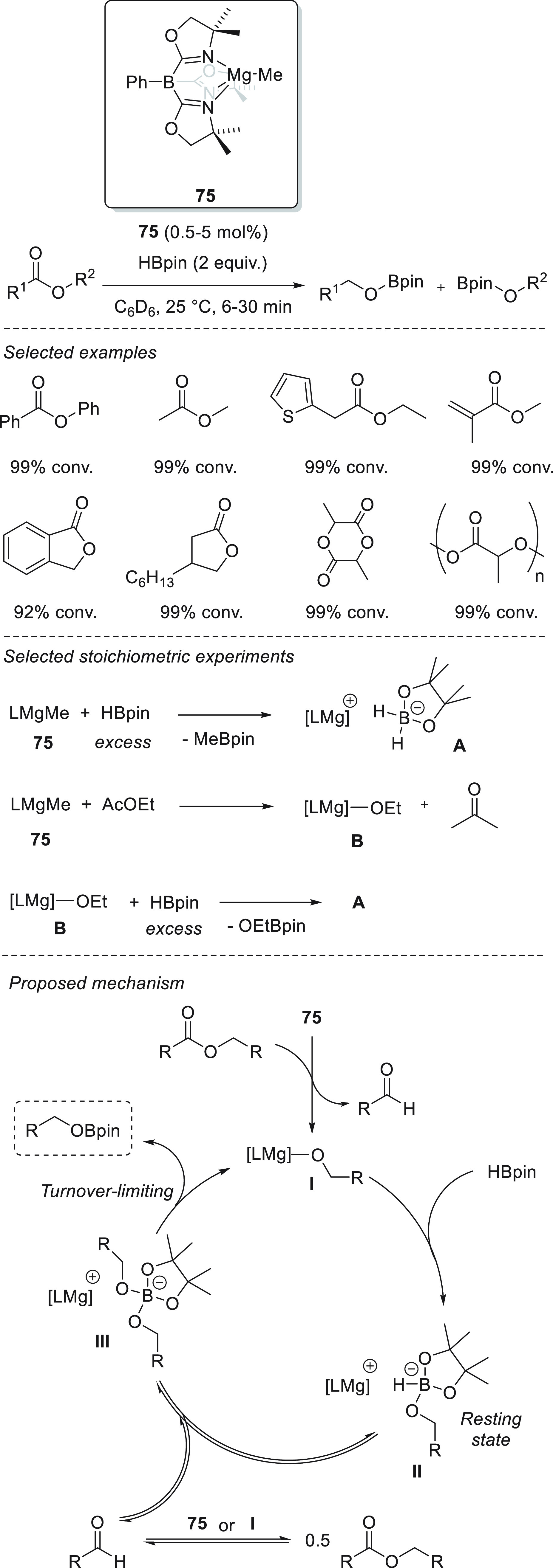
Magnesium-Catalyzed Hydroboration of Esters by Sadow **et al.**

The first example of magnesium-catalyzed hydroboration of esters was reported in 2014 by Sadow *et al.* Trisoxazonylphenylborate magnesium complex **75** (To^M^MgMe) was used for the catalytic hydroboration of both linear and cyclic esters. In all cases, quantitative conversions were observed under mild reaction conditions, short reaction times and low catalyst loadings (Scheme 40).^132^ Moreover, the authors established the formation of a zwitterionic magnesium borohydride species **A** when **75** reacts with the excess of pincacolborane. Here, the magnesium center coordinates to the O atom of the pinacolborane and the two hydrogens are bound the boron atom. The reaction of **75** with AcOEt produces magnesium ethoxide **B** within 5 min, following the elemental organometallic steps of MgMe addition to C=O bond to give the β-ethoxy alkoxide, followed by the β-ethoxide elimination. Unexpectedly, kinetic studies of the catalytic reaction ruled this step out as a part of the catalytic cycle. Treatment of the magnesium ethoxide species **B** with the excess of pinacolborane provides zwitterionic magnesium borohydride species **A** and EtOBpin. Notably, this step is also ruled out by catalytic kinetic experiments.

Kinetic studies revealed surprising dependencies of half-order [AcOEt] and zero-order [HBpin]. The half-order [AcOEt] indicates a reversible interaction of the catalyst and the ester substrate to afford ester cleavage prior to the turnover-limiting step. The zero-order [HBpin] rules out that the classic σ-bond metathesis mechanism is turnover-limiting and together with the half-order [AcOEt] unambiguously rules out the insertion/σ-bond metathesis pathway. The Mg intermediate **II** was shown to be the resting state, as it was the only magnesium compound observed during the course of the catalytic reaction. This finding is in agreement with the zero-order [HBpin] as the hydride reducing agent is not HBpin but **II** in its resting state. Thus, the classic σ-bond metathesis step forming the corresponding Mg-H and EtOBpin species does not occur. The half-order [AcOEt] rather indicates that the catalyst resting state interacts reversibly (*via* reversible ester cleavage). Finally, product formation occurs by a unimolecular conversion of intermediate **III** that already contains Bpin^–^, RO^–^, and LMg^–^ moieties. In addition, the authors showed that either **75** or magnesium intermediate **I** can mediate ester metathesis, a fast reversible cleavage of ester substrate providing the corresponding aldehyde.

**Scheme 41:**
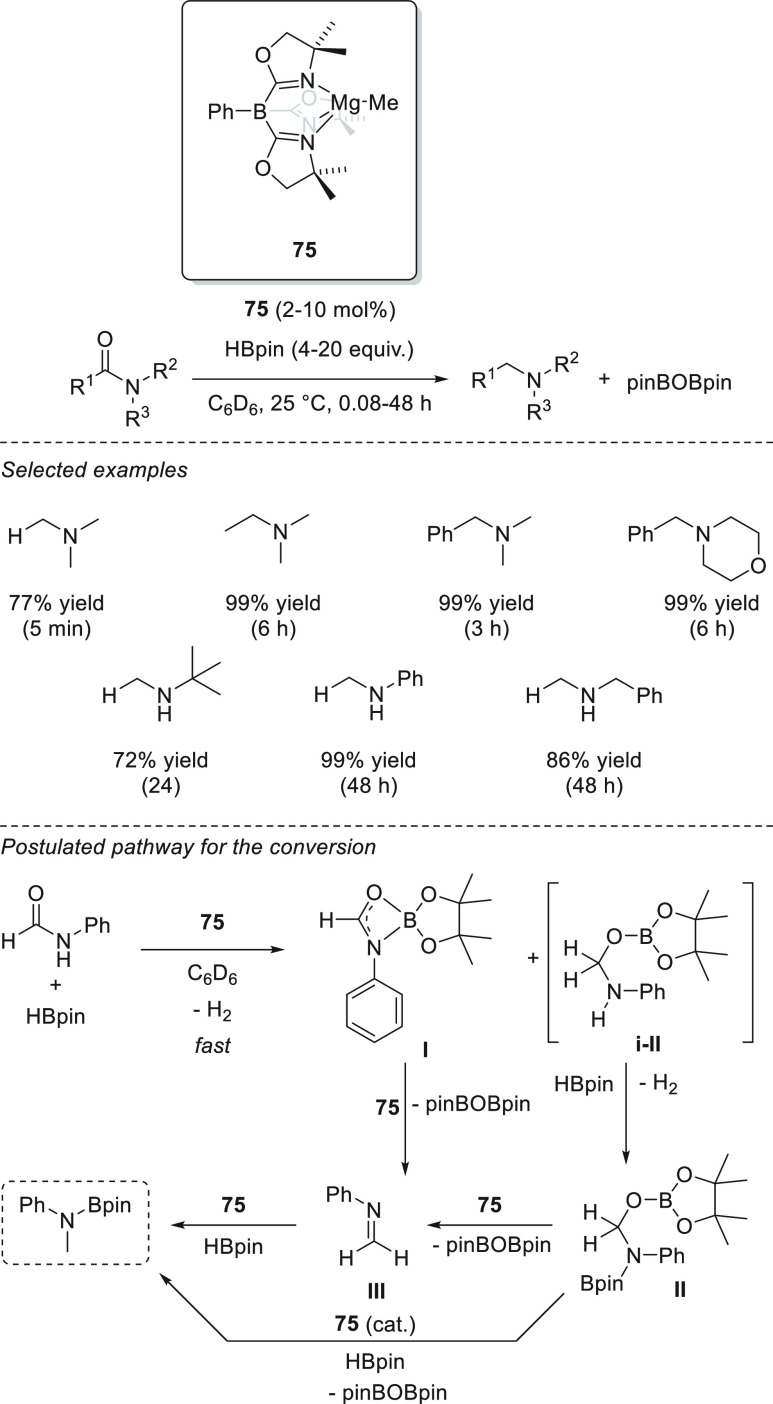
Magnesium-Catalyzed Deoxygenative Reduction of Amides by Sadow *et al.*

One year later, in 2015, the same authors applied complex **75** to the hydroboration of amides (Scheme 41). The presented catalytic system allowed the reduction of secondary and tertiary amides to the corresponding amines *via* deoxygenative C–O bond cleavage.^133^ Although reduction of *N*,*N*-dimethylformamide was completed within minutes under mild reaction conditions, hydroboration of acetamides, benzamides, and secondary formamides required much longer reaction times of up to 48 h. Mechanistically, the authors postulate that the magnesium species **A** (in Scheme 40), formed by mixing **75** and HBpin, is not a plausible catalytically relevant species as it provides a mixture of several products from C–N, C–O, and C–C bond cleavages. Alternatively, **75** decomposes in the presence of amides, ruling out the possibility that **75** is a catalytically relevant species. Moreover, no reactivity of amide with HBpin (in the absence of **75**) is observed.

Spectroscopy data suggest that **75** reacts with both amide and HBpin simultaneously, forming formimidate boronic ester **I** (at higher concentrations of HBpin) and diborylated compound **II** (at lower concentration of HBpin); the latter being obtained *via* a NH/BH dehydrocoupling pathway from a possible **i-II** species. Kinetic studies showed that conversion of amide to **I** and **II** is fast, whereas the reductive deoxygenation pathway (from **I** and **II** to give the desired product) is the turnover-limiting step.

**Scheme 42:**
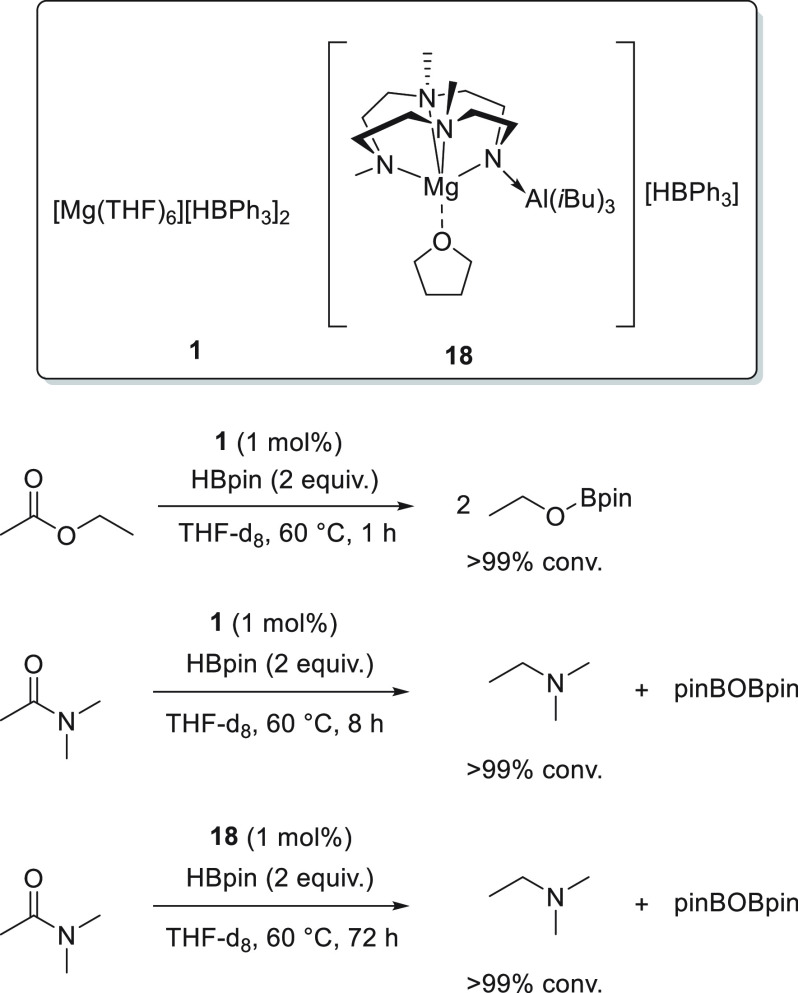
Deoxygenative Reduction of Esters and Amides by Okuda *et al.*

Okuda *et al.* also applied magnesium hydrotriphenylborate complex **1** and complex **18** for the hydroboration of esters and amides (Scheme 42).^45,77^ In all cases, however, the reactions required harsher conditions and longer times than those needed for Sadow’s precatalyst **75**.^132,133^ The authors concluded that in the case of complex **18**, the presence of a bulky Al(*i*Bu)_3_ group reduces the amide reactivity.

**Figure 8:**
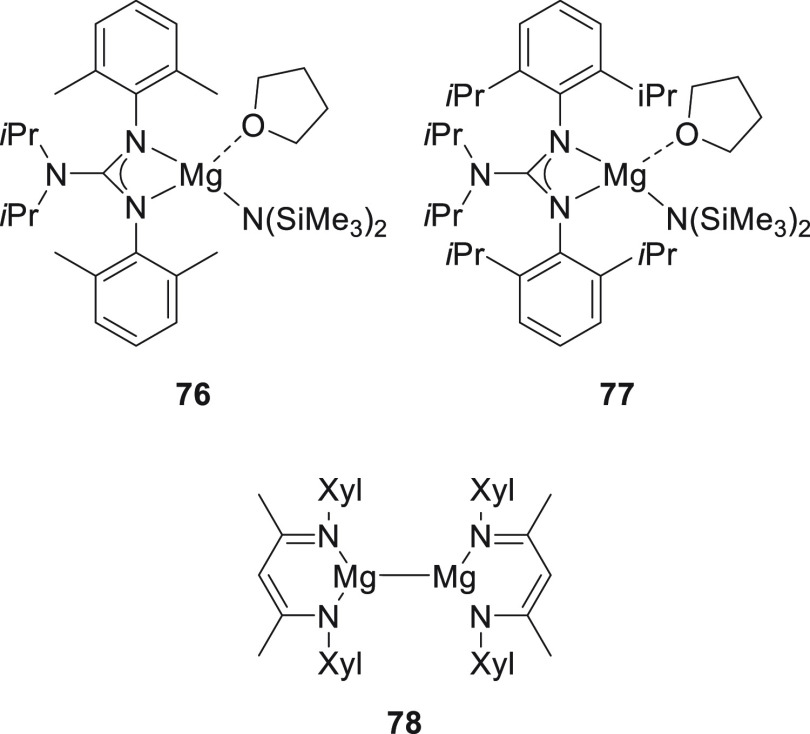
Magnesium complexes for hydroboration of esters.

Several magnesium amide complexes **76** and **77** (Figure 8) were developed in 2017 by Nembenna *et al.* for the selective hydroboration of esters.^134^ Whereas magnesium diamide **76** showed excellent selectivity toward ester hydroboration, more sterically hindered complex **77** was less active. From a catalytic perspective, quantitative conversions at ambient temperature and low catalyst loadings (0.1–0.5 mol %) were achieved in 10–45 min for a wide range of linear and cyclic esters bearing functional groups, competing favorably with Sadow’s precatalyst.^132^

Ma *et al.* applied dimeric Mg(I) complex **78** (Figure 8) for the reduction of various carbonyl compounds, including esters.^135^ Under mild reaction conditions, full conversions were obtained for a variety of substrates, showing the high effectiveness of this low-valent magnesium(I) complexes, comparable to the best results obtained with divalent magnesium(II) complexes.

**Scheme 43:**
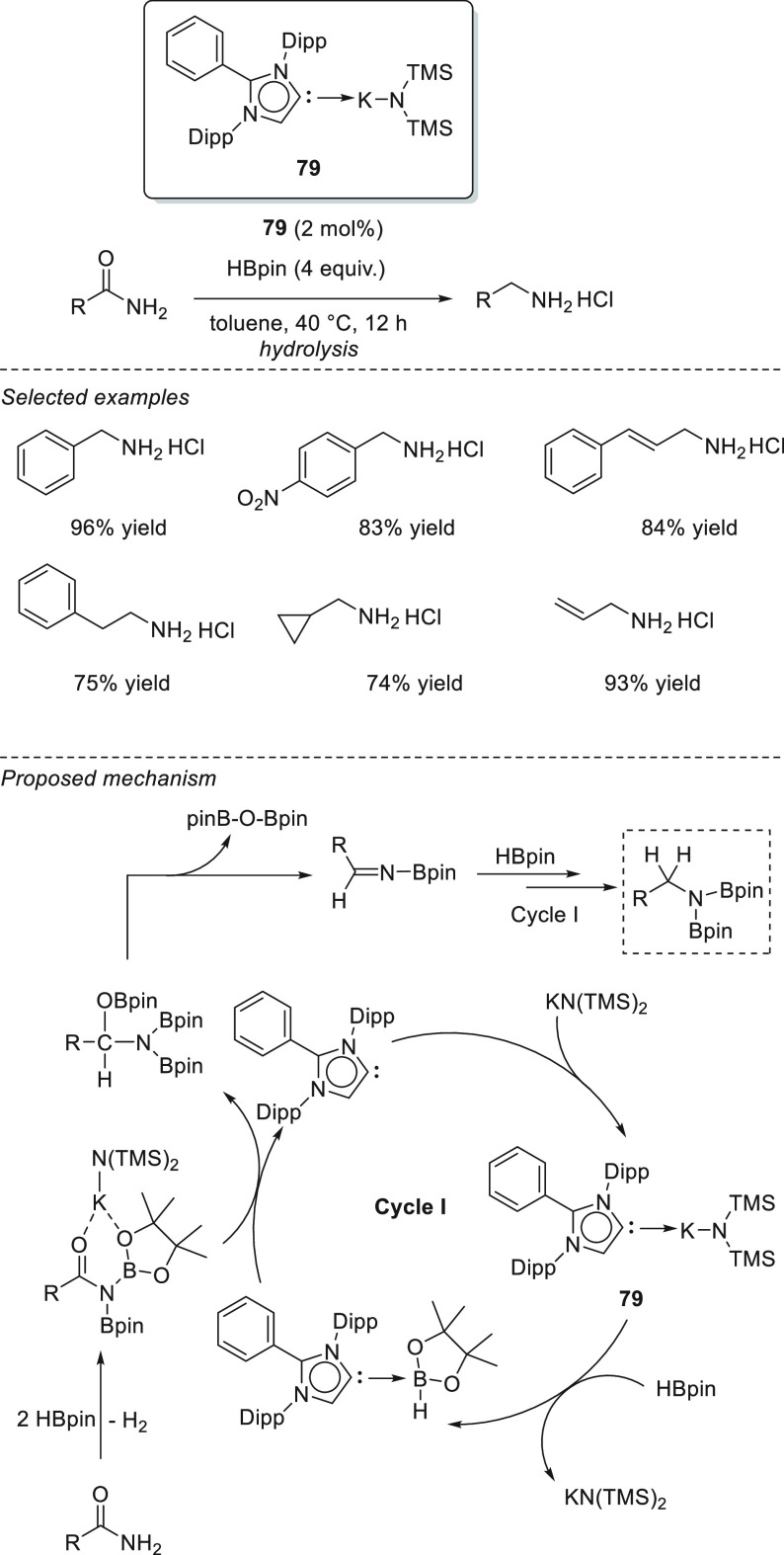
Hydroboration of Primary Amides by Mandal *et al.*

Mandal and co-workers reported the use of abnormal NHC-based potassium complex **79** for the catalytic hydroboration of primary amides.^136^ Low catalyst loading and mild reaction temperatures allowed the reduction of several aryl and alkyl primary amides in excellent yields. It is important to highlight that control experiments showed that in the absence of precatalyst **79** or with the presence of only NHC or KN(TMS)_2_ no conversion was observed. Isolation of reaction intermediates and single-crystal XRD analysis as well as DFT calculations led to the proposal of the dual role of **79**: nucleophilic activation of HBpin by the abnormal NHC and the Lewis acidic activation of the borylated amide by potassium ions (Scheme 43).

**Scheme 44:**
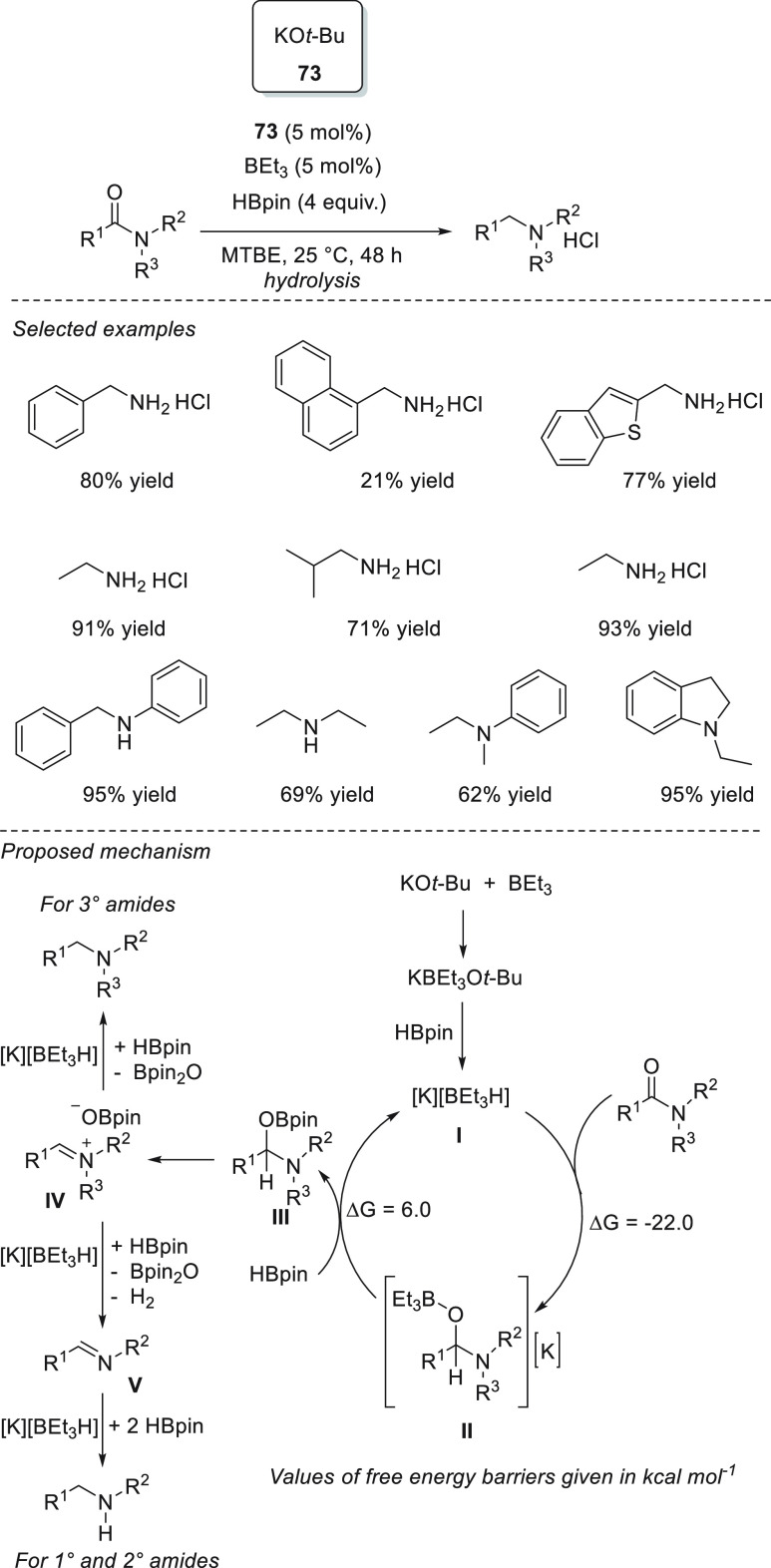
Hydroboration of Amides by Yao *et al.*

Later, Yao and co-workers reported that simple and commercially available KO-*t*-Bu **73** in combination with BEt_3_ could selectively reduce amides to amines (Scheme 44).^137^

Interestingly, and contrary to the previously reported K-based precatalyst **79**, KO-*t*-Bu **73** in combination with BEt_3_ catalyzes the deoxygenative reduction of tertiary, secondary, and primary amides with pinacolborane, although longer reaction times were required. Kinetic experiments showed that the reaction shows a first-order dependence on [substrate], [HBpin], and [**73**-BEt_3_]. Mechanistically, the authors reported that [K][BEt_3_H] **I**, obtained from the reaction of **73** and BEt_3_ with pincacolborane, is the active reducing agent.^138^ On the basis of control experiments and DFT calculations, the authors reported that a plausible mechanism would involve the reaction of **I** with the amide, providing ion-pair intermediate **II**, which subsequently reacts with pinacolborane to afford borane **III** and regenerate active species **I**. Intermediate **III** then provides iminium **IV** species, which in the case of 3° amides is reduced to afford the desired product. On the contrary, with 1° and 2° amines an imine intermediate **V** is obtained *via* intermediate **IV** and HBpin. Finally, imine **V** undergoes reduction, affording the corresponding primary and secondary amines.

**Scheme 45:**
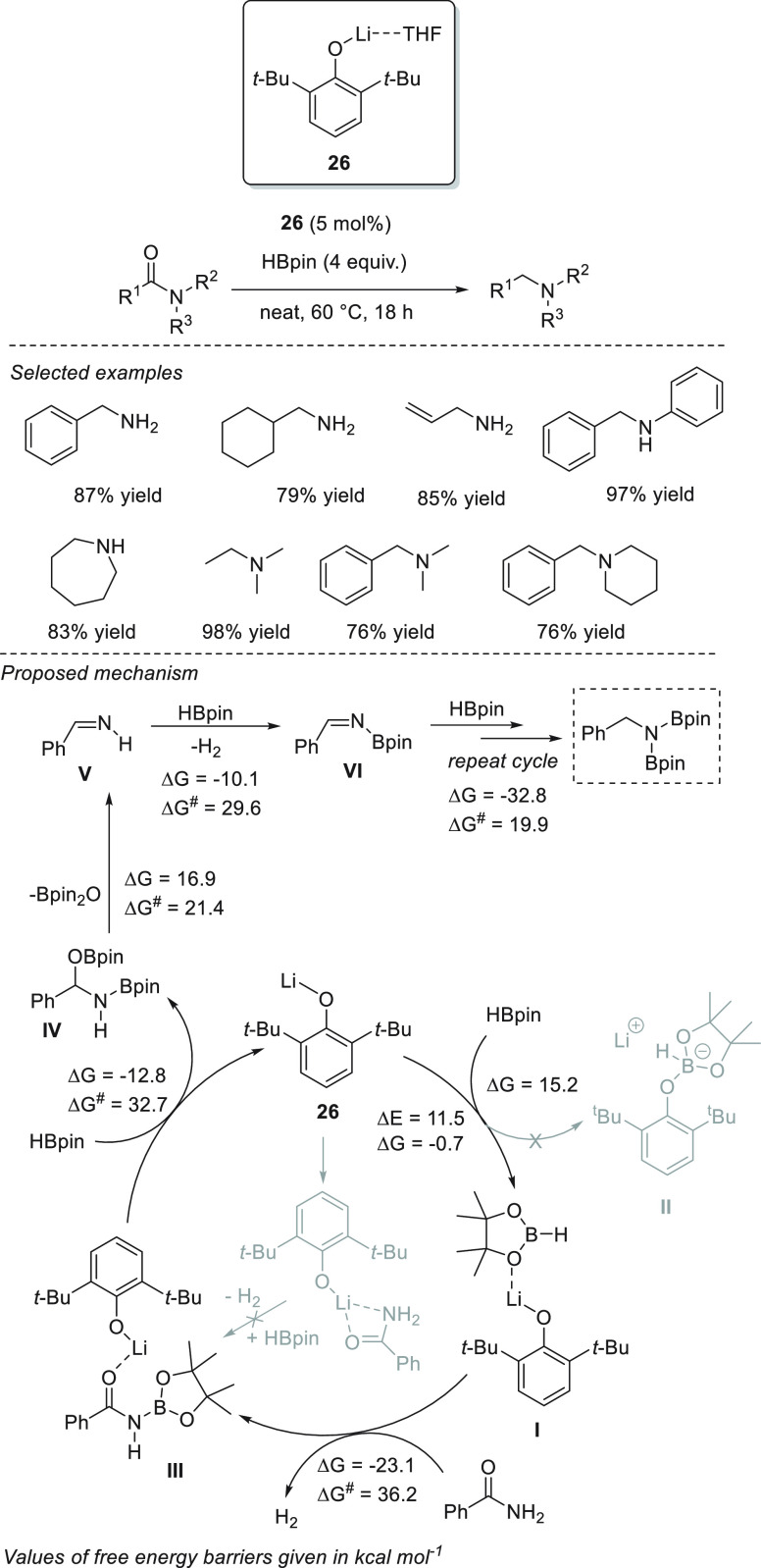
Hydroboration of Amides by Sen *et al.*

Finally, Sen and co-workers reported high efficiency of lithium phenolate **26** as catalyst for the deoxygenative hydroboration of primary, secondary, and tertiary amides (Scheme 45).^139^ In this regard, excellent yields for a wide range of amides could be obtained. In agreement with the previous reports from Sadow,^133^ Mandal,^136^ and Yao,^137^ the authors suggest a pathway *via* the imine intermediate. Thus, lithium **26** coordinates to HBpin to afford intermediate **I**, which after amide insertion, together with hydrogen evolution, gives the *N*-borylated amide **III**. Catalyst **26** coordinates to the C=O bond, favoring the second hydroboration reaction to obtain intermediate **IV**, which undergoes elimination of B_2_pin_2_O and generates imine **V**. Finally, hydrogen evolution and reduction of *N*-borylated imine **VI** provides the corresponding compound. It is important to highlight that direct reactivity of **26** with amide, and the subsequent reaction with HBpin is discarded due to the higher energy barrier required.

Recently, Liu and Cui and co-workers reported the catalytic activity of dimeric magnesium hydride stabilized by phosphinimino **56** toward ester hydroboration, although in comparison with most of the other catalyst, lower activity was observed.^99^

### 3.5 Carbonates and Carbamates

**Scheme 46:**
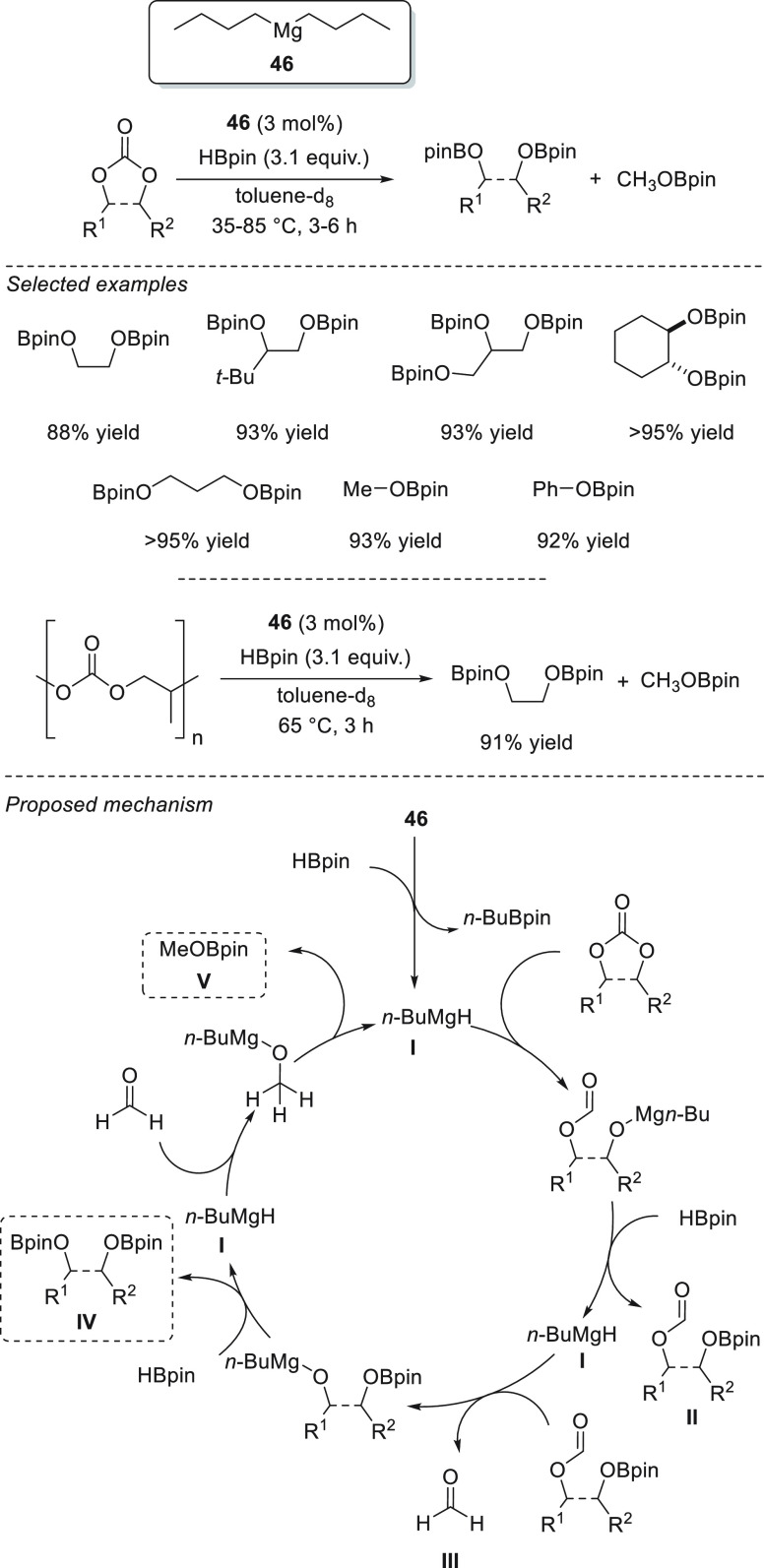
Mg-Catalyzed Hydroboration of Carbonates

The reduction of carbonates leads to methanol and value-added diols or their derivatives. However, carbonates are known to be inert toward reduction due to their high stability.^140^ Thus, the hydroboration of carbonates remains fairly underdeveloped, and the first example was reported recently. Rueping *et al.* applied readily available Mg(*n*-Bu)_2_**46** to the hydroboration of linear and cyclic carbonates.^141^ For the first time, an s-block metal-based catalyst showed activity toward carbonate reduction. This efficient Mg-catalyzed reduction of carbonates provides an efficient indirect route for the conversion of CO_2_ into valuable alcohols (Scheme 46). Moreover, magnesium **46** could also be applied for the depolymerization of polycarbonates. Based on control experiments and NMR spectroscopy, the authors suggest a mechanism for the magnesium-catalyzed hydroboration of carbonates which involves a *n*-BuMgH species obtained by σ-bond metathesis of HBpin and Mg(*n*-Bu)_2_**46**. This active *n*-BuMgH species then participates in three sequential catalytic cycles. After the first hydromagnesiation of carbonate and the subsequent σ-bond metathesis with one molecule of pinacolborane, active *n*-BuMgH **I** is regenerated together with O-Bpin formate **II**. Then species **II** reacts with *n*-BuMgH to provide formaldehyde **III**, and after reaction with a second molecule of pinacolborane, the desired product **IV** is obtained, regenerating *n*-BuMgH **I**, which finally reduces formaldehyde leading to methyl boronic ester **V**. Competition experiments with equimolar amounts of carbonate and formate **II** showed that whereas formate reacted quantitatively, carbonate remained unreacted, thus leading to the conclusion that the reduction of carbonate is the rate-limiting step.

Similarly, Ma *et al.* continued their research on the application of dimeric Mg(I)-based precatalysts for the hydroboration of carbonyl compounds. Just recently, they reported an efficient protocol for the reduction of cyclic and linear carbonates, among other compounds. By performing the reaction under neat conditions, the authors were able to prepare the corresponding Bpin-protected alcohols and diols in the presence of 1 mol % catalyst **78** at ambient temperature.^135^

**Scheme 47:**
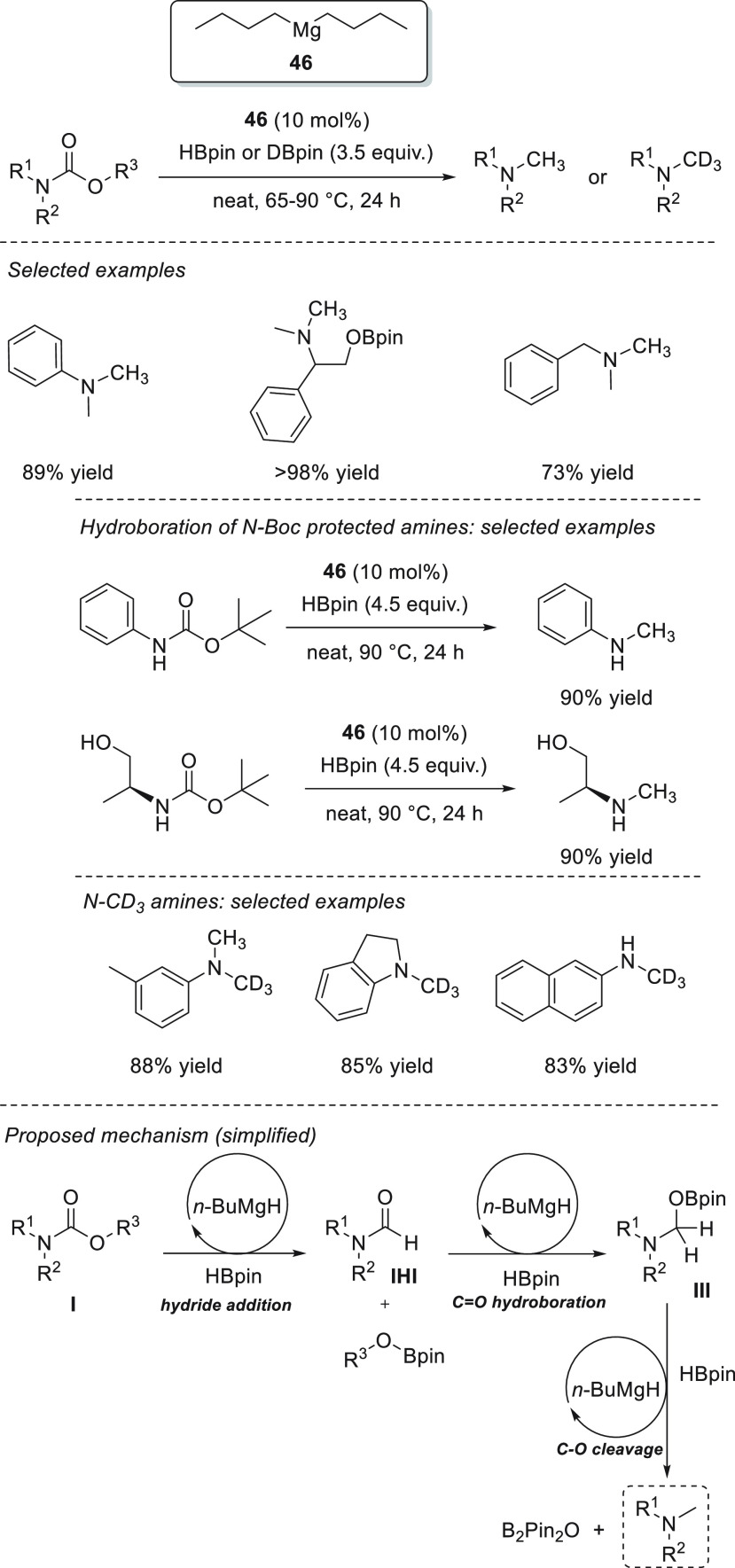
Magnesium-Catalyzed Deoxygenative Reduction of Carbamates

Rueping *et al.* also applied commercially available Mg(*n*-Bu)_2_**46** for the hydroboration of a wide range of secondary and tertiary linear and cyclic carbamates (Scheme 47).^142^ In this case, the hydroboration of methyl and *tert*-butyl carbamates provided the corresponding *N*-methyl amines in excellent yields. It is important to highlight that one of the most applied *N*-protecting groups, the *N*-Boc group, could be used as a C1-building block. Similarly, by using DBpin, the corresponding *N*-trideuteromethyl amines could be obtained. On the basis of the NMR spectroscopy results, the authors suggested a mechanism which involves two sequential reductive steps of the carbamate **I** and the formamide intermediate **II** and a third step that involves a C–O bond cleavage of the obtained *O*-Bpin hemiaminal species **III**. Similar to the hydroboration of carbonates (Scheme 46), the authors established that the rate-limiting step is the reduction of carbonate **I**.

Recently, Liu and Cui reported the catalytic activity of dimeric magnesium hydride stabilized by phosphinimino **56** in the hydroboration of both linear and cyclic carbonates.^99^

### 3.6 Nitriles and Isonitriles

**Scheme 48:**
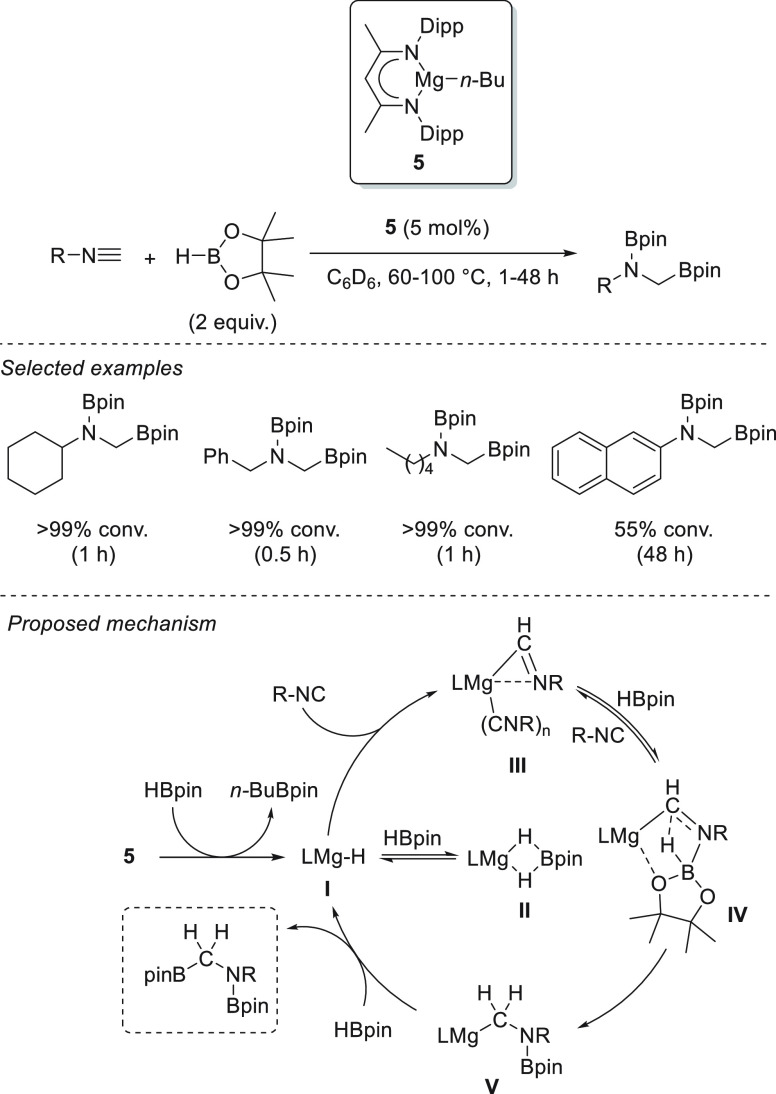
Magnesium-Catalyzed Hydroboration of Isonitriles

The reduction of nitriles and isonitriles, compared to the reduction of other unsaturated compounds, is a fairly underdeveloped field of research, as the only existing example of catalytic hydroboration of this class of compounds was described by Hill *et al.* in 2015.^143^ The reduction of *N*-alkyl-substituted isonitriles to form 1,2-diborylated amines proceeded smoothly in 1 h at a moderate temperature in the presence of β-diketiminato magnesium alkyl complex **5** (Scheme 48). The reduction of *N*-aryl substrates, on the other hand, required much harsher conditions and did not lead to complete consumption of the substrate. The low conversions were attributed not only to the change in electronic properties of the substrates and hence their higher stability but also to the decomposition of the reducing agent at elevated temperature. Mechanistic studies revealed that the first Mg–H addition occurs on the polarized C≡N bond. Then HBpin coordination takes place, leading to an intramolecular hydride transfer from HBpin to the Mg–formimidoyl intermediate. Finally, a σ-bond metathesis of the second molecule of HBpin regenerates the catalyst and provides the corresponding 1,2-diborylated amine products. On the basis of NMR spectroscopy studies, the authors proposed a mechanism in which precatalyst **5** reacts with HBpin to form LMg-H species **I**. After substrate coordination, the first Mg-H addition to the polarized C≡N bond occurs, providing intermediate **III**. Then HBpin coordination takes place, affording species **IV**, which undergoes intramolecular hydride transfer from HBpin to the Mg-formimidoyl intermediate **III** to afford magnesium species **V**. Analysis of the reaction rates indicate a pre-equilibria between **I** and **II** and between **III** and **IV**, which regulates the assembly of a magnesium formimidoylhydroborate **IV**. The intramolecular hydride transfer in **IV** is proposed to be the turnover-limiting step. Finally, a σ-bond metathesis of the second molecule of HBpin regenerates the catalyst and provides the corresponding 1,2-diborylated amine products. It is worth mentioning that the authors did not find enough experimental evidence to rule out the possibility that the formidoyl reduction (intermediate **IV**) and borane metathesis occur in a concerted fashion. Therefore, the product could be obtained through a pathway which would not contemplate intermediate **V**.

**Scheme 49:**
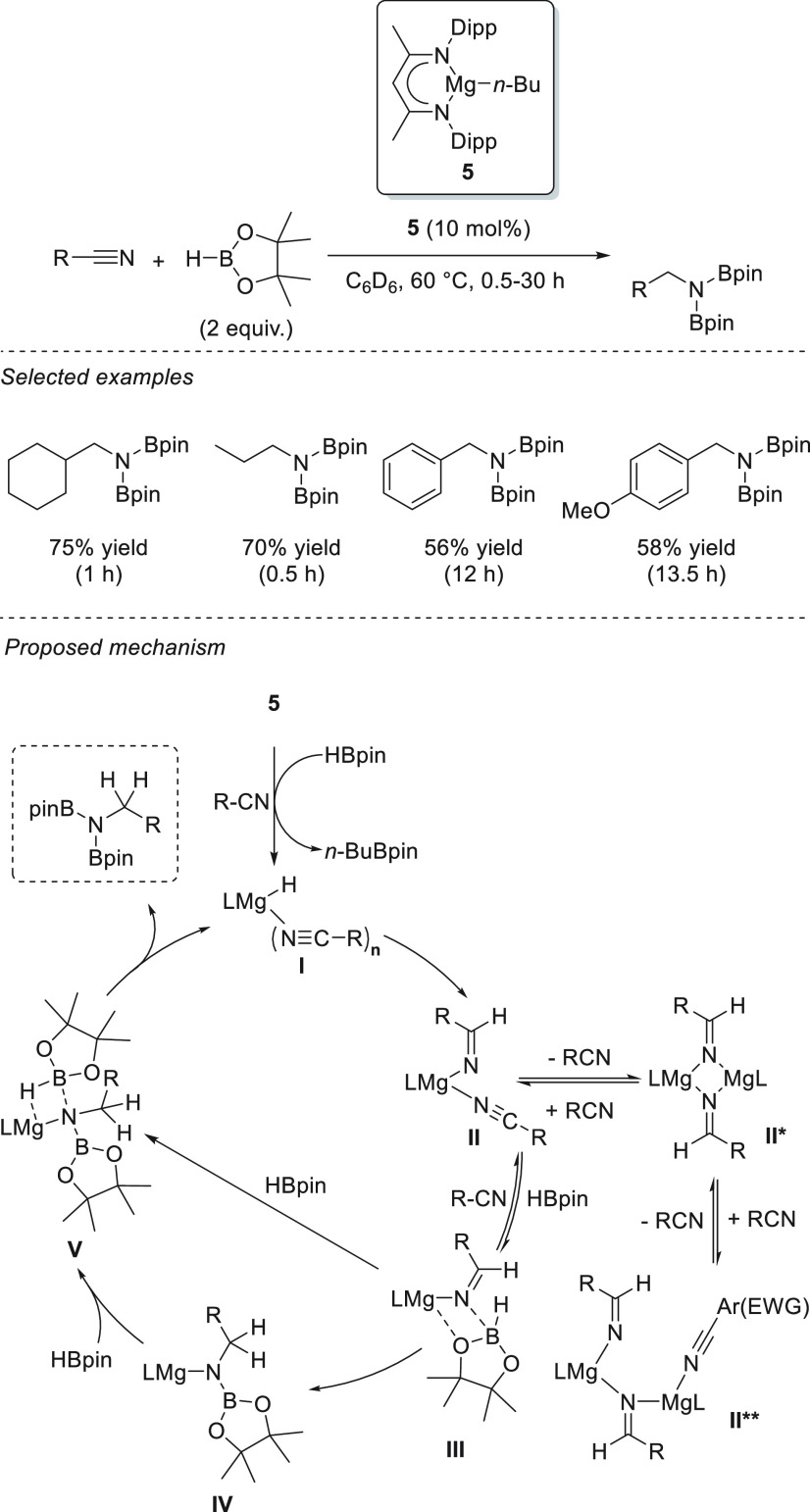
Mechanism for the Dihydroboration of Nitriles Proposed by Hill *et al.*

Hill *et al.* showed that complex **5** is also an active and selective precatalyst for the reductive dihydroboration of organic nitriles and is a useful tool for the synthesis of primary amine derivatives^144^ (Scheme 49). Similar to the reduction of isonitriles, substrates with aliphatic substituents could be reduced in shorter times and under milder reaction conditions compared to aryl nitriles. Mechanistic investigations indicated that the magnesium-catalyzed processes are likely to demonstrate previously unappreciated mechanistic diversity, as follows:

(i) Stoichiometric experiments showed that the reaction proceeds through the generation of magnesium aldimido **II**, magnesium aldimidoborate **III**, and magnesium borylamido **IV** intermediates formed *via* sequential intra- and intermolecular σ-bond metathesis of HBpin.
(ii) Mechanistic differences may depend on the substrate; alkyl nitriles *versus* electron-rich aryl nitriles *versus* electron poor aryl nitriles.
(iii) KIE indicates that B–H bond cleavage and C–H bond formation are involved in the rate-determining process during the dihydroboration of alkyl and electron poor aryl nitriles. With all that information, the authors suggested a common mechanism in which the rate-determining steps vary based on the formation of several pre-equilibria.

Thus, for alkyl nitriles (which exhibit more basic character) the monomer/dimer equilibrium favors the monomeric species **II**. After HBpin coordination, magnesium aldimido hydroborate **III** is obtained. A facile subsequent hydride transfer follows, affording magnesium borylamide **IV**. Finally, Mg–N metathesis with a second equivalent of pinacolborane provides the corresponding bis(boryl) amine product *via* intermediate **V**. The catalytic hydroboration of alkyl nitriles is determined by the pre-equilibria of **II** and **III** and its consumption though a B–H transfer to the coordinated C=N bond. Thus, the observed rate is dictated by not only the ability of HBpin to replace the nitrile substrate but also by the intramolecular C=N reduction. For electron-rich aryl nitriles, the conjugative stability of **III** toward intramolecular reduction would discard the reaction pathway *via* intermediate **IV**. Alternatively, a second molecule of HBpin may coordinate to intermediate **III**, affording intermediate **V**. The second-order [HBpin] is likely the result of two sequential reactions between the magnesium intermediate **III** and HBpin. For electron-deficient aryl nitriles, the Mg–H insertion into RCN provides a dimeric intermediate II*. The apparent independence of [HBpin] and the absence of KIE (using DBpin) suggests that HBpin is not involved in the opening of this dimeric intermediate. Thus, the coordination ability of the substrate to disrupt the dimer II* to form dimer II** with a partial opening may be considered as the rate-determining step. The second order [catalyst] would also agree with an active dinuclear catalysis. Then II** undergoes HBpin coordination and hydride transfer to provide the diborylated amine product.

**Scheme 50:**
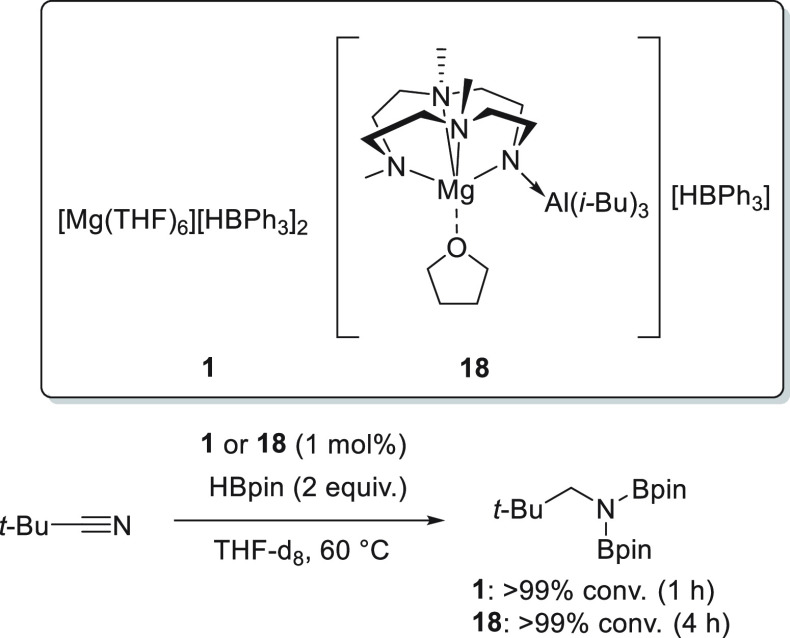
Hydroboration of Nitriles by Okuda *et al.*

Magnesium hydrotriphenylborate complexes **1**(45) and **18**(77) were developed by Okuda *et al.* for the hydroboration of a variety of polarized bonds, including organic nitriles. Although only one substrate has been tested (Scheme 50), the authors proved the high activity of both complexes, as the reduction of *tert*-butyl nitrile was completed in a very short time using 10 times less catalyst than that used by Hill *et al.*(144)

**Scheme 51:**
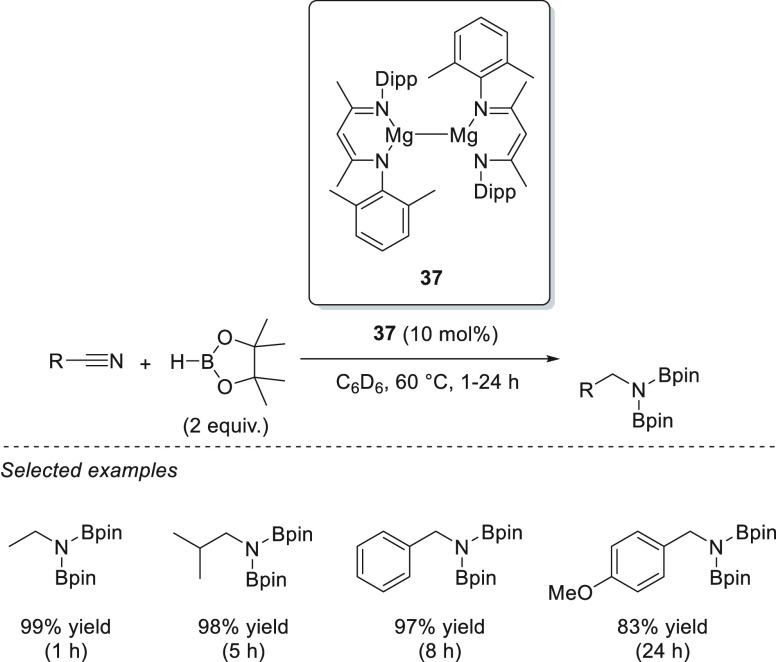
Mg(I) Dimers Developed by Ma *et al.* and Their Application for the Hydroboration of Nitriles

Finally, Ma *et al.* applied an unsymmetrical β-diketiminato-magnesium(I) complex **37** (Scheme 51) for the hydroboration of nitriles.^88^ Although the reaction required high catalyst loadings (10 mol %), the reduction of both aliphatic and aromatic nitriles could be achieved. Notably, the higher activity of complex **37** in the hydroboration of aromatic nitriles than of the initial precatalyst **5** developed by Hill *et al.*(144) was attributed to the better accessibility of the metal center due to the smaller steric hindrance of one of the aryl moieties of the unsymmetrical ligand.

**Scheme 52:**
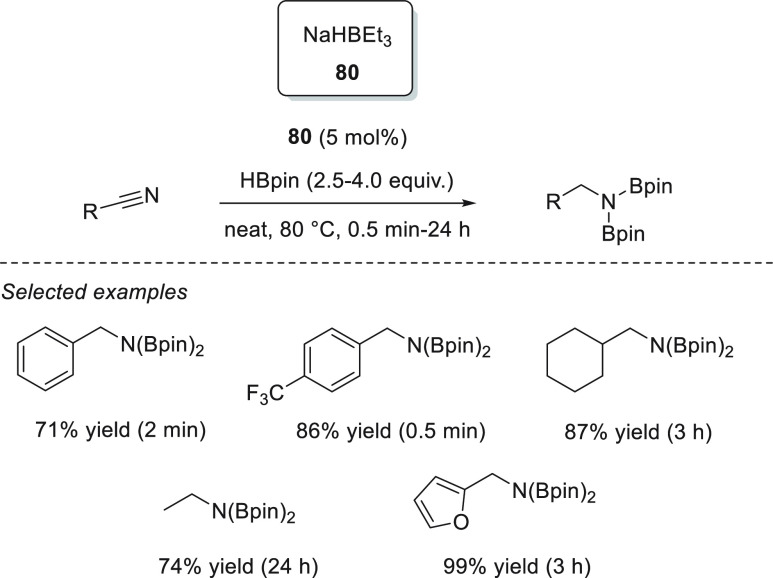
Hydroboration of Nitriles by Findlater *et al.*

Findlater and co-workers showed the high catalytic activity of NaHBEt_3_**80** toward nitrile hydroboration (Scheme 52).^145^ In this regard, excellent yields in short reaction times were obtained for a wide range of aryl and alkyl nitriles, competing favorably with the previous magnesium catalysts reported by Hill,^144^ Okuda,^45,77^ and Ma.^88^

**Scheme 53:**
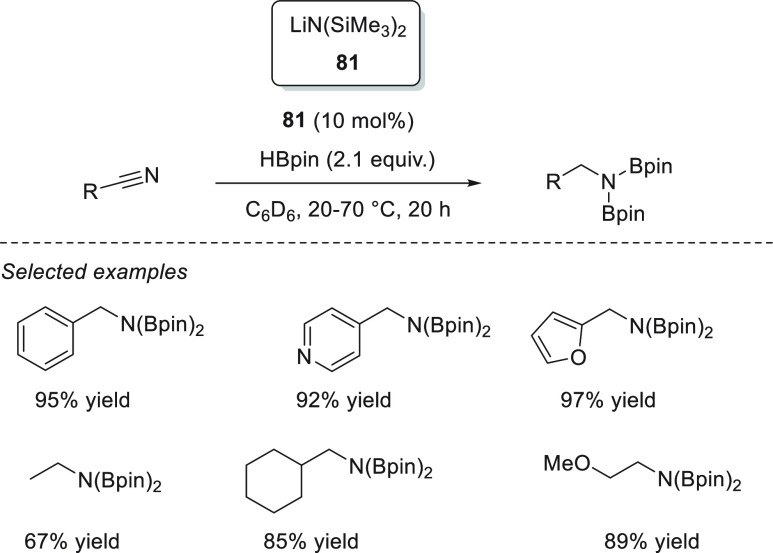
Hydroboration of Nitriles by Wangelin *et al.*

At the same time, Wangelin and co-workers applied lithium amide **81** precatalyst for the hydroboration of nitriles (Scheme 53).^146^ Excellent yields for a wide range of substrates were obtained, although slightly higher catalyst loadings and longer reaction times were required when compared to other previously reported catalysts. Kinetic experiments showed that the reaction exhibited a pseudo-first-order rate dependence on [RCN], first order on [**81**], and zero order on [HBpin]. On the basis of the kinetic and stoichiometric experiments, the authors proposed that upon mixing **81** with the substrate a Lewis acid–base adduct is formed. Then HBpin coordination (*via* O atom to the lithium cation) takes place, followed by an intramolecular hydride transfer, forming lithium–imido compounds that undergo further reduction, affording the corresponding bis-*N*-(borylated) amines.

**Scheme 54:**
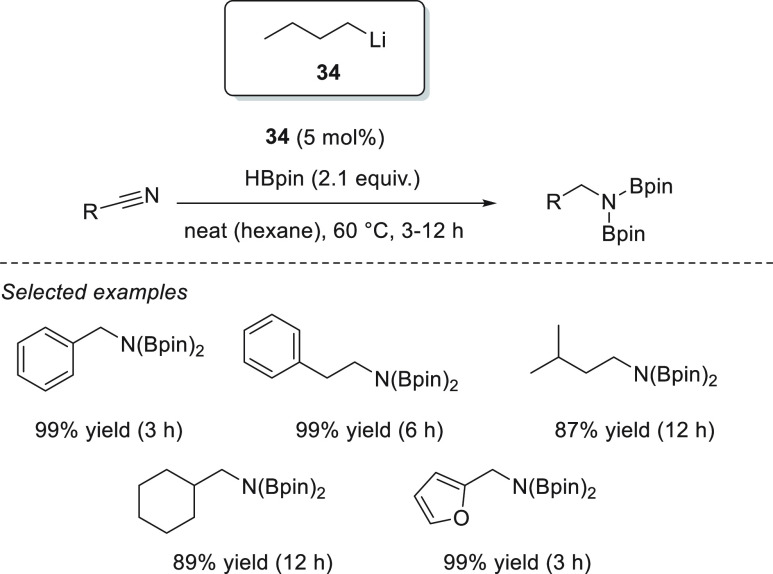
Li-Catalyzed Hydroboration of Nitriles by Yang and Ma *et al.*

Finally, Yang and Ma and co-workers demonstrated the excellent activity of readily available *n*-BuLi **34** as precatalyst in the hydroboration of aryl and alkyl nitriles (Scheme 54).^147^

### 3.7 Isocyanates and Carbodiimides

**Scheme 55:**
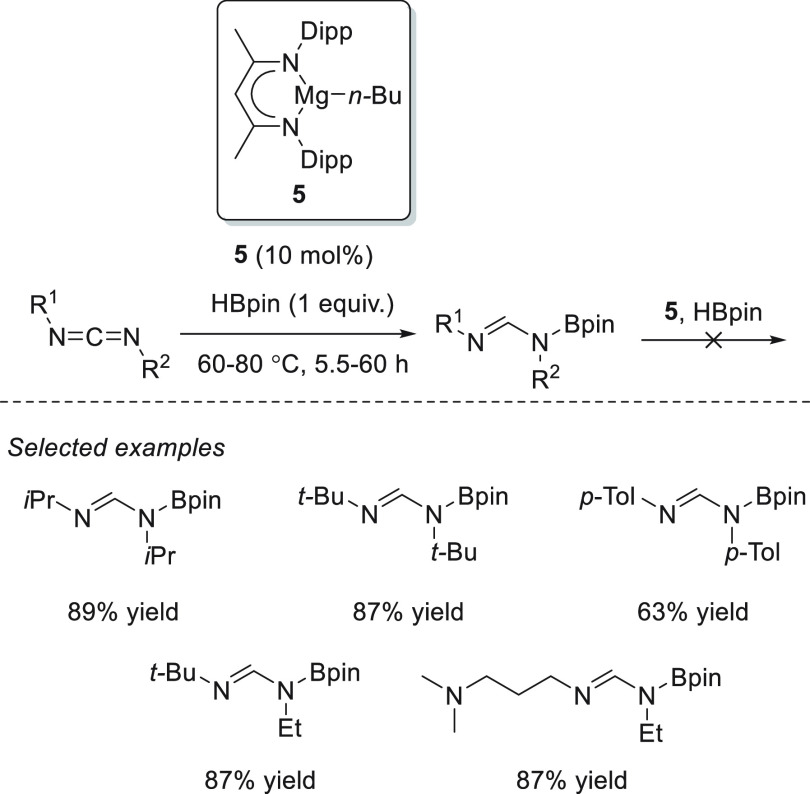
Magnesium-Catalyzed Hydroboration of Carbodiimides

The application of magnesium **5** to the hydroboration of carbodiimides was first presented by Hill *et al.* in 2016.^148^ Interestingly, only partial reduction took place, affording the corresponding *N*-boryl formamidine products. Attempts to induce the second reduction led only to HBpin decomposition. Therefore, a wide range of (*E*)-formamidine derivatives could be obtained under relatively mild reaction conditions (Scheme 55). On the basis of kinetic studies, the authors showed that catalytic turnover is dependent on the cooperative assembly of further carbodiimides and HBpin to affect the formation of the (*E*)-formamidine product.

**Scheme 56:**
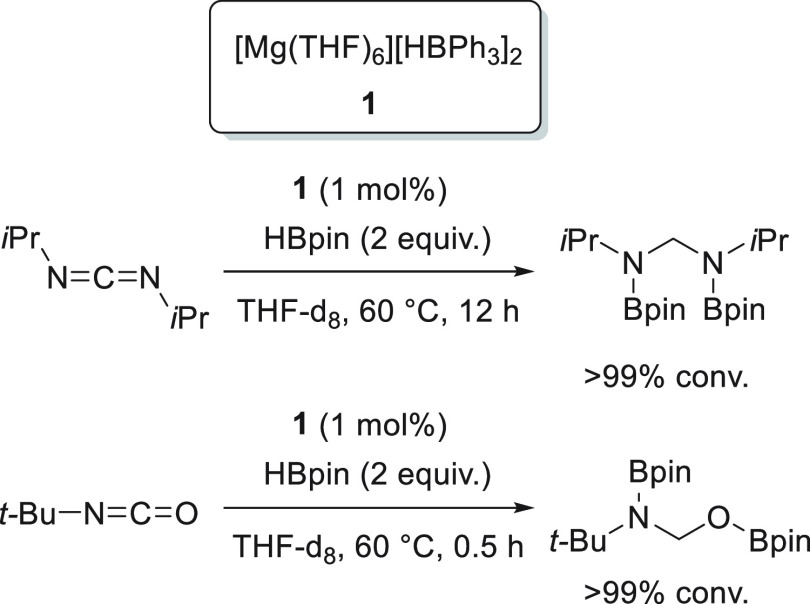
Hydroboration of Isocyanates and Carbodiimides Catalyzed by **1**

In the same year, Okuda *et al.* developed magnesium hydrotriphenylborate complex **1**, which was applied for the hydroboration of polarized bonds, including carbodiimides and isocyanates (Scheme 56).^45^ Although the authors limited the application of the catalytic system to only model substrates, it has been proven that the catalyst exhibits high activity toward the reduction of C=N bonds (cf. Hill’s monohydroboration on Scheme 55), as the dihydroboration of *N*,*N*-diisopropyl carbodiimide and *tert*-butyl isocyanate was complete within 12 and 0.5 h, respectively, in the presence of just 1 mol % catalyst.

**Scheme 57:**
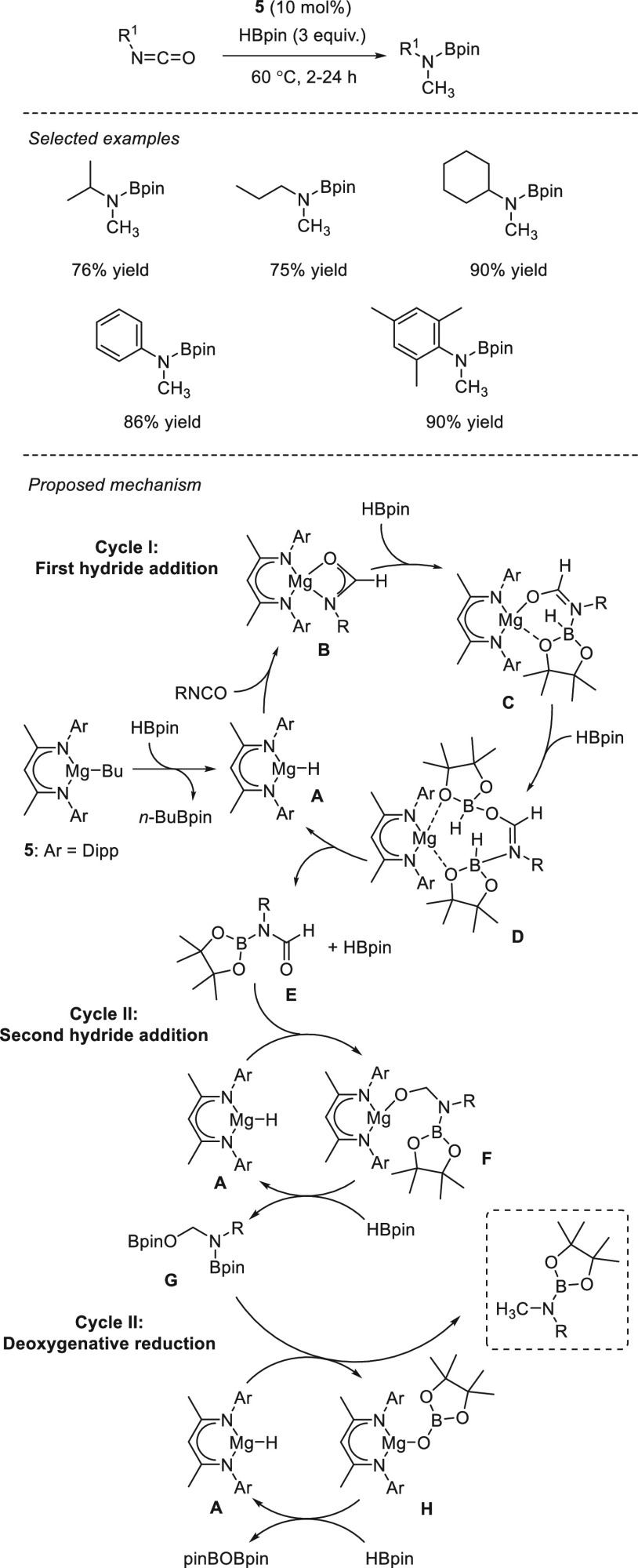
*N*-Methylation of Amines *via* Magnesium-Catalyzed Hydroboration of Isocyanates

Furthermore, Hill *et al.* confirmed the excellent versatility of complex **5** by its application in the reductive hydrodeoxygenation of isocyanates.^149^ Organic isocyanates were easily converted to methyl amines *via* a magnesium-catalyzed hydroboration process (Scheme 57). On the basis of the results of control experiments, NMR spectroscopy, and DFT calculations, the authors suggest that the mechanism involves two hydride additions to isocyanate (cycle I) and formamide intermediate (cycle II) and a third hydride addition that cleaves a C–O bond (cycle III), affording the corresponding *N*-methyl amine. Thus, the authors postulated that precatalyst **5** reacts with pinacolborane to afford the active magnesium hydride **A** which, upon substrate coordination and hydromagnesiation, forms magnesium formamidate species **B**. The reaction with a second equivalent of HBpin leads to intermediate **C**, a borate species formed by the formal insertion of HBpin into the Mg–N bond. In this case, no indication of a Mg–H species was found. Intermediate **C** in the presence of HBpin rapidly affords *N*-borylated formamidine **E***via* magnesium species **D**. The so-obtained *N*-borylated formamidine **E** reacts with **A** to provide diborylated hemiaminal product **G**, presumably *via* magnesium hemiaminal **F**, which was, however, never observed. Finally, the production of *N*-borylated amine product and closure of the catalytic cycle (regeneration of active magnesium hydride **A**) are described to occur *via* a sequential C–O/Mg–H and Mg–O/B–H metathesis steps. DFT calculations showed that this magnesium-mediated C–O bond cleavage is the most energetically demanding catalytic step. After the C–O bond cleavage a magnesium-boryloxide species **H** is formed, which after dimerization and Mg–O/B–H metathesis regenerates the active magnesium hydride species, and (pinB)_2_O is obtained as byproduct. Notably, in the case of the catalyst developed by Okuda *et al.*,^45^ deoxygenative reduction (similar to cycle III) did not take place, and a Bpin–hemiaminal was obtained instead (see Scheme 56).

**Scheme 58:**
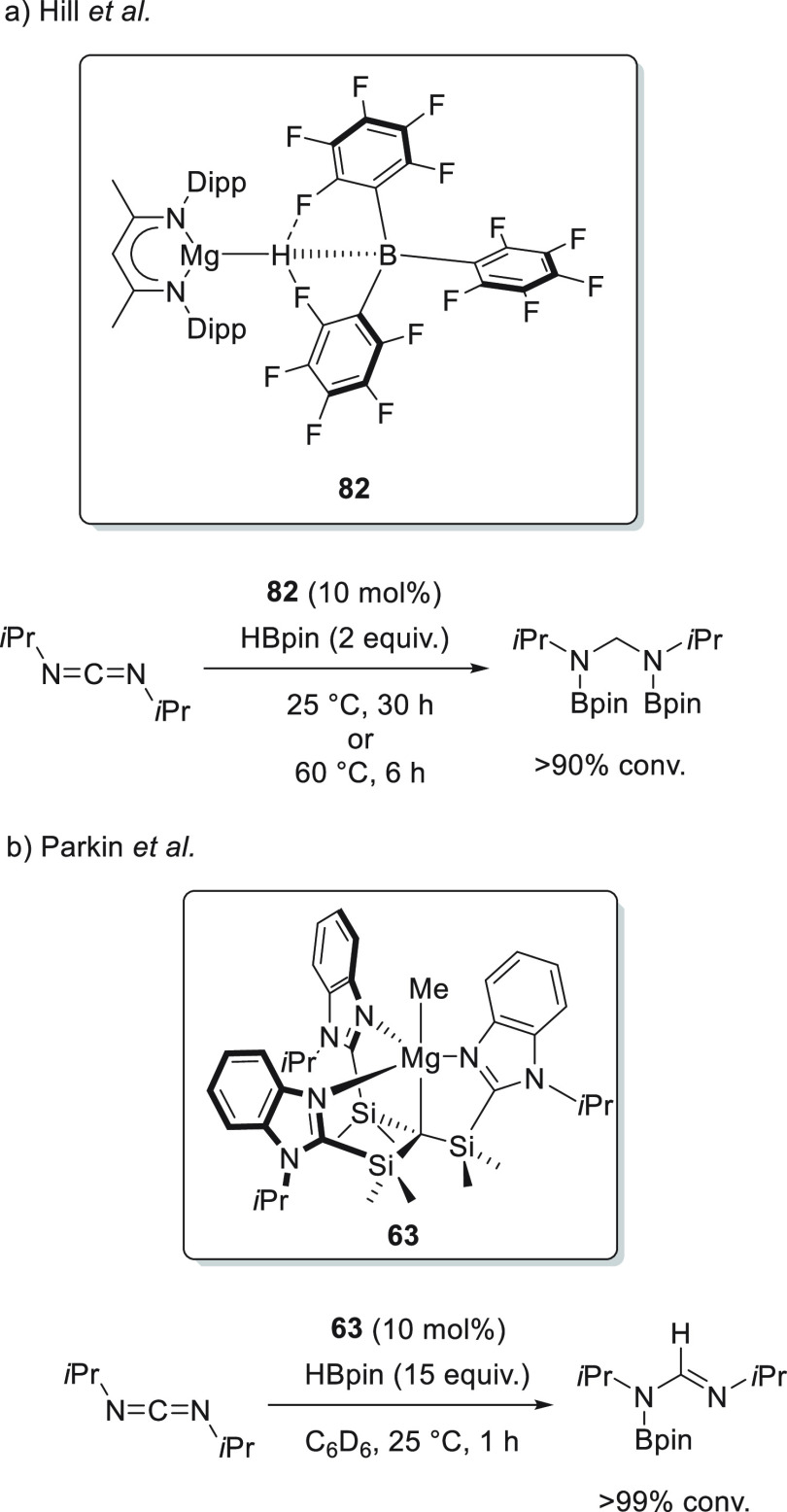
Hydroboration of Carbodiimides Catalyzed by Magnesium Complex **82** and **63**

In 2017, Hill, Mahon *et al.* further expanded the application of magnesium-based complexes for the hydroboration of carbodiimides. Thus, complex **82** was applied for hydroboration of *i*PrN=C=N–*i*Pr with HBpin to afford the corresponding bis(*N*-boryl)aminal (Scheme 58a).^150^ Parkin *et al.* developed [Tism^PriBenz^]MgMe complex **63** (Scheme 58b), and a preliminary test showed that it was able to catalyze the hydroboration of carbodiimides to form *N*-boryl formamidines.^118^ The authors claim that complex **63** shows the highest activity in the hydroboration of carbodiimides among all the magnesium catalysts reported thus far, as the reaction proceeds in a short time at room temperature. It should be pointed out, however, that a large excess of reducing agent was used.

**Scheme 59:**
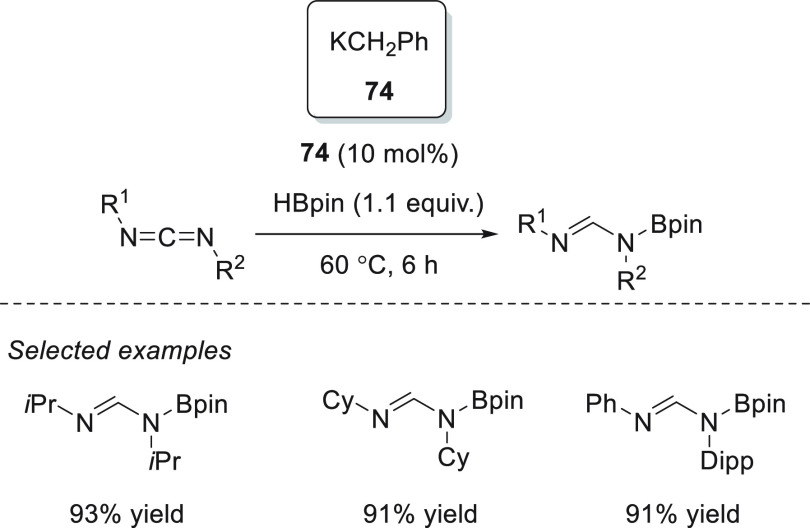
Hydroboration of Carbodiimides Catalyzed by Potassium **74**

Panda *et al.* applied KCH_2_Ph **74** as precatalyst in the hydroboration of carbodiimides as earlier studies showed **74** to be an active precatalyst for the aldimine hydroboration (Scheme 59).^127^ Although higher catalyst loadings were required when compared to magnesium complex **1** developed by Okuda,^45^ precatalyst **74** compares well with magnesium precatalyst **5** applied by Hill *et al.*(148)

Recently, Yang and Ma and co-workers showed that readily available *n*-BuLi **34** can also be used in the hydroboration of dialkyl- and diarylcarbodiimides.^147^ In this regard, precatalyst **34** showed similar activities to magnesium **5** and **1**, previously reported by Hill^148^ and Okuda.^45^

### 3.8 Carbon Dioxide

Hydroboration of carbon dioxide is a convenient approach for conversion of this rather thermally and kinetically stable gas to C1 building blocks.^151^ Although several transition-metal complexes have shown to be active toward carbon dioxide reduction, it was only very recently when alkali- or alkaline-earth-abundant metals were applied.^152^

**Scheme 60:**
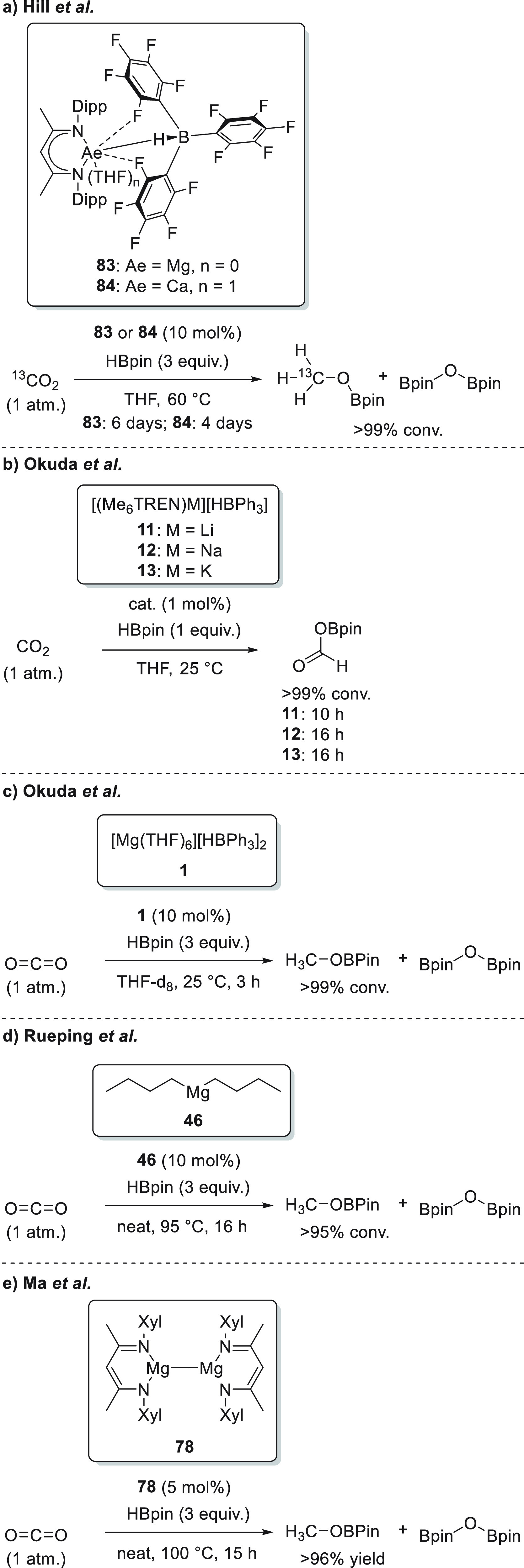
Comparison of Catalytic Activity of Various Alkali and Alkaline Earth Metal Complexes in Hydroboration of Carbon Dioxide

In 2014, Hill *et al.* showed that B(C_6_F_5_)_3_-activated magnesium and calcium hydride complexes **83** and **84** are active for the catalytic hydroboration of CO_2_ (Scheme 60a).^153^ This catalytic system allowed the unprecedented complete and selective reduction of CO_2_ to the methanol equivalent (CH_3_OBpin), although for both catalysts, full conversion was observed only after long reaction times at elevated temperature.

Later, Okuda *et al.* employed a series of alkali metal hydridotriphenylborate complexes **11**–**13** for the selective hydroboration of CO_2_ to primarily reduce formoxyborane (Scheme 60b).^35^ In this case, all complexes promoted hydroboration at very low catalyst loadings following the reactivity trend Li > Na > K, similar to when carbonyl compounds were reduced (section 3.1).

Okuda *et al.* applied magnesium hydrotriphenylborate complex **1** (Scheme 60c) for the hydroboration of carbon dioxide at ambient temperature.^45^ Notably, the complex showed higher activity than magnesium and calcium complexes **83** and **84**.

Commercially available Mg(*n*-Bu)_2_**46** was applied by Rueping *et al.* for the hydroboration of CO_2_ (Scheme 60d). This precatalyst, however, showed much lower activity than previously reported complexes, as harsh reaction conditions and long reaction times were necessary for complete consumption of the substrate.^141^ This observation was also reported by Ma *et al.*, who applied Mg(I) complex **78** for hydroboration of CO_2_ and other carbonyl compounds (Scheme 60e).^135^

### 3.9 Carboxylic Acids

**Scheme 61:**
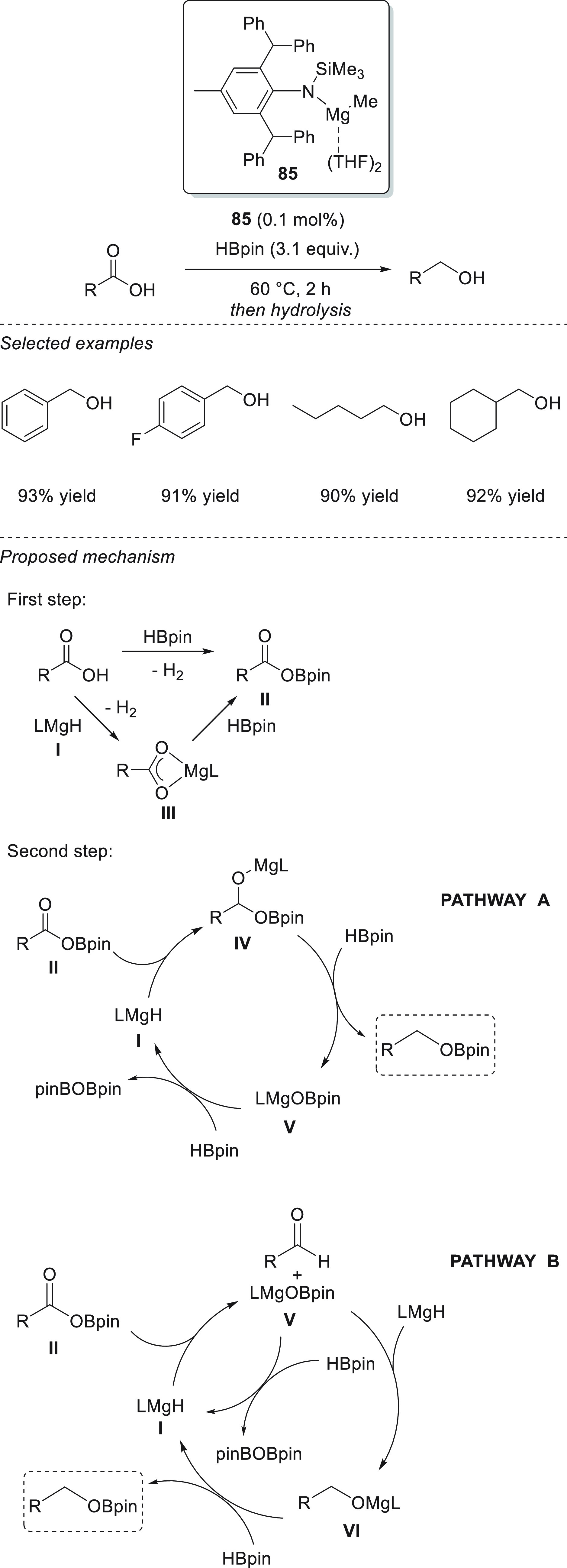
Magnesium-Catalyzed Reduction of Carboxylic Acids

Reduction of carboxylic acids can be performed using stoichiometric amounts of LiAlH_4_. The application of HBpin in combination with metal catalysts is very rare, and the only example of a main group metal catalyzed hydroboration of carboxylic acids was reported by Ma *et al.* in 2020.^154^ Sterically bulky amino magnesium methyl complex **85** was applied for hydroboration of various aliphatic and aromatic carboxylic acids (Scheme 61). Remarkably, the reported catalytic system turned out to be more efficient than the previously reported protocols based on Ru^155^ and Mn.^156^

On the basis of DFT calculations, NMR analysis, and control experiments, the authors proposed a mechanism which involves formation of RCOOBpin **II***via* a noncatalytic reaction of carboxylic acid with HBpin with simultaneous liberation of hydrogen or *via* a Mg-catalyzed pathway. The so-obtained RCOOBpin is then reduced in the presence of in situ formed magnesium hydride **I** (pathway A) to to generate a magnesium complex **V** and eventually the desired product. Alternatively (pathway B), the first step obtained boryl ester could react with LMgH **I** to form an aldehyde and magnesium boryloxide species **V**. The formation of an aldehyde as a plausible intermediate was confirmed *via* NMR experiments. The boryloxide species **V** reacts then with HBpin to regenerate the magnesium hydride with elimination byproduct pinBOBpin. At the same time, the aldehyde is reduced to the corresponding borylated alcohol *via* alkoxide intermediate **VI**. Although the authors reported high activity of their complex, it should be noted that recent studies on catalyst-free hydroboration of carboxylic acids demonstrated that this reaction may in fact proceed efficiently without the presence of any catalyst (see section 7.2).

### 3.10 Sulfoxides

**Scheme 62:**
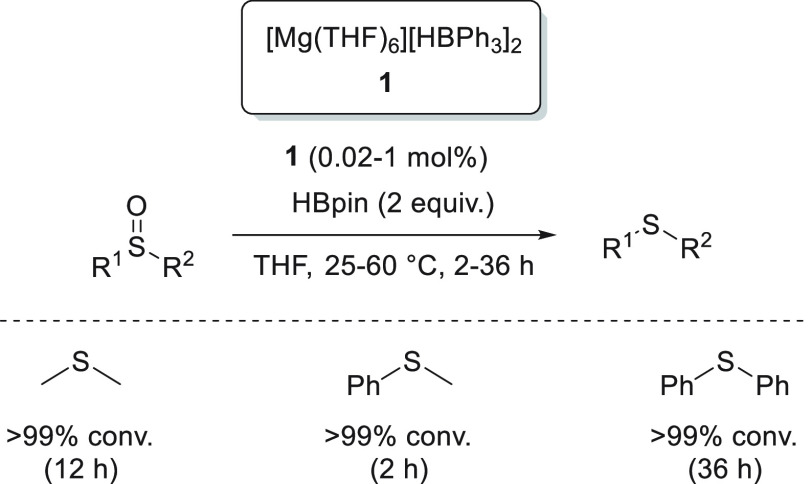
Magnesium-Catalyzed Reduction of Sulfoxides

During studies on the hydroboration of carbonyl compounds in the presence of magnesium hydrotriphenylborate complex **1**, Okuda *et al.* found that when the reactions were carried out in DMSO, catalytic deoxygenation of DMSO occurred as a side reaction (Section 3.1). The authors further investigated the reactivity of complex **1** for the hydroboration of sulfoxide, and thus, the only existing protocol of such a reaction in the presence of an alkaline earth metal-based catalyst was reported (Scheme 62).^45^ Catalytic deoxygenation proceeded even under mild reaction conditions and low catalyst loadings; however, longer reaction times were required, and the substrate scope was limited to only three examples.

## 4 Hydroboration of Unsaturated C–C Bonds

### 4.1 Alkenes

**Figure 9:**
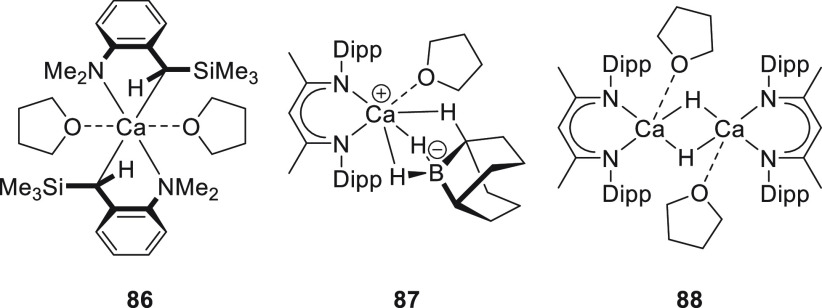
Calcium complexes applied in the hydroboration of unsaturated bonds.

Although the transition-metal-catalyzed hydroboration of alkenes has been widely studied,^157−160^ studies of the hydroboration of alkenes catalyzed by s-block metals have been limited.^161^ The first example of the s-block metal-catalyzed hydroboration of unsaturated C–C bonds was reported by Harder *et al.* in 2012. Calcium-based complexes **86**–**88** (Figure 9) were investigated as potential catalysts in the hydroboration of 1,1-diphenylethylene using HBcat (catecholborane) as reducing agent. Surprisingly, the product of the reaction was not the expected Ph_2_CHCH_2_Bcat; instead, (Ph_2_CHCH_2_)_3_B was formed. By means of NMR spectroscopy, the authors proved that organocalcium complexes **86**–**88** decompose HBcat to BH_3_ or B_2_H_6_, which are the actual active species in the reaction. By using less reactive HBpin, they found that the organocalcium complexes decompose even at room temperature.^162^

**Scheme 63:**
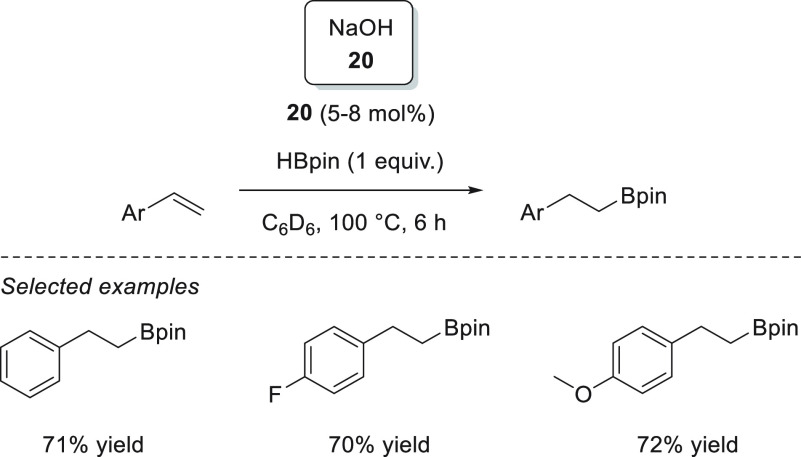
Na-Catalyzed Hydroboration of Alkenes

Several years later, Wu, Liu, and Zhao *et al.* showed that a catalytic hydroboration of nonpolarized unsaturated compounds, such as alkenes, could be carried out in the presence of NaOH **20** as a precatalyst (Scheme 63).^79^ The authors postulated that the reaction is possible due to the formation of an anionic borodihydride species from the reaction of borane and NaOH, which then acts as a catalyst (see Scheme 15).

**Scheme 64:**
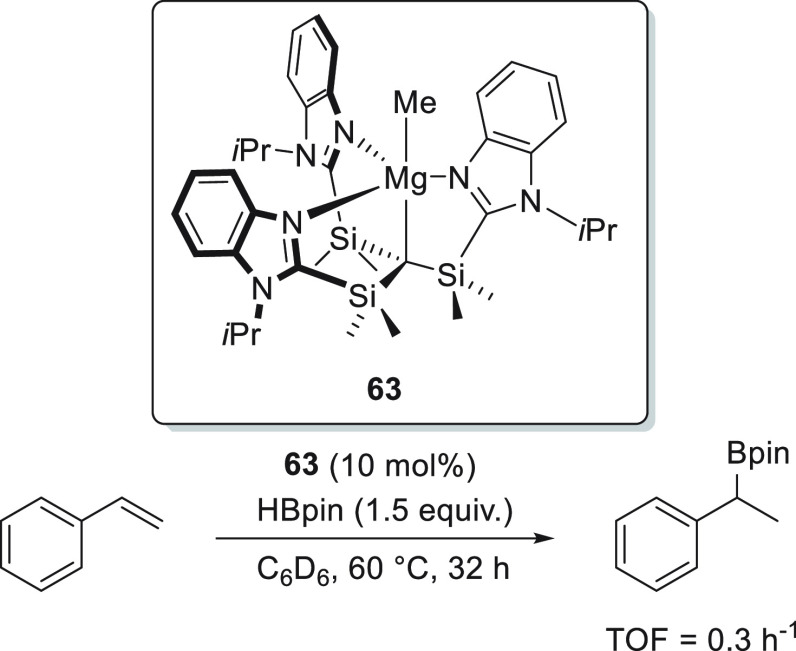
Magnesium-Catalyzed Hydroboration of Styrene

The only example of magnesium-catalyzed hydroboration of alkenes reported thus far was reported in 2017 by Parkin *et al.*, who applied magnesium complex **63** for the hydroboration of styrene (Scheme 64).^118^ Remarkably, in contrast to the *anti*-Markovnikov regioselectivity observed when transition-metal catalysts were used, the hydroboration in the presence of **63** as a precatalyst proceeded in a Markovnikov manner. Although only styrene was tested, this was the first example of such a transformation in the presence of a magnesium catalyst.

**Scheme 65:**
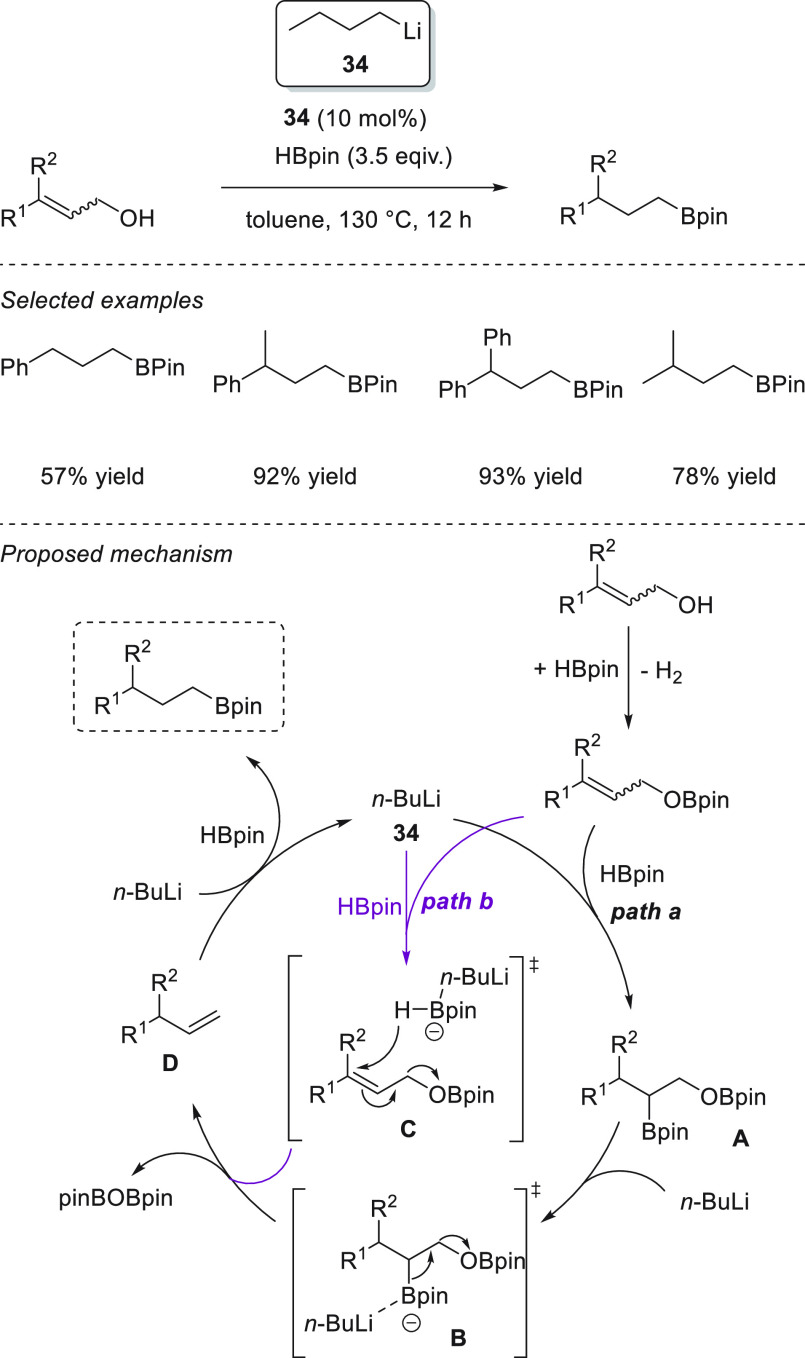
Li-Catalyzed Reductive Relay Hydroboration of Allylic Alcohols

In 2019, Xu and Shi *et al.* showed that *n*-BuLi **34** may also be active for the reductive relay hydroboration of allylic alcohols (Scheme 65).^163^

Mechanistic studies revealed that this process involves a one-pot three-step process involving:

(i) Pathway a: base-promoted regioselective hydroboration of the allylic intermediate **A** (obtained *via* dehydrocoupling of allylic alcohol with pinacolborane), followed by β-oxygen elimination (transition state **B**, path a), to afford alkene **D**.
(ii) Alternative pathway b: allylic hydride substitution of allylic intermediate **A** mediated by an in situ generated borohydride (transition state **C**) to afford alkene **D**.
(iii) Finally, an *anti*-Markovnikov hydroboration step of **D** to afford the desired product.

Although the authors report that *n*-BuLi **34** species are regenerated within the proposed catalytic cycle (Scheme 65), no experimental evidence is reported. Because of the high reaction temperatures (130 °C) and the presence of allylic alcohol as substrates, alternative regenerated Li species should be considered.

**Scheme 66:**
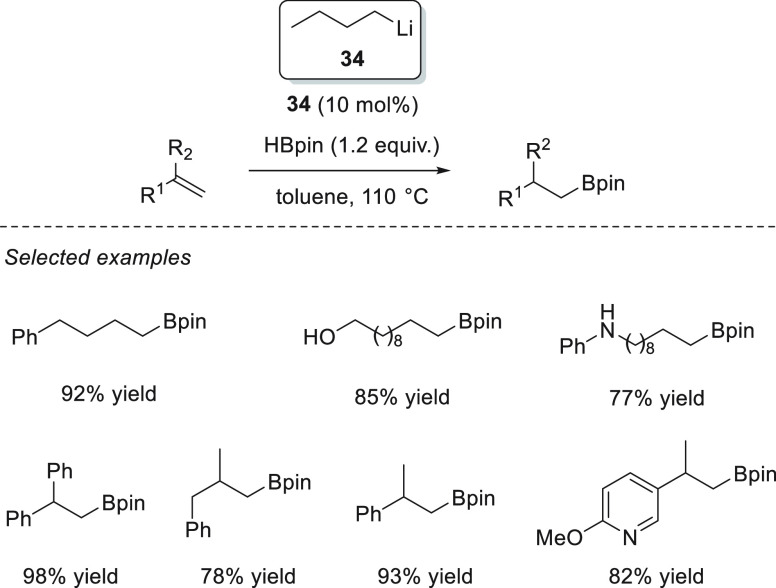
Li-Catalyzed Hydroboration of Alkenes

The same authors reported an efficient and general *n*-BuLi-promoted *anti*-Markovnikov selective hydroboration of various terminal α- and 1,1-disubstituted alkenes, providing the corresponding alkyl boronic esters bearing various functional groups as single regioisomers with very good yields (Scheme 66).^164^

Similarly, Sen *et al.* reported that lithium complexes **26** and **27**, which were previously employed for the hydroboration of polarized unsaturated compounds such as ketones and aldehydes (Scheme 17),^82^ could also be applied for the hydroboration of alkenes.^165^ Interestingly, when 2-substituted 1,3-diene was used in the reaction, the 3,4-selective hydroboration product was obtained exclusively. Finally, An *et al.* discovered that potassium carbonate **45** was an active precatalyst for the hydroboration of a wide range of terminal alkenes.^92^ This method employing inexpensive, readily available, and air-stable potassium salts afforded products with moderate to very good yields.

### 4.2 Alkynes

**Scheme 67:**
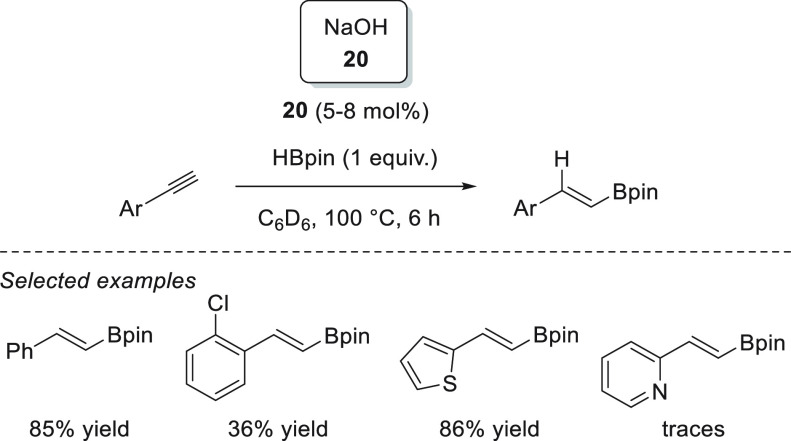
Na-Catalyzed Hydroboration of Alkynes

Vinylboranes are versatile precursors that have been widely used in organic synthesis, for instance, in the Suzuki–Miyaura reaction. Although in recent years the catalytic hydroboration of alkynes has increasingly gained attention,^166−169^ the application of main group metal catalyst is at its early career stage.^170^ Wu, Liu, and Zhao *et al.* showed that catalytic hydroboration alkynes could be initiated by simple sodium hydroxide **20** (Scheme 67).^79^ The desired products were isolated, in most cases, in moderate to good yields with *anti*-Markovnikov regioselectivity and (*E*)-stereoselectivity; however, the authors limited the substrate scope to only aryl-terminal alkynes.

One year later, Ma *et al.* applied unsymmetrical β-diketiminate magnesium(I) complex **37** for the hydroboration of terminal alkynes.^88^ Although harsh reaction conditions were required, excellent yields of aryl- and alkyl-terminal alkynes were obtained. Only one example of an internal alkyne was reported, although with moderate regioselectivity.

**Scheme 68:**
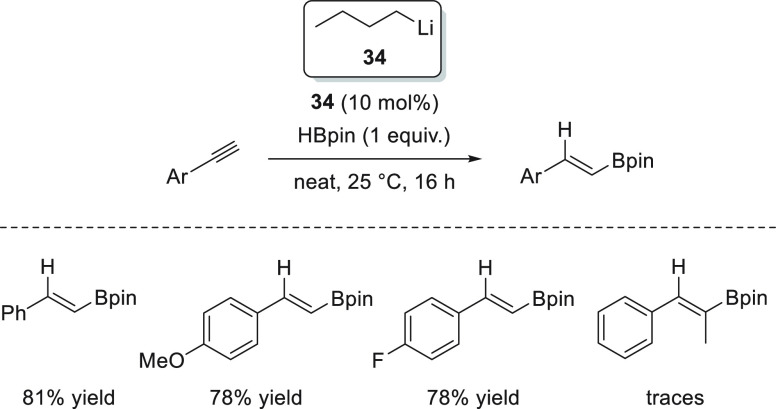
Li-Catalyzed Hydroboration of Alkynes

Xue and Bao *et al.* extended the application of *n*-BuLi **34** as a precatalyst for the hydroboration of alkynes under mild reaction conditions (Scheme 68).^126^ Although the authors described the reaction conditions as “neat”, some solvent from *n*-BuLi stock solution has been involved. For the first time, a lithium catalyst was shown to be active toward the hydroboration of nonpolarized unsaturated bonds; however, relatively high catalyst loading was necessary. Unfortunately, this catalytic system failed when internal alkynes were tested.

**Scheme 69:**
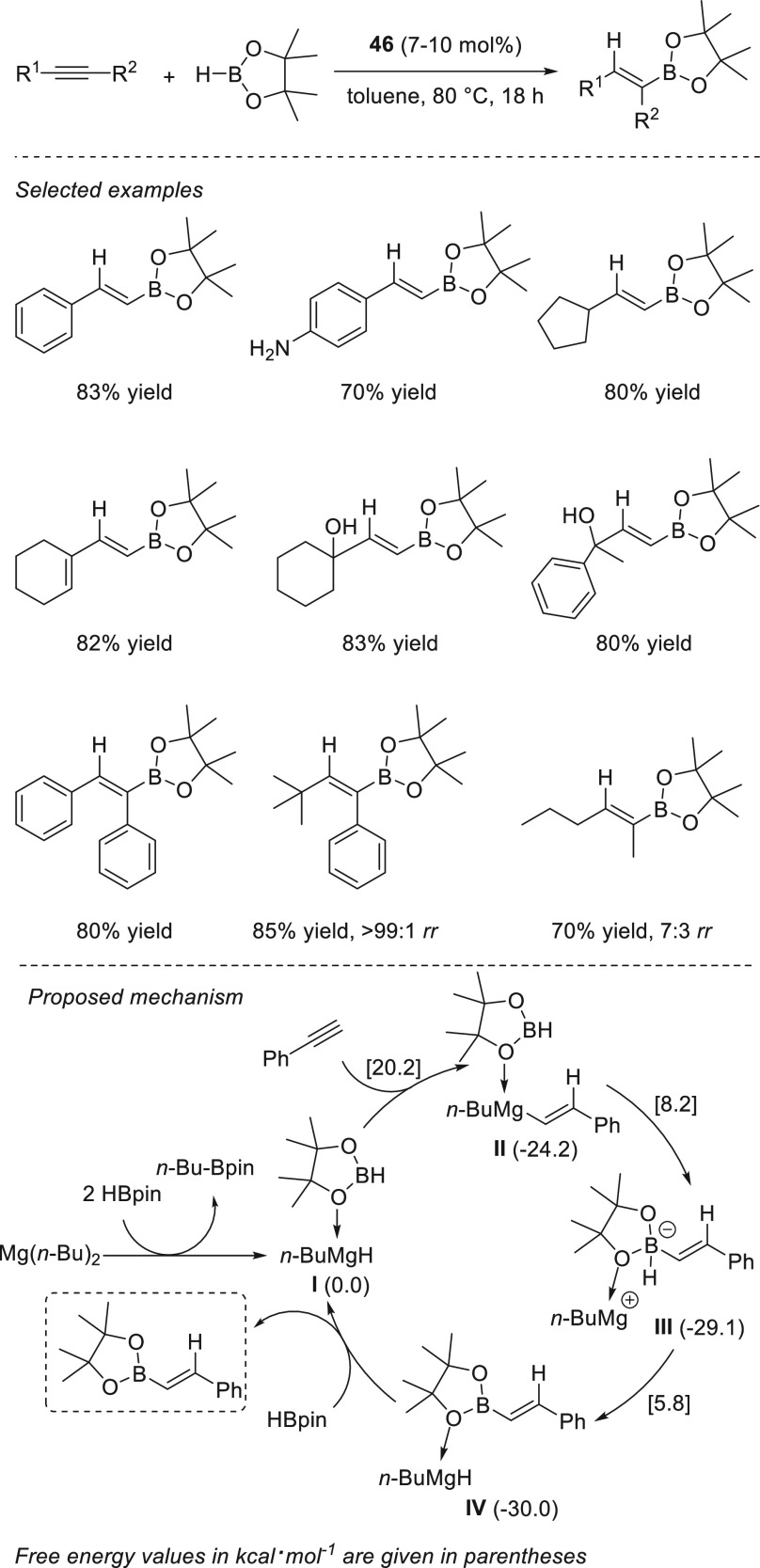
Mg-Catalyzed Hydroboration of Terminal and Internal Alkynes

Rueping *et al.* applied commercially available Mg(*n*-Bu)_2_**46** for the hydroboration of a wide range of terminal and internal alkynes.^171^ Low-cost and readily available magnesium species **46** provided the corresponding (*E*)-vinyl boranes in excellent yields (Scheme 69).

Moreover, precatalyst **46** showed excellent functional group tolerance, and the hydroboration of alkynes bearing hydroxyl and free amino groups proceeded with excellent yields and with *syn*-stereoselectivities. It is important to highlight the good to excellent regioselectivities obtained for a wide range of internal unsymmetrical alkynes. The authors provided insight into the reaction mechanism, and based on the results of NMR spectroscopy and DFT calculations, they proposed the catalytic cycle, based on catalytically active magnesium hydride species, which involves:

(i) *The formation of active n-BuMgH species*: By means of NMR spectroscopy and DFT calculations, the authors suggested that mixing commercially available **46** with pinacolborane, *n*-BuMgH **I**, and *n*-BuBpin were formed *via* a σ-bond metathesis pathway (8.9 kcal mol^–1^).
(ii) *Alkyne hydromagnesiation step:* In situ formed magnesium hydride species **I**, which contains one molecule of coordinated HBpin, undergoes hydrometalation of alkyne (20.2 kcal mol^–1^) to afford the corresponding vinyl magnesium **II**.
(iii) *Nucleophilic migration*: The next step is the nucleophilic migration of the vinyl group to the boron atom of the coordinated pinacolborane (8.2 kcal mol^–1^), forming a zwitterionic intermediate **III**, which is more stable than vinyl magnesium **II** species.
(iv) *Hydride migration:* Finally, a reverse hydride migration of anionic borohydride to magnesium center (5.8 kcal mol^–1^) provides the corresponding (*E*)-vinyl borane and regenerates the active *n*-BuMgH **I** species.

The significantly lower energy barriers of nucleophilic migration and hydride migration steps (8.2 and 5.9 kcal mol^–1^, respectively) suggest that the rate-limiting step in the catalytic cycle is the hydromagnesiation step (20.2 kcal mol^–1^).

**Scheme 70:**
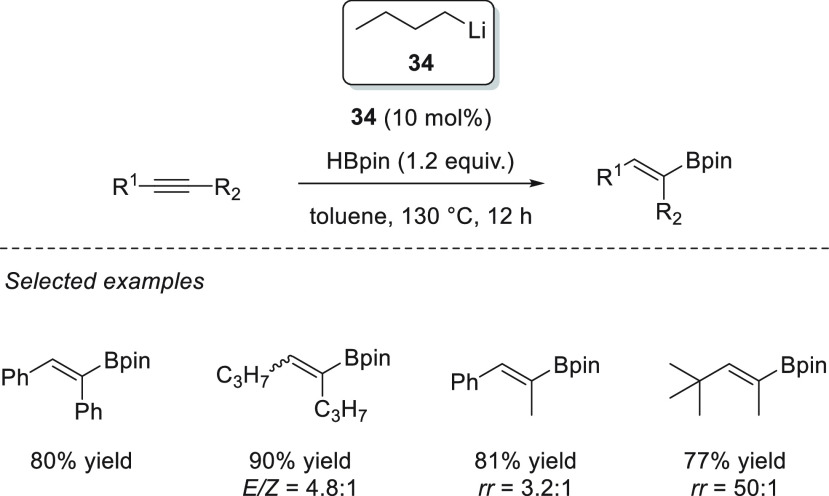
Li-Catalyzed Hydroboration of Alkenes and Alkynes

Xu and Shi *et al.* reported an efficient and general *n*-BuLi-promoted *anti*-Markovnikov selective hydroboration of various terminal and internal alkynes (Scheme 70).^164^ When nonsymmetrical internal alkynes were tested, moderate to excellent regioselectivities were observed. Moreover, harsher reaction conditions than those reported by Xue and Bao *et al.*(126) were required to make the hydroboration of internal alkynes possible.

At the same time, Sen *et al.* reported that lithium complexes **23** and **24**, which were active for the hydroboration of polarized unsaturated bonds such as ketones and aldehydes,^82^ were also active toward alkyne hydroboration.^165^ Smooth hydroboration of different aromatic terminal alkynes with electron-donating or electron-withdrawing substituents at the *o*/*m*/*p*-positions was reported, providing the corresponding products with very good conversions. On the other hand, when internal alkynes were tested, only moderate yields and stereoselectivities were achieved.

## 5 Hydroboration of Strained Systems: Epoxides and Oxetanes

The ring opening of strained systems such as epoxides and oxetanes is a powerful tool to obtain alcohols. In this regard, the ring opening of nonsymmetrical substrates afford mixtures of regioisomers; traditionally dependent on the reducing agent employed.^172^ Generally, the catalytic C–O bond cleavage is fairly limited due to the high stability of the metal–alkoxide products, which hampers the regeneration of the metal hydride intermediate.

In the recent years, the transition-metal-catalyzed hydroboration of epoxides has emerged as a good method for the synthesis of alcohols. The use of mild pinacolborane as a reducing agent resulted in good-to-excellent selectivities, however, exclusively toward linear alcohols.^172^

**Scheme 71:**
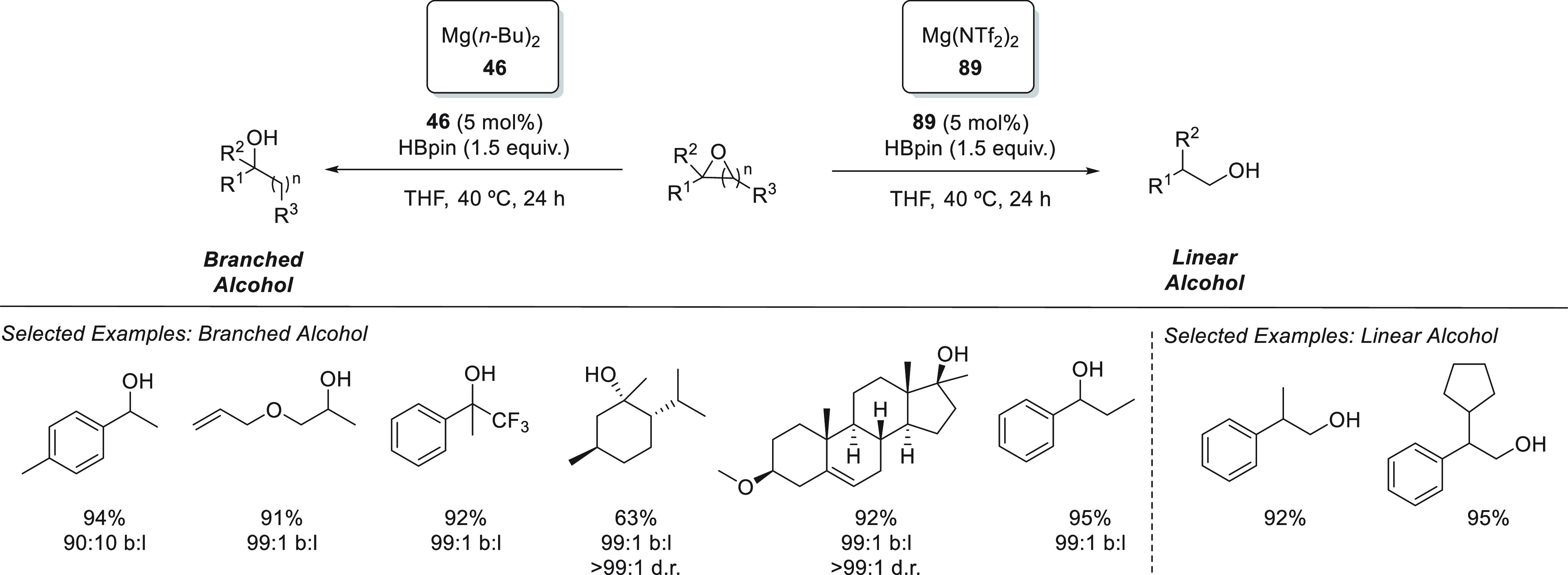
Magnesium-Catalyzed Regiodivergent Hydroboration of Epoxides and Oxetanes

In 2020, Rueping *et al.* reported the first main group metal catalyzed hydroboration of epoxides and oxetanes to obtain the corresponding alcohols.^173^ The authors demonstrated that readily available Mg(*n*-Bu)_2_**46** was able to catalyze the ring opening of terminal and internal epoxides and oxetanes to afford the corresponding branched alcohol, the opposite regioselectivity compared to transition-metal-catalyzed hydroboration of epoxides.^174^ In addition, enantiopure tertiary alcohols were also obtained as a result of the enantiospecific ring opening of optically pure epoxides and epoxides derived from natural products, showing excellent functional group tolerance (Scheme 71). Interestingly, the good performance of **46** could be also extended to the hydroboration of less reactive oxetanes which had not even been reported with transition-metal catalysts. In addition, the authors found that replacing the Mg(*n*-Bu)_2_**46** precatalyst with readily available Mg(NTf_2_)_2_**81** completely reversed the regioselectivity. In this regard, magnesium catalyst **81** provided the corresponding linear alcohol in excellent yields and regioselectivities for a wide range of terminal epoxides.

**Scheme 72:**
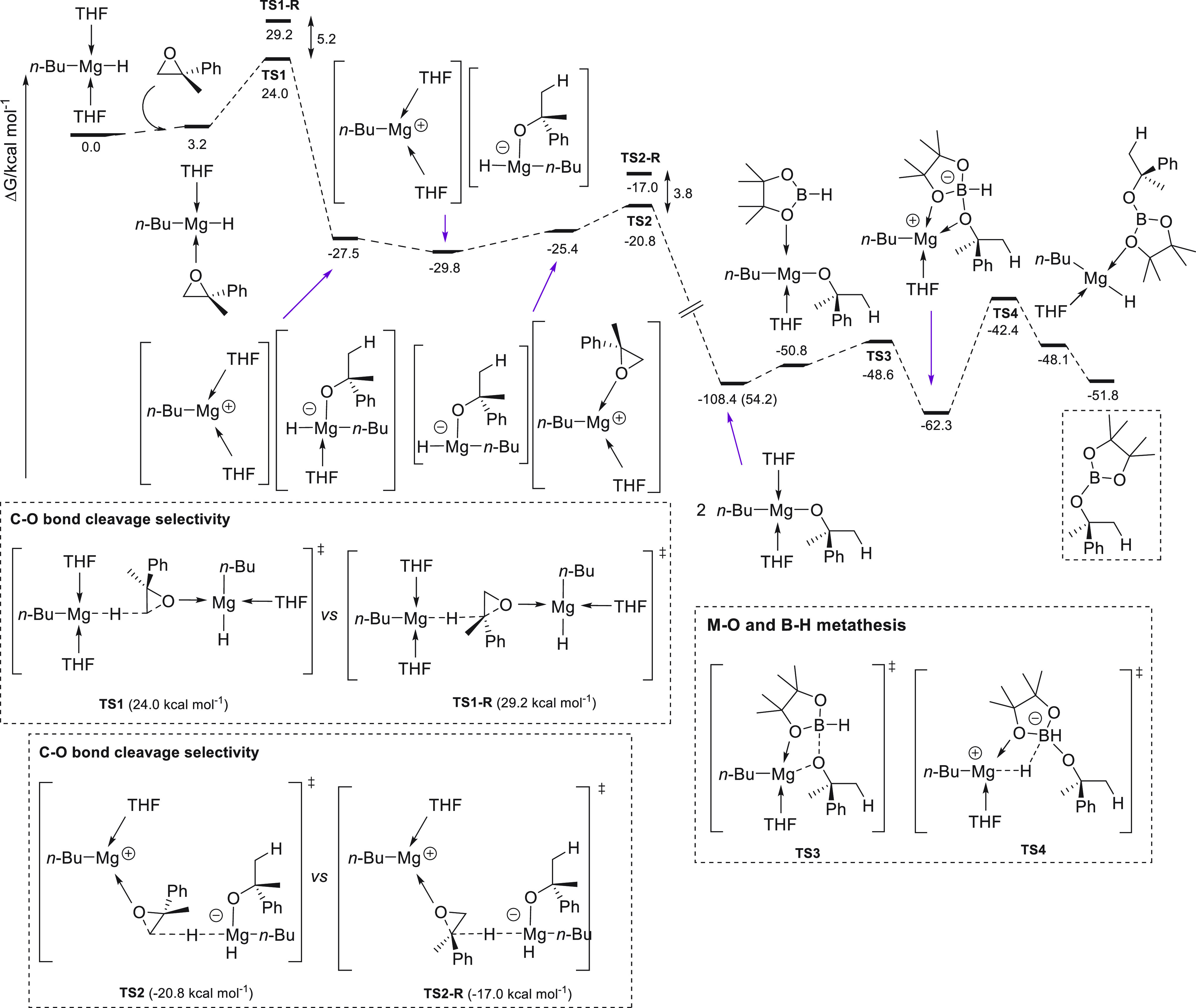
Mechanism of Mg-Catalyzed Ring Opening of Epoxides Using Mg(*n*-Bu)_2_

**Scheme 73:**
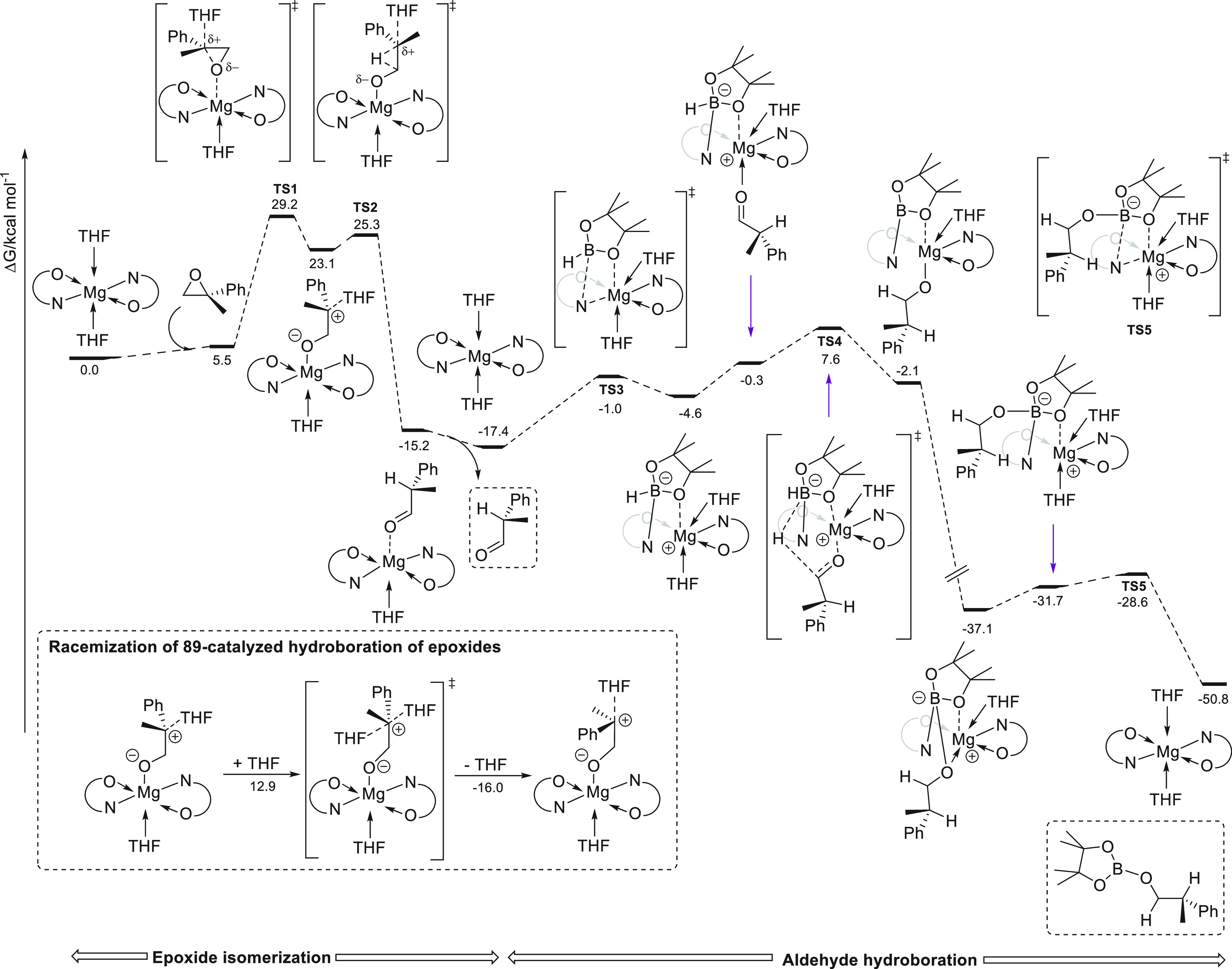
Mechanism of Mg(NTf_2_)_2_-Catalyzed Ring Opening of Epoxides

Mechanistically, based on control experiments and DFT calculations, the authors elucidated two different mechanisms for magnesium **46** and magnesium **89** catalyzed reactions (Scheme 72 and Scheme 73 respectively).

For the Mg(*n*-Bu)_2_**46**-catalyzed procedure (Scheme 72), the authors suggest that after epoxide coordination to active *n*-BuMgH species a bimolecular ring-opening mechanism occurs in which epoxide activation and hydride addition to the least substituted carbon take place simultaneously (5.2 kcal mol^–1^ difference between **TS1** and **TS1-R**) to provide the corresponding magnesium alkoxide intermediate. Then HBpin activation occurs, followed by alkoxide migration to pinacolborane (**TS3**), and the resulting zwitterionic species undergoes hydride transfer (**TS4**) to liberate the branched pinacol ester product with the regeneration of active *n*-BuMgH. Thus, the overall reaction profile (Scheme 72) shows that the bimetallic hydride transfer *via***TS1** is the rate-controlling step. Regarding the regioselectivity, as mentioned before, differences in energy of 5.2 kcal mol^–1^ (**TS1***vs***TS1-R**) and 3.8 kcal mol^–1^ (**TS2***vs***TS2-R**) are consistent with the high regioselectivity observed.

On the other hand, by means of DFT calculations, the authors elucidated the mechanism for Mg(NTf_2_)_2_**89**-catalyzed ring opening of terminal epoxides (Scheme 73). Here, magnesium **89** catalyzes the hydroboration of epoxides to afford the linear product (opposite to magnesium **46**). First, the authors ruled out the possibility of a magnesium hydride intermediate. However, they established that the epoxide coordinates to highly Lewis acidic **89**, which results in isomerization (**TS1** and **TS2**) to afford the corresponding aldehyde. Finally, after HBpin coordination to **89**, the aldehyde is reduced, affording the linear isomer product. Moreover, the authors also demonstrated a loss of enantioselectivities when enantiopure epoxide was tested with **89**, corroborating the epoxide isomerization *via* carbocation intermediate.

**Scheme 74:**
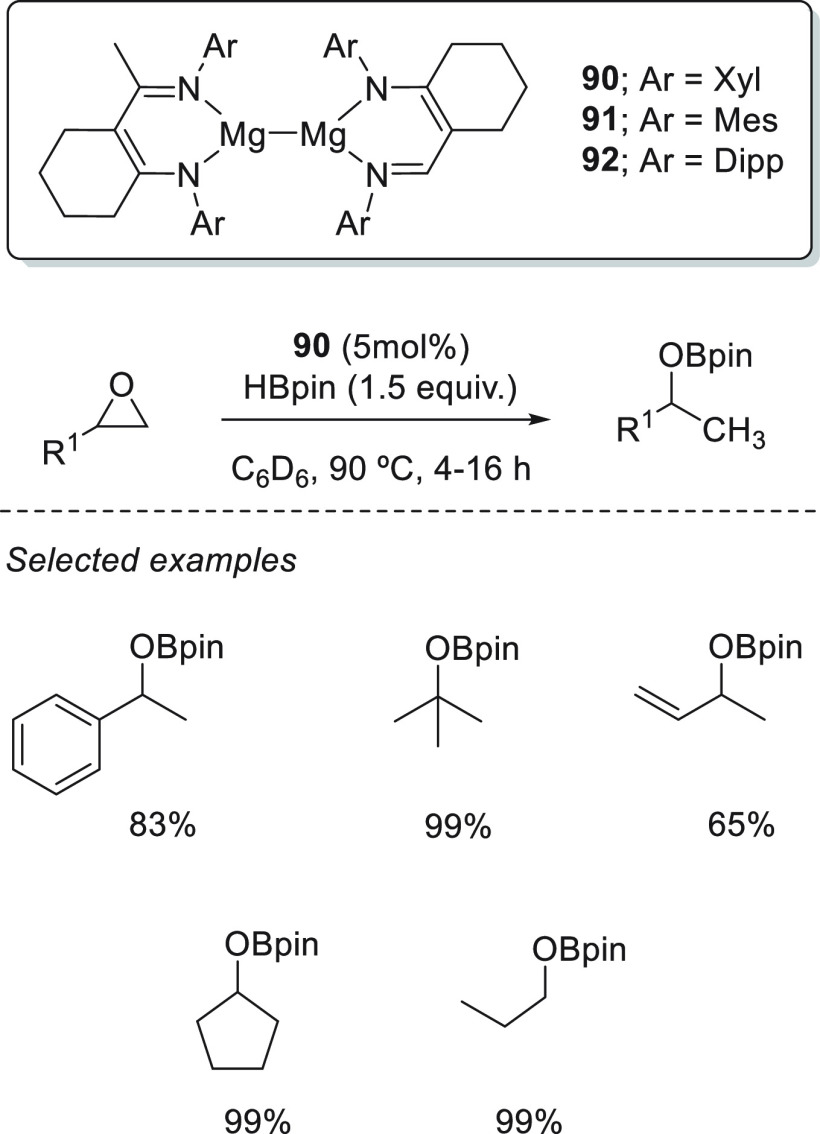
Magnesium(I) Dimer Catalyzed Hydroboration of Epoxides

Very recently, Ma *et al.* also reported the application of dimeric Mg(I) dimers **91**–**92** for the hydroboration of epoxides.^175^ In this case, Mg(I) dimers were found to be active as well (Scheme 74).

## 6 Comparison of Alkali- and Alkaline-Earth-Abundant Catalysts with Aluminum and Lanthanide and Early-Actinide Analogues

The application of main group metal catalysts in reactions that have been traditionally been associated with transition-metal complexes has increased exponentially in the past decade. Thus, p-block metals such as aluminum (and to a lesser extent, Sn and Ge) and f-block metal complexes such as lanthanides have been successfully applied for the hydroboration of a wide range of unsaturated systems.

Alkali- and alkaline-earth-abundant metals (s-block) share several similarities with p- and f-block metals, including:

(i) generally redox neutral catalytic activity;
(ii) metal hydride as active catalytic species;
(iii) mechanisms based on similar catalytic steps.

As such, it is interesting to compare alkali- and alkaline-earth-abundant metal catalysts (presented in this review) with their aluminum, lanthanide, and early actinide catalyst analogues. It is important to highlight that this section is not intended to provide a detailed description of the mechanisms and scope of the different p- and f-block metals. We will simply illustrate the best catalysts of each block and compare them with the best of s-block catalysts. The comparison with Sn- and Ge-based catalysts will not be discussed due to their limited scope as almost all examples are based on the hydroboration of aldehydes and ketones.^176,177^ However, it is worth mentioning that since the first example of low-valent Sn(II) and Ge(II) complexes applied in hydroboration of C=O bonds^178^ promising advances have been made on low-valent p-block metal hydroborations.^179−182^

### 6.1 s-Block Metals *versus* Aluminum Complexes

Group 13 hydrides have been widely used in various organic transformations, but their catalytic use has been rather scarce.^183^ Many efforts have been made to investigate the catalytic activity of aluminum hydrides as main group catalysts based on the principles of transition-metal catalysts.^184^

#### 6.1.1 Aldehydes and Ketones

**Scheme 75:**
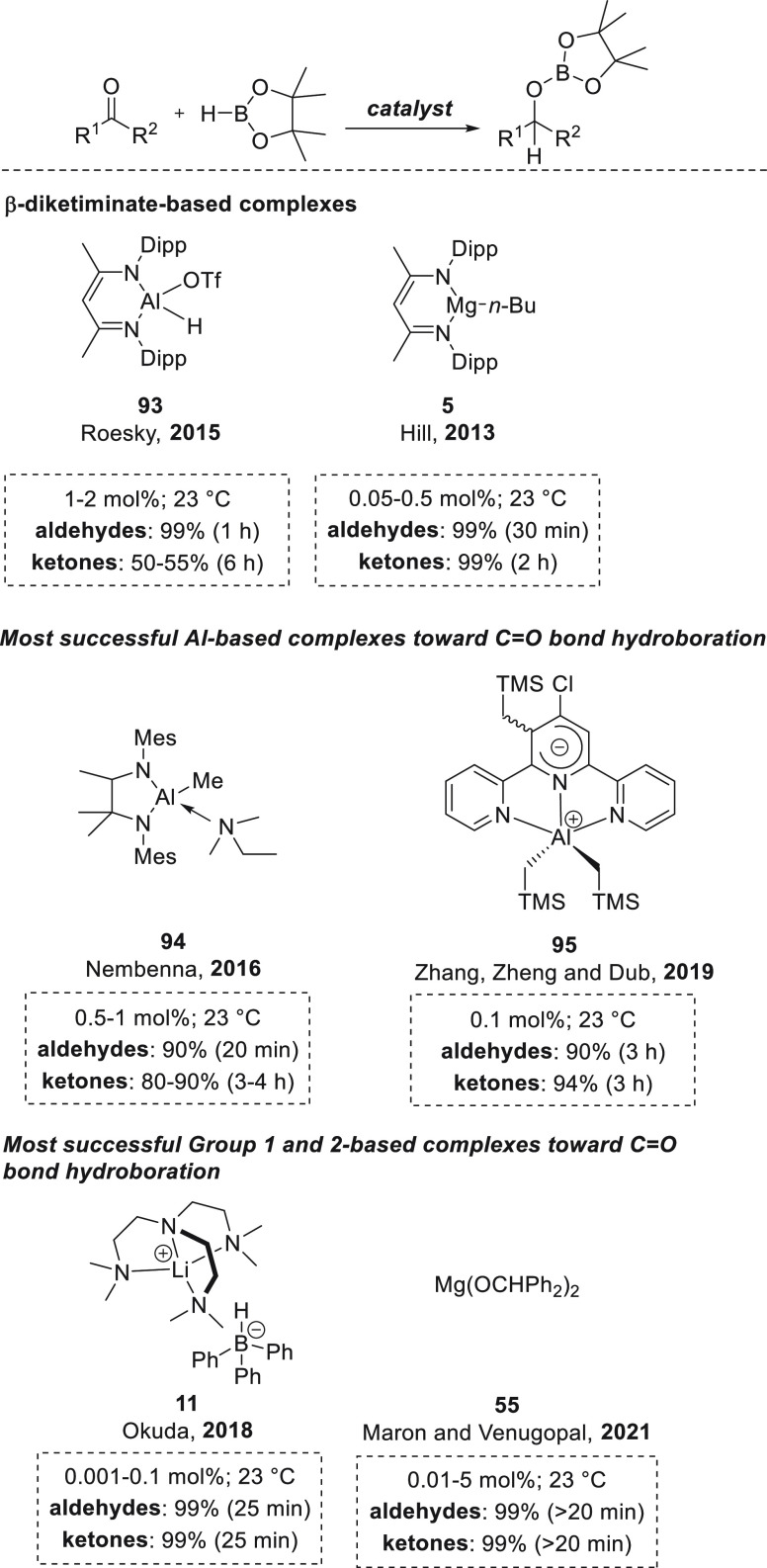
Comparison in the Hydroboration of Aldehydes and Ketones

The hydroboration of aldehydes and ketones has become a benchmark reaction to test the activity of catalysts. In this regard, several aluminum-based catalysts active for the reduction of C=O bonds have been developed (Scheme 75).^185−191^ Compared with Group 1 and Group 2 catalysts, however, application of aluminum catalysts (group 13) is still in its infancy. A comparison of β-diketiminate magnesium **5** with its aluminum analogue **93**(185) demonstrates that the magnesium complex **5** showed higher activities toward aldehyde and ketone hydroboration. This behavior can be attributed to the electronically difference of the metal centers but also to the fact that, whereas complex **5** contains an alkyl group as reactive side, aluminum **93** contains a hydride and a OTf group, which influences the reactivity. Although recent efforts have been made in aluminum catalysis, lithium complex **11** and magnesium complex **55**, developed by Okuda *et al.*(35) and Venugopal *et al.*,^98^ respectively, exhibit much higher reactivity. Mechanistically, Okuda (for **11**) and Maron and Venugopal (for **55**) discarded the fact that metal hydrides are the active species in the reduction of C=O bonds. In both cases, the authors suggest that the hydroboration of C=O bonds proceeds *via* B–H addition, either from the HBPh_3_ anion (in the case of **11**, Scheme 12) or from a ligand activation of HBpin (in the case of **55**, Scheme 26). In the other cases, a metal hydride species is proposed as active catalysts; therefore, a different mechanism operates when complexes **94** and **95** are used

##### Enantioselective Hydroboration of Ketones

**Scheme 76:**
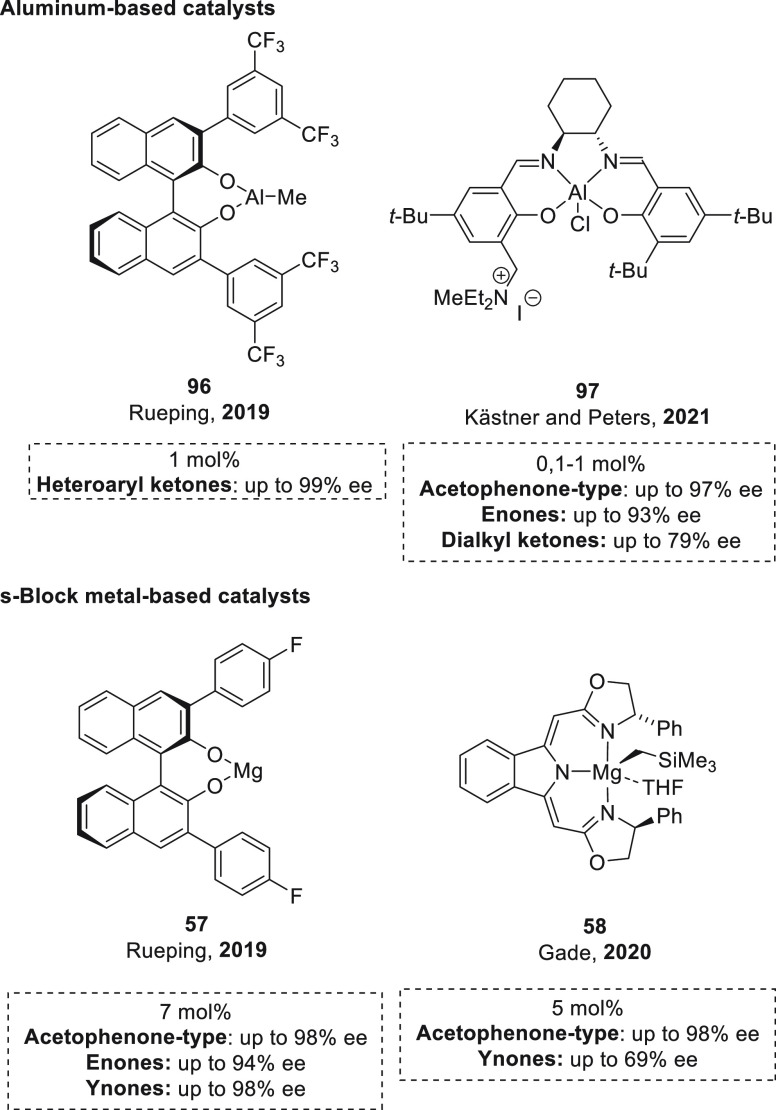
Comparison in the Enantioselective Hydroboration of Aldehydes and Ketones

The hydroboration of ketones remains one of the most studied transformations with the use of main group metals. However, the enantioselective version remains a challenge. Similar to magnesium-based complexes, there are only a few active and selective aluminum catalysts (Scheme 76).^192−195^ In this regard, results complementary to those obtained using magnesium **57**(107) and **58**(109) can be obtained by aluminum–BINOL complex **96** developed by Rueping *et al.*(194) and a recent aluminum–ammonium salt **97** developed by Kästner, Peters, and co-workers.^195^ The different reactivity observed between Al-BINOL **96** and Mg-BINOL **57** can be attributed to the different active species formed in the presence of pinacolborane. In the reaction with **96**, the authors report the formation of active aluminum hydride species, while for **57**, the authors suggest a metal–ligand cooperative activation of pinacolborane. In the case of aluminum **97**, the ammonium salt activates the borane while the aluminum center activates the carbonyl compound. As such, very different active species and mechanisms are reported, thus making the comparison difficult. Nevertheless, main group metal complexes mimicking the dual activation mode of **57** and **97** can be excellent candidates for active and selective catalysts toward enantioselective hydroboration of ketones.

#### 6.1.2 Nitriles and Carbodiimides

**Scheme 77:**
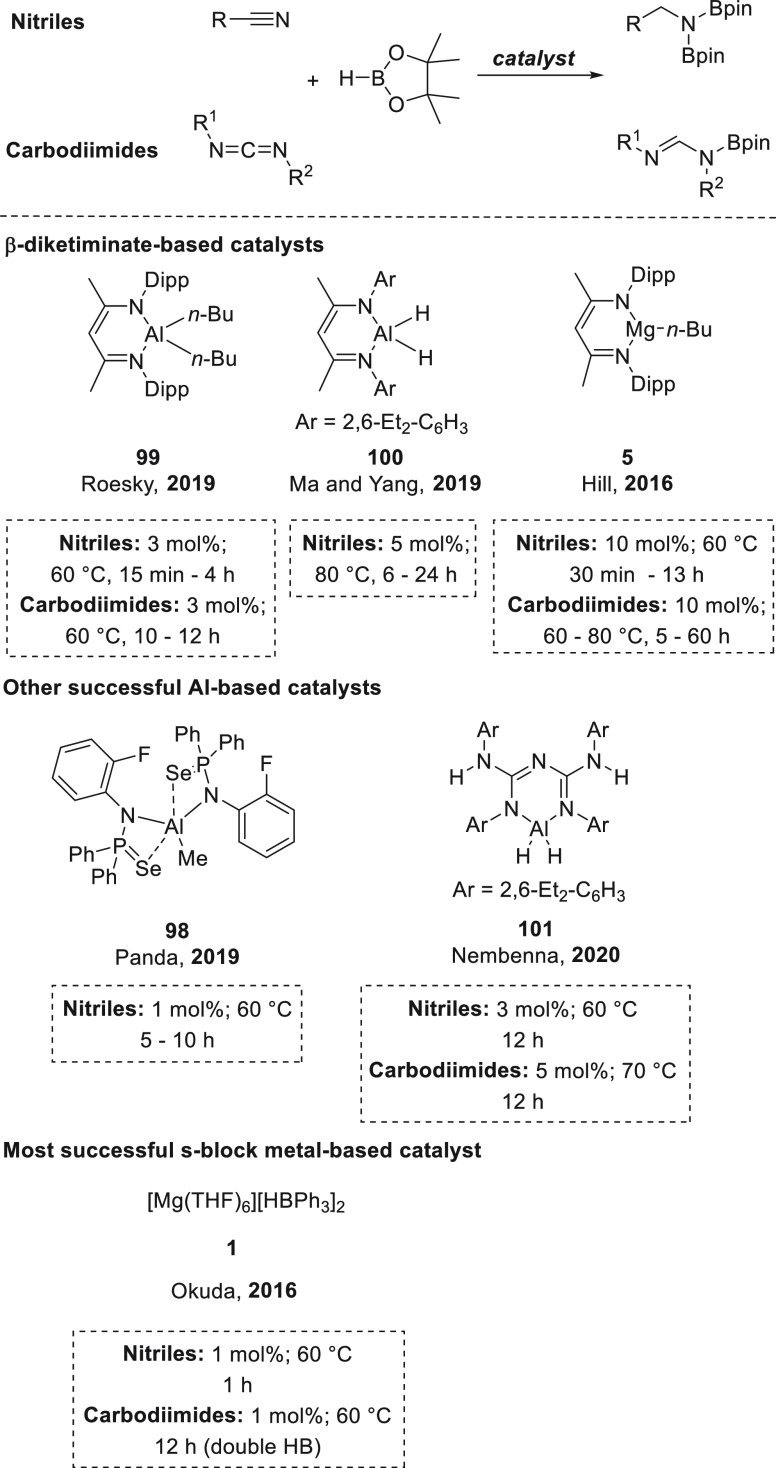
Comparison in the Hydroboration of Nitriles and Carbodiimides

Recently, nitriles and carbodiimides have become a benchmark reaction to test the newly developed aluminum catalysts.^196,197^ Whereas Hill *et al.* developed the first s-block metal complex **5** active toward nitrile^143^ and carbodiimide^144^ hydroboration in 2016, Panda *et al.* reported the first aluminum-based precatalyst **98** in 2019.^198^ A comparison of the β-diketiminato complexes (Scheme 77) shows that Al-based complex **99** developed by Roesky^199^ is more active than its magnesium analogue **5** in both the hydroboration of nitriles and carbodiimides.^143,144^ Thus, the different electronic natures of the Al- and Mg-atoms play a crucial role. At the same time, aluminum complex **98** developed by Panda is the most active aluminum complex reported to date for the hydroboration of nitriles.^198^ However, cationic magnesium complex **1** developed by Okuda is still the most active catalytic system for the hydroboration of nitriles and carbodiimides.^45^

#### 6.1.3 Carbon Dioxide

**Scheme 78:**
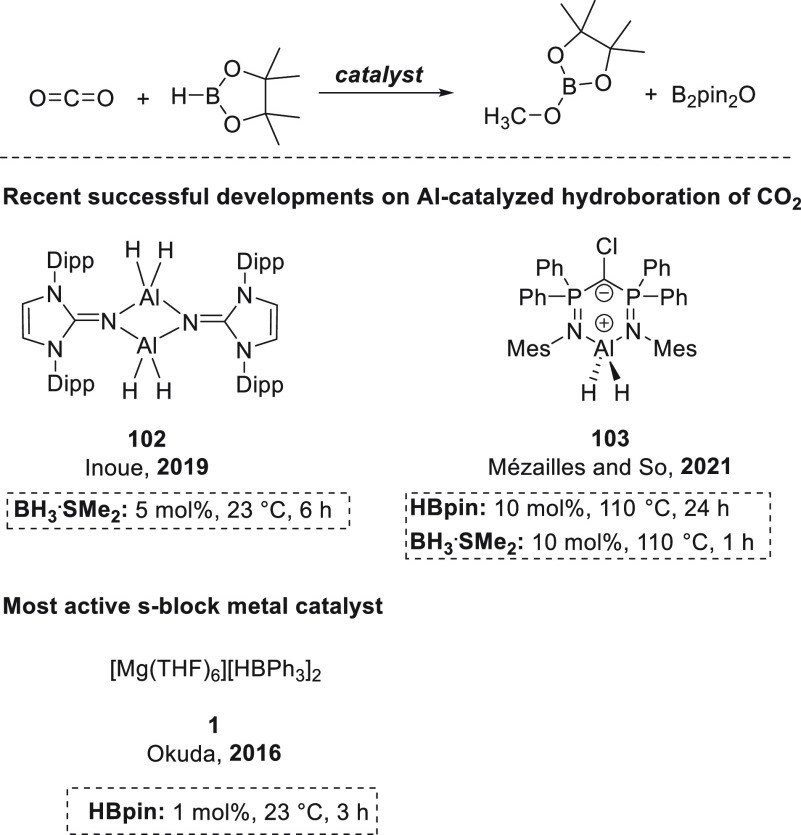
Comparison in the Hydroboration of Carbon Dioxide

The hydroboration of carbon dioxide has not been studied as much as the hydroboration of other C=O bonds. Although recent advances have been made in aluminum catalysis by Inoue *et al.*(200) and Mézailles, So, *et al.*(201) dicationic magnesium catalyst **1** developed by Okuda exhibits the highest activity (Scheme 78).^45^ One can attribute the higher catalytic activity of magnesium **1** to its different reactivity. Whereas the aluminum complexes **102** and **103** undergo 1,2-hydroalumination, in the case of cationic magnesium **1**, the hydroborated anion plays a crucial role *via* direct B–H addition (Scheme 12).

#### 6.1.4 Alkenes

**Scheme 79:**
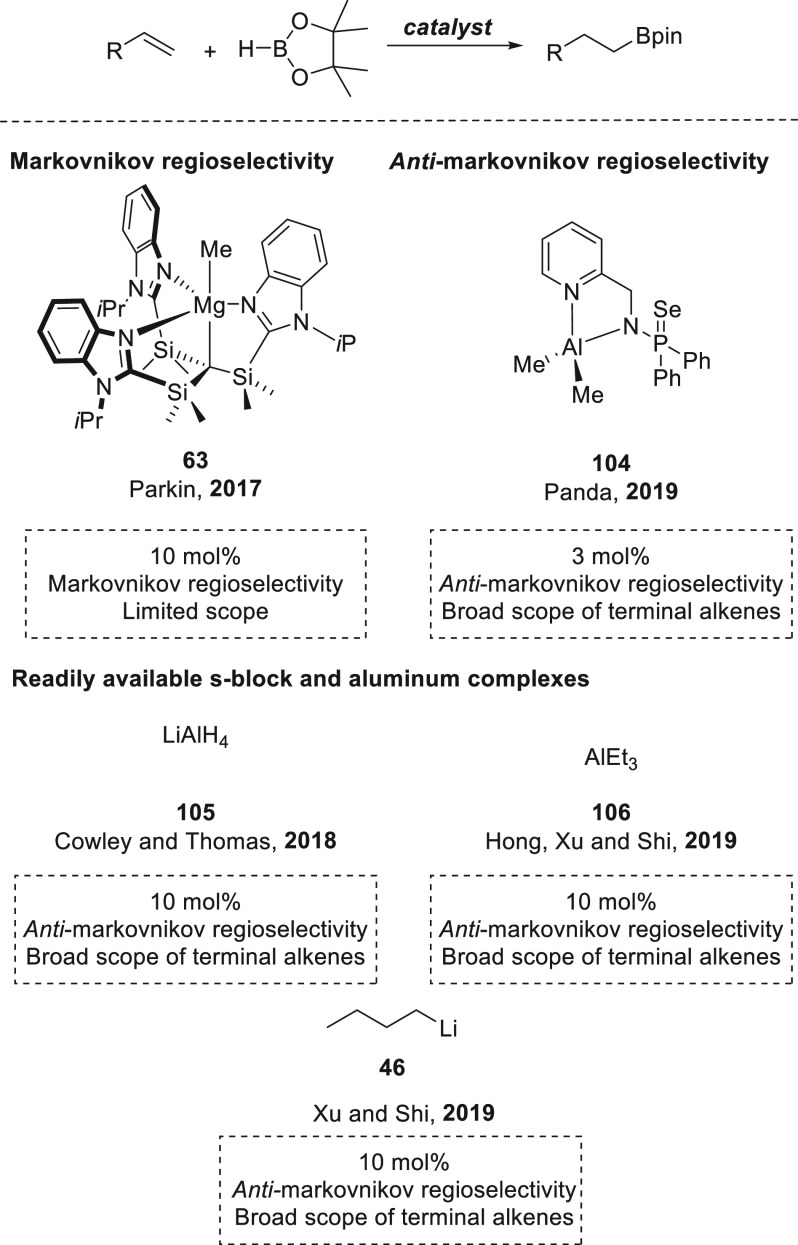
Comparison in the Hydroboration of Alkenes

Contrary to the hydroboration of highly polarized C=O and C=N bonds, there are more active aluminum complexes reported for hydroboration of C=C bonds than the s-block analogues.^202^ In this regard, Cowley and Thomas applied commercially available LiAlH_4_**105** for the hydroboration of a wide range of terminal alkenes to obtain the *anti*-Markovnikov regioisomer.^203^ Similarly and independently, Panda^204^ and Shi^205^ applied active aluminum precatalyst **104** and **106** (Scheme 79). A comparison of commercially available alkyl metals such as AlEt_3_**106** and *n*-BuLi **46**,^164^ shows that both provide similar activities and regioselectivities. Interestingly, magnesium **63**, developed by Parkin, is the only main group metal catalyst that affords the Markovnikov product to date, most likely due to the ligand backbone.^118^

#### 6.1.5 Alkynes

**Scheme 80:**
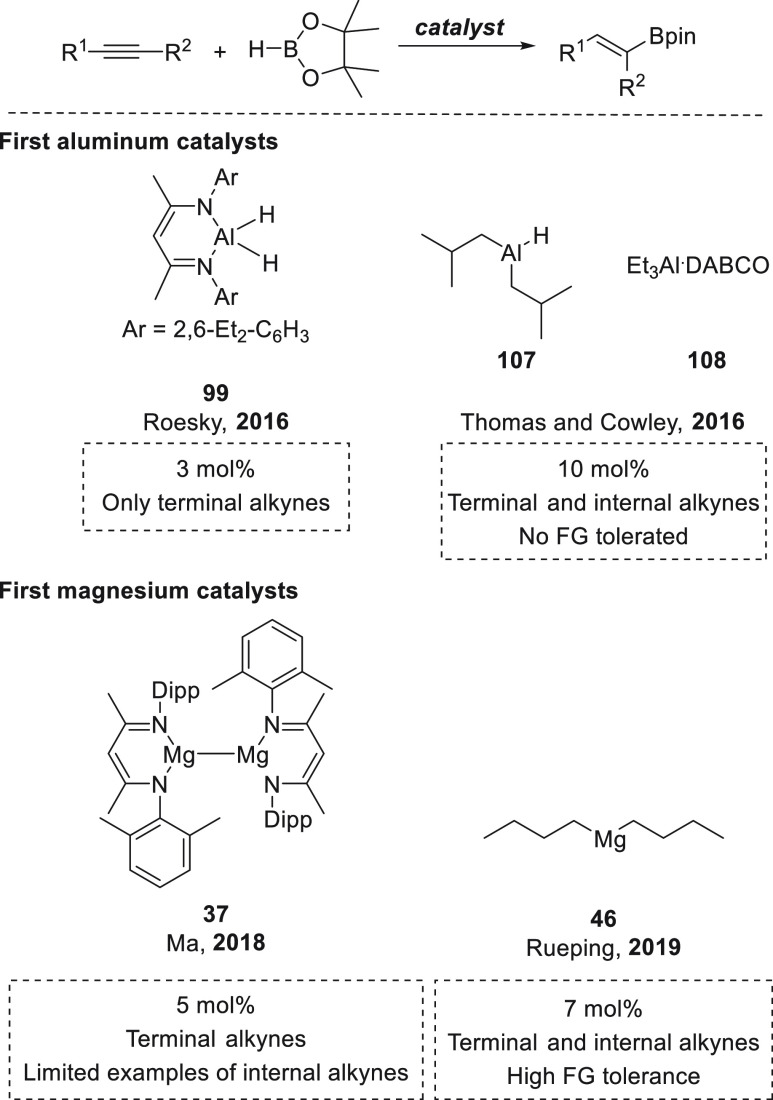
Comparison in the Hydroboration of Alkynes

Similarly than hydroboration of C=C bonds, the hydroboration of alkynes has been widely studied using aluminum catalysts (Scheme 80).^204−206^ In fact, the first application of aluminum catalysts was reported by Roesky in 2016, two years before the first magnesium complex.^168^

Interestingly, Thomas and Cowley applied commercially available alkyl aluminum **107** and **108** for the hydroboration of terminal and internal alkynes. However, the functional group tolerance was limited.^169^ Comparison of **99** with **107** and **108** shows that the latter precatalysts can catalyze the hydroboration of internal alkynes, probably due to the lower steric hindrance around the metal center. On the other hand, magnesium complexes developed by Ma (**37**) and Rueping (**46**) showed broader substrate scope with excellent functional group tolerance, probably due to the milder reaction conditions used, when compared to its aluminum analogue **108** (80 °C for **46***vs* 110 °C for **108**).

### 6.2 s-Block Metals *versus* Lanthanide and Early-Actinide Complexes

Because of their low cost and toxicity and high catalytic activity, f-block elements, which are relatively highly abundant in the Earth’s crust, have been widely used in catalytic hydrofunctionalization of unsaturated bonds.^207,208^

Similar to s-block metals and aluminum, organolanthanide and early actinide catalysts engage in redox-neutral processes. Thus, in hydroboration reactions the mechanism resembles the one presented for alkali- and alkaline-earth-abundant metals. Another similarity is the σ-bond metathesis pathway occurring in the presence of hydridic reagents such as pincacolborane. As such, organolanthanides and early actinides form catalytically active Ln–H and An–H species.^209,210^ The catalytic activities of f-block metal complexes are significantly influenced by the nature of the metal center and the steric and electronic nature of the ligand. Thus, for f-block metal-catalyzed hydroelementations, larger metal ions bearing a less sterically hindered coordination sphere show higher activity.^211,212^ However, due to the high oxophilicity of the Ln- and An-centers, thermodynamically stable and catalytically inactive Ln–O and An–O bonds are preferred, making the hydroboration of C=O bonds rather challenging.^213,214^

In this part, we will compare the most active and selective f-block metal-based complexes with s-block metal catalysts in the hydroboration of unsaturated systems.

#### 6.2.1 Aldehydes and Ketones

**Scheme 81:**
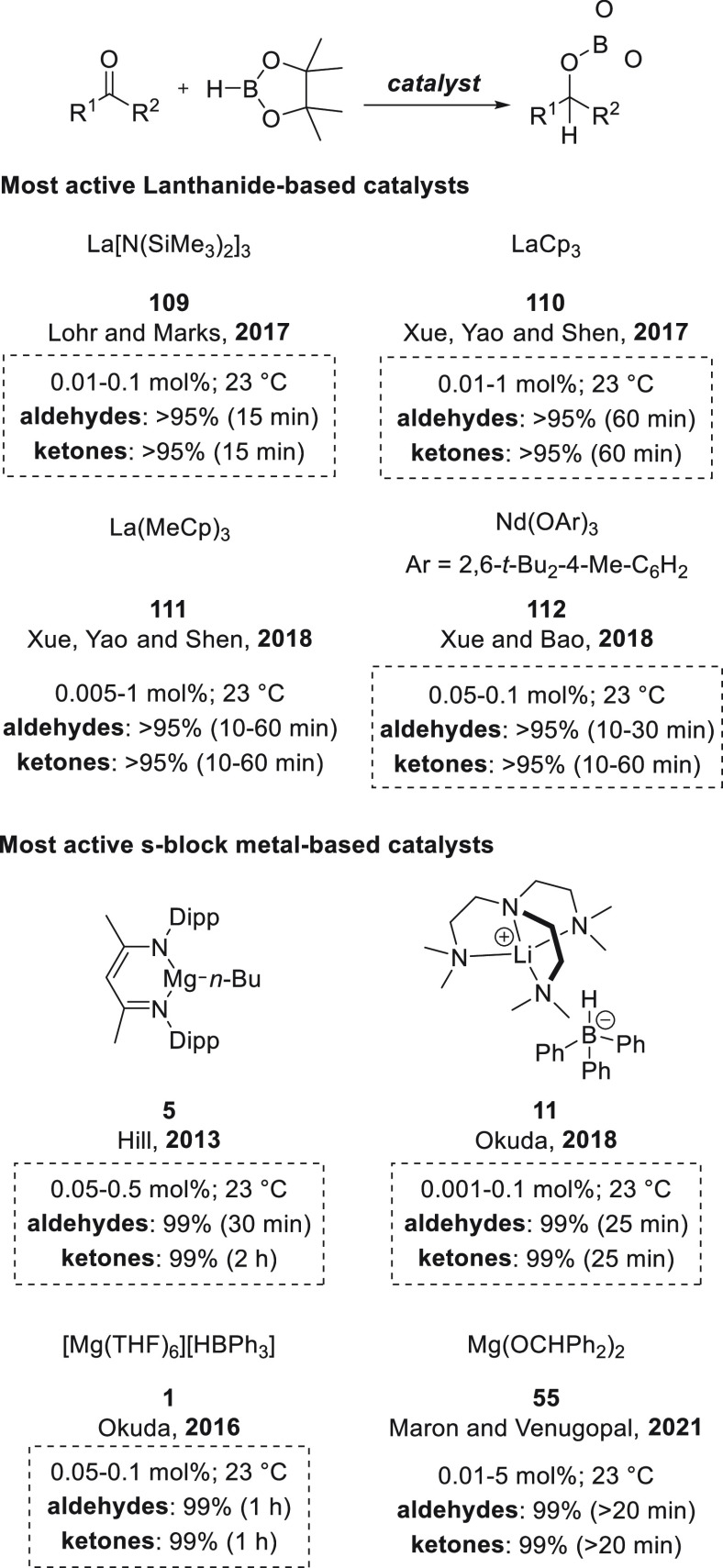
Comparison in the Hydroboration of Aldehydes and Ketones

The wider exploration of lanthanide-based catalyst for the hydroboration of aldehydes and ketones started later than s-block metal complexes.^215^ Simple La–amide,^215^ −cyclopentadienyl,^216,217^ and −alkoxide^218^ complexes were found to be very active, competing favorably with the best group 1 and group 2 metal complexes (Scheme 81). However, Okuda’s Li complex **11** remains the most active catalyst reported to date.^35^ Generally, metal complexes that form metal hydrides upon reaction with boranes show lower activity than those that, for instance, activate pinacolborane *via* nucleophilic attack or Lewis acid-type coordination (**1**, **11**, and **55**).

Regarding Ln reactivity, La-amide **109**, which bears a *N*-ligand, has been shown to be the most active Ln catalyst.

##### Enantioselective Hydroboration of Ketones

**Scheme 82:**
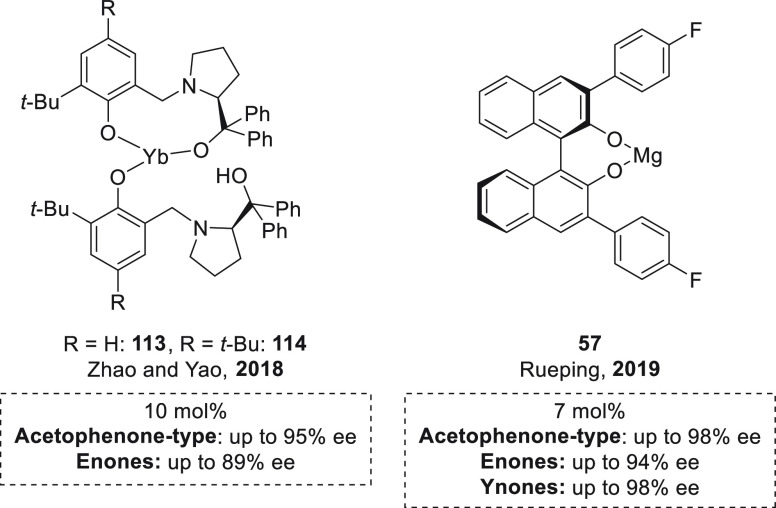
Comparison in the Enantioselective Hydroboration of Ketones

As described above the successful applications of chiral alkali- and alkaline-earth-abundant metals for the enantioselective hydroboration of ketones are rare.^107,109^ However, the examples of rare-earth metal complexes are even more unusual (Scheme 82). In this regard, the only example is the phenoxy-prolinol Yb catalysts **113** and **114** reported by Zhao and Yao.^219^ Compared with magnesium complex **57** reported by Rueping *et al.*, enantioselectivities reported in the hydroboration of acetophenone derivatives and enones are lower.^107^

#### 6.2.2 Pyridines

**Scheme 83:**
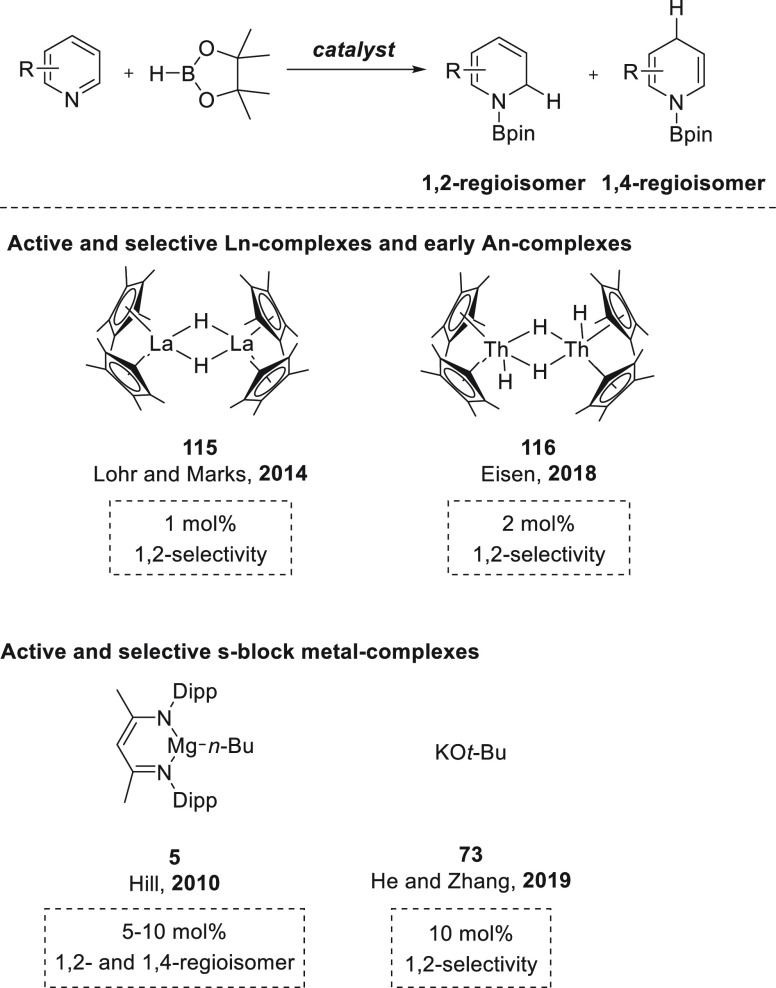
Comparison in the Hydroboration of Pyridine and Derivatives

The first s-block metal-catalyzed hydroboration of *N*-heterocycles was reported by Hill *et al.* in 2011 when magnesium **5** catalyzed the hydroboration of pyridine.^115^ In 2014, Delferro and Marks *et al.* reported the first example of a La-based catalyst.^19^ Interestingly, dimeric [Cp*LaH]_2_ complex **115** exhibited complete selectivity toward 1,2-hydroborated pyridine, similar to the Th complex **116** developed later by Eisen (Scheme 83).^220^ Probably, due to the milder reaction conditions when compared to Mg precatalyst **5**, the kinetically favored 1,2-product remains in favor over the thermodynamically controlled 1,4-hydroborated product. It is important to highlight that comparable 1,2-regioselectivity was observed by He and Zhang when KO-*t*-Bu **73** was used as a precatalyst (Scheme 35).^123^

#### 6.2.3 Imines and Nitriles

**Scheme 84:**
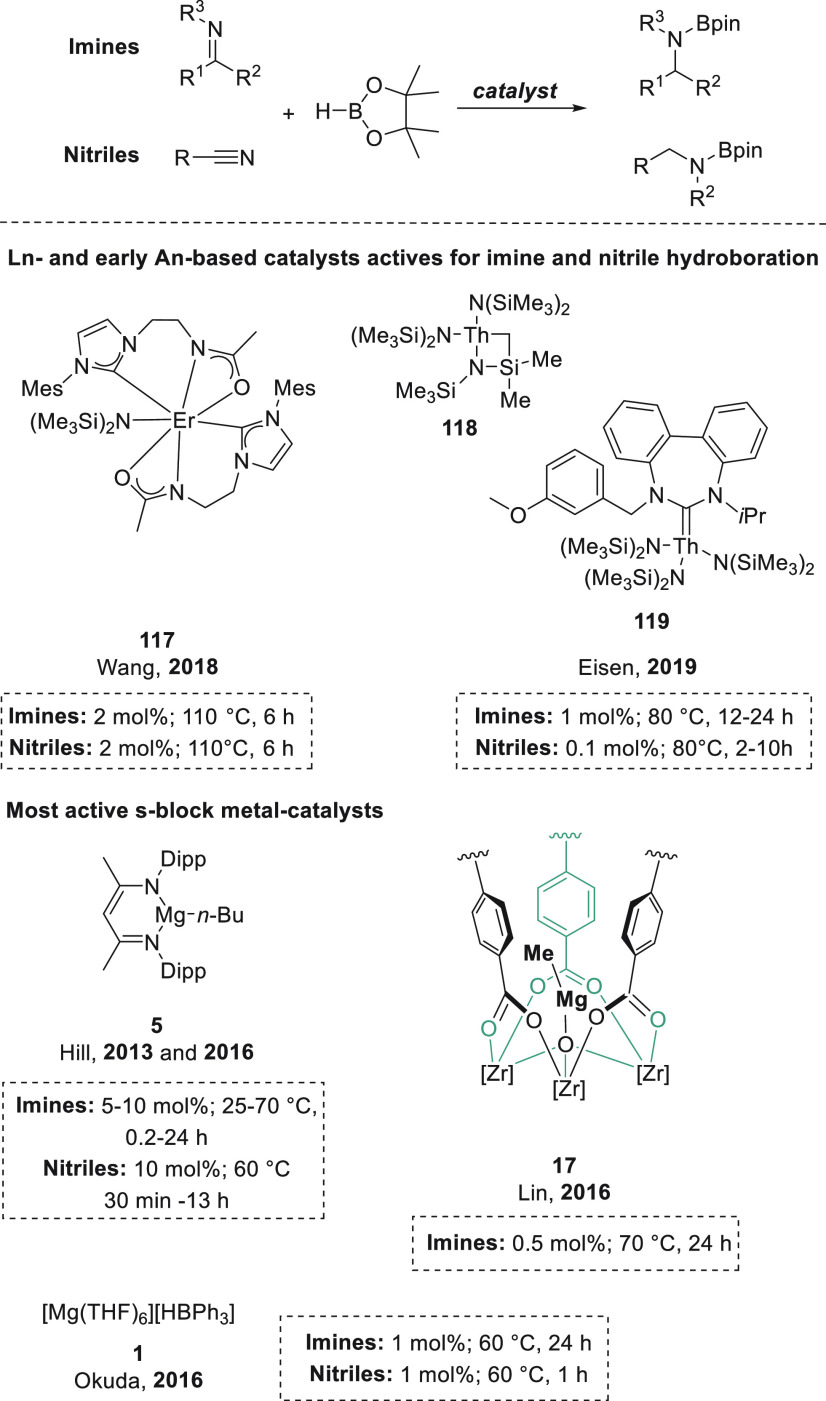
Comparison in the Hydroboration of Imines and Nitriles

The first s-block metal complex active toward imine^125^ and nitrile^144^ reduction was developed by Hill *et al.* in 2013 and 2016, respectively. Two years later, the first successful example of a rare-earth metal catalyst was reported by Wang.^221^ However, with magnesium precatalyst **1** or a rare-earth metal complex **117**, long reaction times and high temperatures are needed. Later, Eisen *et al.* reported the Th-catalyzed hydroboration of imines and nitriles.^222^ Comparing Ln- (**117**) and An-based complexes (**118** and **119**) with s-block metal catalysts (**1**, **5**, and **17**), one can see that, whereas for the hydroboration of imines, long reaction times are needed in all cases, for the nitrile hydroboration magnesium complex **1** is the most active metal complex (Scheme 84).^45^

#### 6.2.4 Esters and Amides

**Scheme 85:**
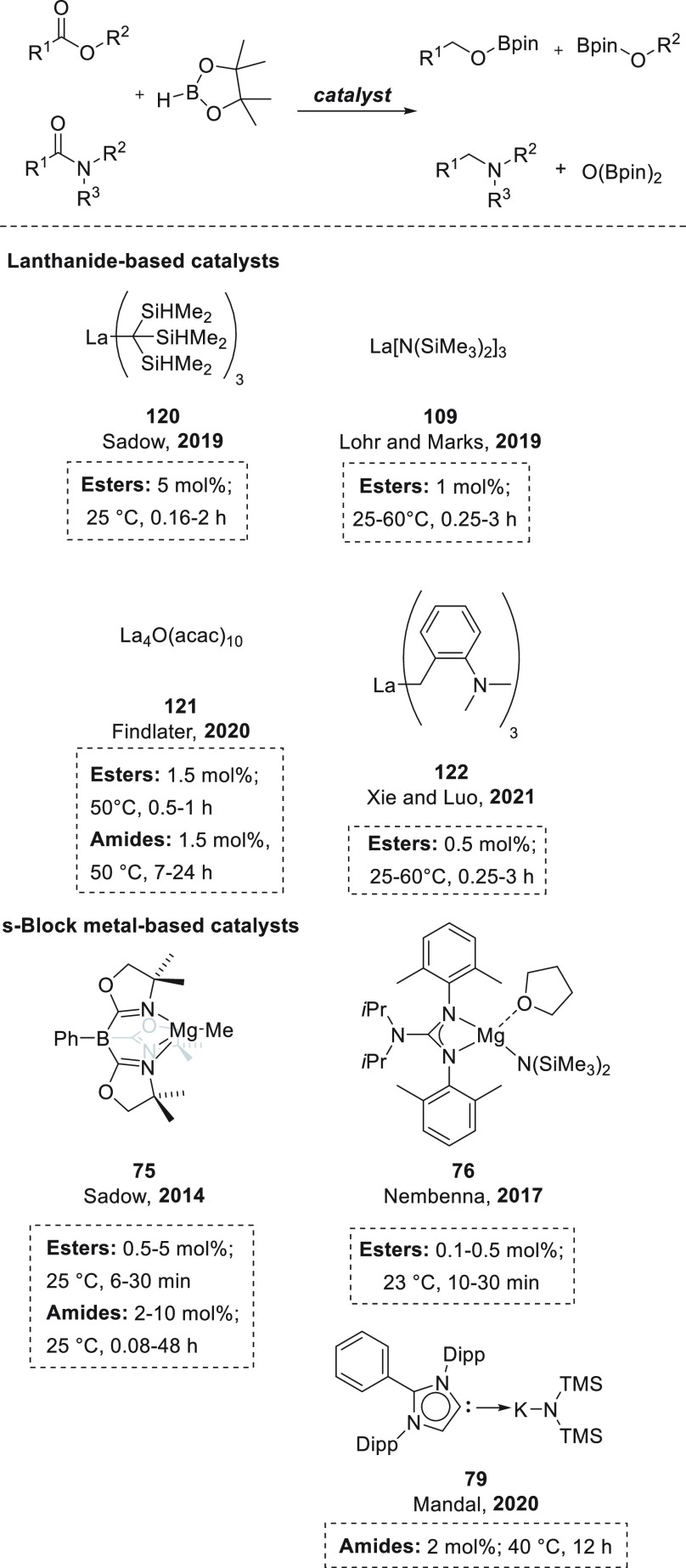
Comparison in the Hydroboration of Esters and Amides

While the first s-block magnesium-catalyzed hydroboration of esters^132^ and amides^133^ was reported by Sadow in 2014 and 2015 (**75**), the first example using a lanthanum-based precatalyst (**120**) was reported in 2019 by the same author.^20^ Since then, other La-based complexes have been reported as active precatalyst toward esters and amides.^223−226^ When comparing La with Mg complexes, we can observe that both types of complexes exhibit similar reactivity (Scheme 85).

#### 6.2.5 Alkenes

**Scheme 86:**
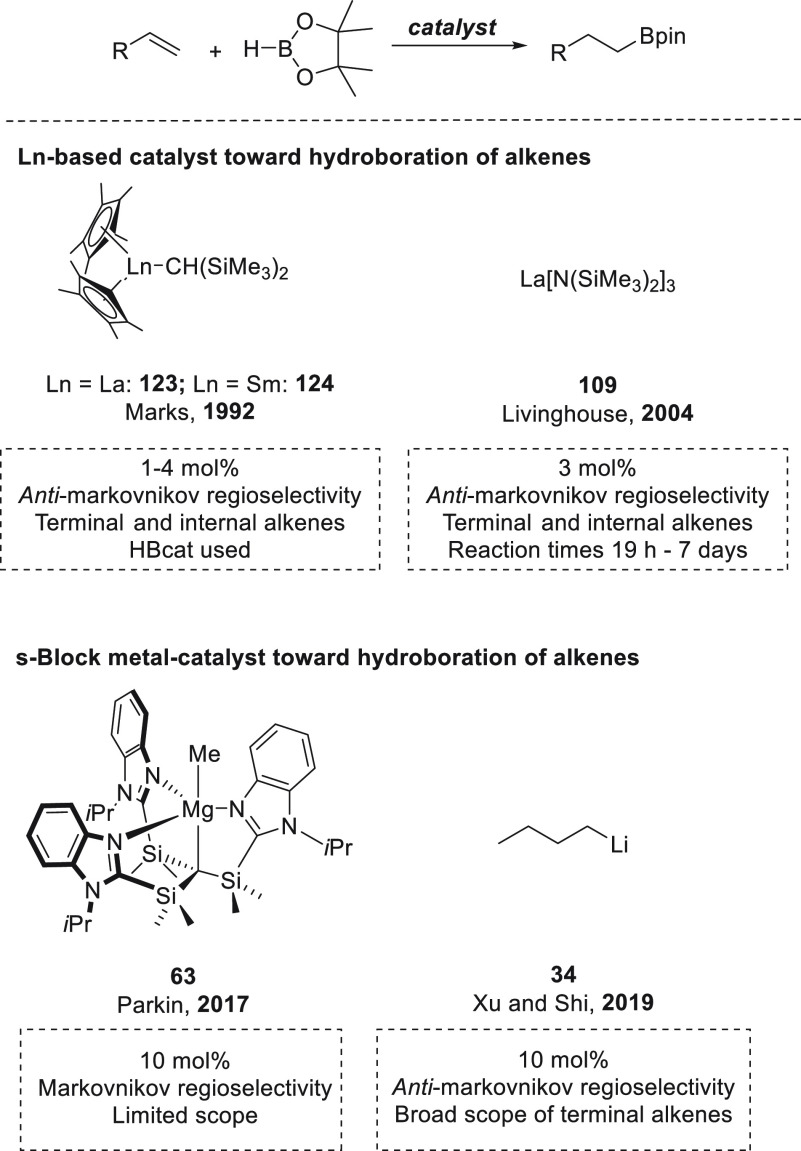
Comparison in the Hydroboration of Alkenes

In 1992, Marks *et al.* reported the first example of organolanthanide-catalyzed (**123**) hydroboration of olefins.^18^ In this case, HBcat was used to hydroborate terminal and internal alkenes. With the recent application of s-block metal catalytic systems, broader substrate scope is tolerated under milder reaction conditions and shorter reaction times. Similar to aluminum-based complexes and most of the s-block metal complexes, Ln-based catalysts ensure the *anti*-Markovnikov regioselectivity (Scheme 86).^227^

#### 6.2.6 Epoxides

**Scheme 87:**
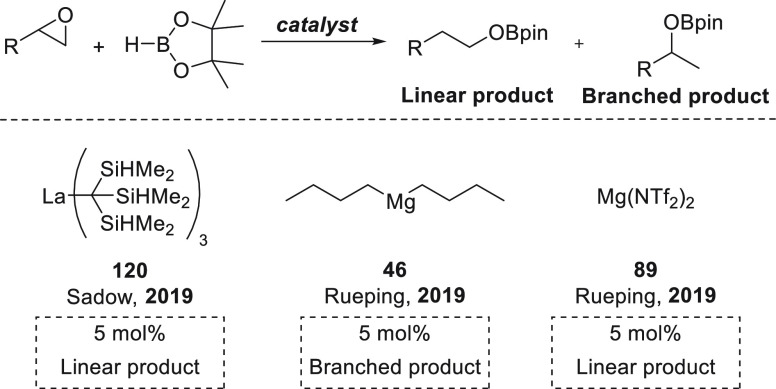
Comparison in the Hydroboration of Epoxides

In 2019, Sadow *et al.* reported the use of tris(alkyl)lanthanum **120** for the hydroboration of epoxides.^20^ One year later, Rueping *et al.* reported the use of commercially available dialkylmagnesium Mg(*n*-Bu)_2_**46** as a precatalyst.^173^ Interestingly, while the lanthanide-based precatalyst provided the linear isomer, the magnesium **46** provided the branched isomer. This complementary result can be explained by the different catalytically active species formed. Whereas La complex **120** activates pinacolborane forming a zwitterionic species,^20^ Mg complex **46** forms an active hydride species.^173^ On the other hand, Mg complex **89**, which does not react with HBpin forming a magnesium hydride species, provides the same regioisomer as **120** (Scheme 87).^173^

## 7 Considerations

### 7.1 Borane Decomposition: BH_3_ as a “Hidden” Catalyst

Recently, Thomas *et al.* demonstrated that catalyst precursors can mediate BH_3_ formation by the decomposition of HBpin in the presence of a nucleophilic catalyst.^31,32^ The authors point out that in the case of reactions utilizing HBcat, decomposition is routinely investigated. In the case of HBpin, however, decomposition is not commonly considered as it is perceived as a stable hydride source. The authors applied different well-established precatalysts (including Group 1 and Group 2 metal-based complexes) for hydroboration of *e.g.* alkenes, alkynes,^228^ ketones and *N*-heterocycles and in many cases observed formation of BH_3_ in the reaction mixture. This *in situ* formed species then acted as a “hidden” catalyst.

Since the hydroboration of a variety of organic compounds can be mediated by simple nucleophiles, careful control is needed to determine whether the catalyst studied is the “true” catalyst or if BH_3_ formed, is the “hidden” active catalyst.

Recently reported enantioselective and regiodivergent hydroboration of unsaturated systems in the presence of well-defined alkali- and alkaline-earth-metal complexes, however, show that BH_3_ does not always have to be the “hidden” catalyst. If BH_3_ indeed acted as a hidden catalyst background reaction then racemic product formation would have been expected. Nevertheless, careful control experiments must be conducted when HBpin activation by group 1 and group 2 metals is studied.

Thomas also points out that commercially available HBpin may contain BH_3_ impurities which may compete with a catalyst. In this context, it is worth mentioning that some commonly occurring impurities may promote hydroboration as well. For instance, Speed reported that water or methanol impurities promoted hydroboration of imines.^229^

Very recently, Jones and co-workers reported that Mg(I) dimers also react with HBpin to provide derivatives in which the γ-carbon of the β-diketiminate ligand is activated by boron hydride. Additionally, different reactive boron-containing species such as boryloxides (OBpin), borates ([B(pin)_2_]^−^ or [(pin)BH_2_]^−^), B–O bond ruptured [pinBH_2_]^−^, or BH_3_ have been observed. These results suggest that magnesium(I) dimers are not catalysts in the hydroboration of unsaturated bonds and that there are many potential precatalysts or hydride sources that are generated when Mg(I) dimers and HBpin are mixed.^230^

Additionally, Jin and co-workers discovered that carboxylic acids may promote hydroboration of alkynes. However, in this case, elevated temperatures were required, and thus, acid impurities must also be taken into consideration.^231^ As such, as for any catalytic reaction, it is of great importance to consider all side products as potential catalysts.

### 7.2 Catalyst-Free Hydroboration

**Scheme 88:**
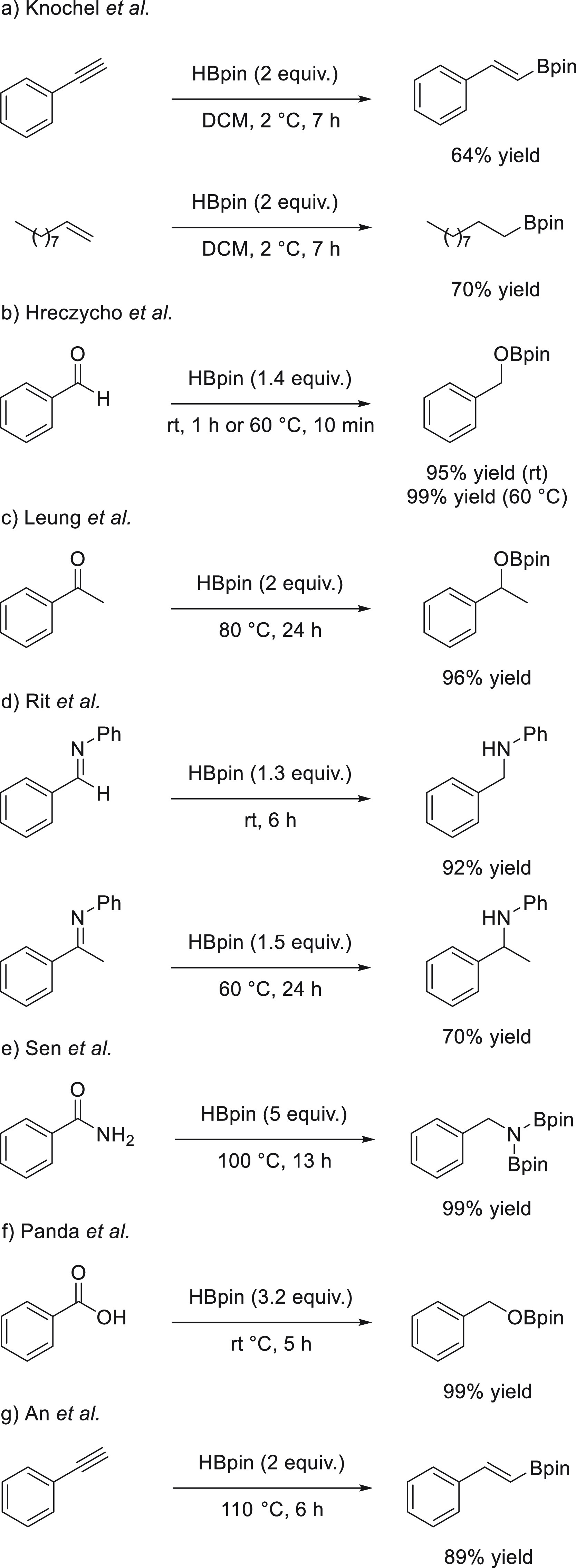
Catalyst-Free Reduction of Various Organic Compounds

In 1992, Knochel introduced a catalyst-free approach for selective hydroboration of alkynes and alkenes (Scheme 88a).^232^ In the presence of superstoichiometric quantities of HBpin (2 equiv), hydroboration of alkenes and alkynes proceeded in high yields under ambient reaction conditions. Since his discovery, catalyst-free approaches have been utilized for many functional group transformations. In 2018, Hreczycho *et al.* performed a solvent-free and catalyst-free hydroboration of aldehydes. The reaction proceeded rapidly at ambient temperature (Scheme 88b).^233^ The authors suggested that the reaction occurs through the formation of Lewis adducts with a weakened boron–hydrogen bond that facilitates the hydride transfer and reduction of the carbonyl bond. Leung *et al.* performed the reduction of ketones to achieve high conversion to the corresponding secondary alcohols at elevated temperature and long reaction times (Scheme 88c)^234^ and Rit *et al.* applied catalyst-free conditions for the hydroboration of aldimines and ketimines (Scheme 88d). The authors observed trends similar to those observed for the reduction of aldehydes and ketones. Whereas aldimines were hydroborated at room temperature, ketimines required elevated temperature and long reaction times.^235^ Very recently, Vanka, Sen *et al.* reported deoxygenative hydroboration of primary and secondary amides (Scheme 88e).^236^ The corresponding *N*-Bpin-protected amines were obtained with good to excellent yields; however, harsh reaction conditions, and in the case of secondary amides prolonged reaction times, were necessary. DFT calculations showed an energy barrier of 47.9 kcal mol^–1^ (Δ*G*^⧧^ value) which explains the need for elevated temperatures (100 °C). The groups of Panda,^237^ Ma,^238^ and Xue^239^ almost simultaneously reported hydroboration of carboxylic acids in the absence of any catalyst (Scheme 88f). All three groups suggested their own mechanisms; however, all start with a reaction between the acid and HBpin to form a boronic ester with concomitant release of hydrogen. Ma *et al.* suggest that this first step has an energy barrier of 56.8 kcal/mol. However, because the reaction is highly exothermic, this barrier is surpassed. Interestingly, the above-mentioned results contradict the paper on magnesium-based hydroboration of carboxylic acid by Ma *et al.* (see section 3.9). In the optimization table, the authors show that in the case of the absence of the catalyst very poor conversion for the reduction of model benzoic acid is observed (3.1 equiv of HBpin, 60 °C, 1 h, 40% yield).^154^ The same group, however, reports full conversion of the same model substrate under solvent-free and catalyst-free conditions (4 equiv of HBpin, 60 °C, 1 h, 99% yield).^238^ Similarly, Xue *et al.* reported full conversion already at room temperature, although after a slightly longer time (3.3 equiv of HBpin, rt, 4 h, 95% yield).^239^ As such, further studies are required to understand the reaction pathway. Finally, An *et al.* reported a catalyst-free hydroboration of alkynes (Scheme 88g). The authors postulate that hydroboration of alkynes proceeded in a general *syn*-addition to afford the trans hydroboration product as a result of thermal activation (110 °C).^240^

Overall, most of the above-mentioned hydroborations under catalyst-free and solvent-free conditions using HBpin as a reducing agent require elevated temperatures, long reaction times, or superstoichiometric amounts of HBpin to achieve full conversions. On the other hand, catalytic systems based on s-block metals usually present milder reaction conditions and shorter reaction times. For example, when two papers reported by Hreczycho on hydroboration of aldehydes are compared, the one that utilizes a catalyst (LiHBEt_3_)^90^ shows much better results than its catalyst-free analogue.^233^ Another example is the Mg-based catalytic system reported by Rueping which requires 80 °C for the hydroboration of alkynes,^171^ while the catalyst-free protocol requires 110 °C.^240^ However, comparison of the activity of the catalyst-free approach for the reduction of amides^236^ shows similar efficiency to s-block metals.^133,136,137,139^ In this context, it is worth mentioning, that reduction of tertiary amides was not possible when a catalyst-free system was utilized, whereas in the case of some of the s-block metal catalysts, this reaction was possible.^133,136,137^

**Scheme 89:**
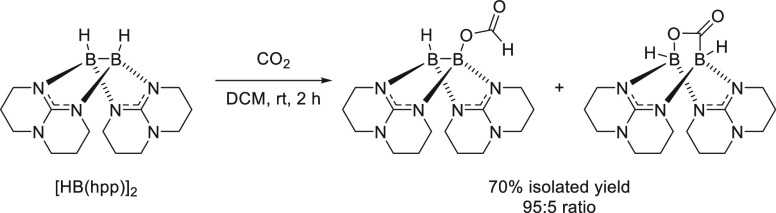
Catalyst-Free Reduction of Carbon Dioxide

Therefore, to further improve the efficiency of catalyst-free systems, more reactive boranes may be introduced. In this context, Himmel *et al.* reported the use of nucleophilic diborane [HB(hpp)]_2_ for the hydroboration of carbon dioxide.^241^ The first hydroboration takes place at remarkably low temperature and short reaction time (Scheme 89). Further reduction of the so-obtained products was possible, when 9-BBN was added. The results obtained for CO_2_ reduction utilizing HBpin and the most active alkaline-earth-metal-based catalysts (see Section 3.8) compete favorably with the catalyst-free variant.

## 8 Conclusions and Outlook

In the past decade, alkali and alkaline earth metals have emerged as redox-neutral alternatives to transition-metal catalysts for the hydrofunctionalization of unsaturated bonds. In this Review, we describe the Group 1 and Group 2 metal catalysts applied for the hydroboration of various polarized unsaturated C=X as well as C–C multiple bonds. We discussed the synthesis of different s-block metal complexes, the scope of the hydroborations, and the proposed outcome. Finally, the comparison of these s-block metal complexes with other redox-neutral catalytic systems based on p-block metals such as aluminum and f-block metal complexes such as lanthanides and early actinides has been also presented.

Since the first example of an s-block metal-catalyzed hydroboration reaction, the evolution of this topic of research has been exponential. Regarding achiral hydroborations, alkali- and alkaline-earth metals bearing neutral and monoanionic ligands have been successfully reported as active and selective catalysts. The ligand design principles can be summarized as (i) the use of monoanionic or dianionic ligands to favor a strong metal–ligand binding, thus avoiding ligand redistribution, and (ii) bulky substituents in a close proximity to the metal center to avoid side-reactivity such as polymerization, catalyst decomposition, and/or ligand redistribution.

Moreover, in recent years, the use of commercially and readily available s-block metal precatalysts has become a focus of interest due to their low cost, simplicity, and thus, the avoidance of tedious ligand synthesis. The recent reports of the application of simple s-block organometallics in the hydroboration of a wide range of unsaturated systems have shown that these simple reagents are very active and selective and can be seen as good alternatives to those s-block metal complexes bearing elaborated ligands.

However, one has to take into consideration that some of the readily available organometallic can decompose pinacolborane (or other organic boranes) to form BH_3_, which has been shown to be an active hydroborating agent. Thus, careful control experiments must be conducted when the HBpin activation by s-block metals is studied.

Concerning the metals of interest, lithium and magnesium complexes have been the most studied catalytic precursors for the hydroboration of unsaturated polarized and unpolarized bonds, which has been the most studied hydrofunctionalization reaction. The combination of experimental and theoretical studies has provided insight into different mechanisms for alkali and alkaline earth metal complexes, all involving redox-neutral pathways. Whereas some mechanisms are based on σ-bond metathesis for precatalyst activation, leading to the formation of active metal hydride species, other mechanisms discard the formation of metal hydrides and rely on the formation of zwitterionic species from the reaction of catalyst precursor and HBpin. Hence, different mechanisms need to be considered: (i) in some cases, s-block metals act as active precatalysts due to the formation of active metal hydrides (*via* σ-bond metathesis with pinacolborane) and undergo 1,2-hydrometalation with the unsaturated bond; (ii) in other cases, the s-block metals activate the borane (*via* nucleophilic attack) and the newly formed borate is the active species, transferring the hydride to the unsaturated substrate. Here the question arises if the s-block metal is just a counterion of the nucleophile or if it also has a role in the activation of the unsaturated system *via* coordination. Given the early stage of s-block metal-catalyzed hydroborations of unsaturated systems, more efforts have to be made to fully understand the respective mechanisms.

Moreover, whereas the addition of H–B bonds to reactive C=O and C=N bonds has been widely studied, more recently, the hydroboration of less reactive bonds such as carbon dioxides and derivatives (carbonates and carbamates), alkenes, and alkynes, and strained systems such as epoxides and oxetanes has also been accomplished. Thus, the hydroboration reaction has become a useful tool for the synthesis of fine chemicals and the conversion of greenhouse gas to C1 building blocks.

Although the use of s-block metal catalysts for achiral hydroboration has evolved exponentially and can already been seen as a real alternative to transition-metal catalysts, the application of alkali- and alkaline-earth-abundant metal catalysts to asymmetric hydroboration is still underexplored. In this regard, there are only a few chiral catalysts reported which so far only focus on the hydroboration of ketones and which show lower catalytic activity and functional group tolerance if compared to the best transition-metal-based catalysts. Thus, the development of efficient chiral s-block metal catalysts is greatly desirable. For this purpose, an efficient ligand design is of high interest to avoid any kind of ligand redistribution, known as Schlenk-type equilibrium, which leads to very reactive but nonchiral species and, consequently, no enantioselective induction.

Comparing s-block metal catalysts with their p- and f-block analogues, we can observe several similarities:

(i) Simple organometallic compounds have appeared as precatalyst that can be an attractive alternative to catalysts with complex ligand architectures. They show excellent activities toward the hydroboration of several unsaturated bonds and high functional group tolerance.
(ii) Whereas for some unsaturated bonds s-, p-, and f-block metal catalysts show similar activities and selectivities, for other C=X bonds (such as aldehydes and ketones) lithium and magnesium complexes show higher activities than their aluminum and lanthanide analogues.
(iii) Regarding enantioselective hydroborations, chiral aluminum magnesium complexes display similar reactivity. However, chiral f-block metal complexes are still underdeveloped.

Furthermore, new catalyst-free protocols have recently appeared in the literature for the hydroboration of several unsaturated compounds. However, in almost all cases, excess of pinacolborane, elevated temperatures, or longer reaction times are required.

Given that only very few examples of enantioselective s-block metal-catalyzed hydroboration reactions are known, there are many opportunities for further developments in this field, and we anticipate that future directions will focus on enantioselective hydroboration of other unsaturated bonds, such as imines and alkenes, to achieve chiral amines and alkyl boranes. Moreover, we foresee that new research will also be directed toward other enantioselective hydrofunctionalizations.

Apart from the hydrofunctionalization of unsaturated bonds, and due to the high reactivity of s-block metals (and their low-valent analogues), we also expect the application of alkali and alkaline earth metal catalysts to further cutting-edge catalytic transformations such as C–H or C–X bond activation and functionalization. In this regard, to date, only stoichiometric use has been reported, and the need for catalytic transformations will provide important impetus for this area of research.

## Abbreviations

Ae: alkaline earth metals
atm: atmosphere
BINOL: 1,1′-bi-2-naphthol
Bn: benzyl group
Boc: *tert*-butyloxycarbonyl group
Box: bisoxazoline ligand
Boxmi: bis(oxazolinylmethylidene)isoindolines
Bu: butyl group
cat: catalyst
Cy: cyclohexyl group
DFT: density functional theory
DHP: dihydropyridine
Dipp: 2,6-diisopropylphenyl group
DMF: *N*,*N*-dimethylformamide
DPE: 1,1-diphenylethylene
dr: diastereomeric ratio
ee: enantiomeric excess
equiv: equivalents
er: enantiomeric ratio
Et: ethyl group
h: hours
HBcat: catecholborane
HBpin: pinacolborane
HMDS: hexamethyldisilazane
hpp: 1,3,4,6,7,8-hexahydro-2*H*-pyrimido[1,2-*a*]pyrimidinate
*i*: iso
KIE: kinetic isotope effect
LDA: lithium diisopropylamide
m: minutes
*m*: *meta*
M_4_TACD: tris{2-(dimethylamino)ethyl}amine
M_6_TREN: 1,4,7,10-tetramethyl-1,4,7,10-tetraazacyclododecane
Mes: mesityl (2,4,6-trimethylphenyl) group
MOF: metal–organic framework
NMR: nuclear magnetic resonance
*o*: *ortho*
OTf: trifluoromethanesulfonate group
*p*: *para*
Ph: phenyl group
pin: pinacol
Pr: propyl group
Py: pyridine
R: undefined group
rr: regioselective ratio
rt: room temperature
*t*: *tert*
THF: tetrahydrofuran
TismPriBenz: tris[(1-isopropylbenzimidazol-2-yl)dimethylsilyl)] methyl ligand
TMEDA: *N*,*N*,*N*′,*N*′-tetramethylethylenediamine
TMS: trimethylsilyl group
TOF: turnover frequency
ToM: tris(4,4-dimethyl-2-oxazolinyl)phenylborate)
TPHN: 4,4′-bis(carboxyphenyl)-2-nitro-1,1′-biphenyl
Xyl: xylyl (dimethylphenyl) group
